# Supplementary material for: A multi-phase project to develop a patient-reported measure of barriers to antiretroviral therapy adherence for use in HIV care: The 7-Item I-Score
Source: PLoS One. 2026 Jan 6;21(1):e0324241. doi: 10.1371/journal.pone.0324241 (PMC12774347; doi:10.1371/journal.pone.0324241)
Supplement: S1 File — (DOCX) [file pone.0324241.s001.docx]

**S1 File. Cognitive interview schedule for the focus groups.**

**General questions**

**Instructions**

- Are the instructions for filling out the questionnaire clear? Is there anything that you do not understand?

**Item by item**

- What do you think we want to know with this item?
- Is it hard to answer this question? (Is it mentally difficult or confusing?)
- How could we improve this item? If so, why?

**At the end of each questionnaire**

1. What do you think about the length of the questionnaire? Should it be shorter or longer?
2. Does the questionnaire seem to cover all relevant aspects? Was anything important left out?
3. Should the content be ordered differently (e.g., should some items come before others)?
4. Are the response options appropriate? (sliding scale from Never to Always)
5. Is it acceptable to ask people living with HIV these questions?
6. How would you feel if your healthcare providers received your responses to these questionnaires?

**Specific questions**

1. For clarity, should HIV be written every time we mention “medication” or “provider” or some other aspect that could be qualified by HIV? If so, why?
   1. Could another term be used instead?
2. What do you think of the timeframe for these questionnaires (past 4 weeks)? Should it be shorter (e.g., past day, past 7 days) or longer (e.g., past 12 weeks, past 6 months)? Why?
