# Supplementary material for: A multi-phase project to develop a patient-reported measure of barriers to antiretroviral therapy adherence for use in HIV care: The 7-Item I-Score
Source: PLoS One. 2026 Jan 6;21(1):e0324241. doi: 10.1371/journal.pone.0324241 (PMC12774347; doi:10.1371/journal.pone.0324241)
Supplement: S1 Table — (DOCX) [file pone.0324241.s002.docx]

**S1 Table. Descriptive statistics of the I-Score’s 7 items at each time point, for the global sample and stratified by sociodemographic group and mode of survey administration.**

|  |  | **Thoughts and feelings** | | **Habits and activities** | | **Social situation** | | **Economic situation** | | **Medication** | | **Care** | | **Health** | |
| --- | --- | --- | --- | --- | --- | --- | --- | --- | --- | --- | --- | --- | --- | --- | --- |
|  |  | Time 1 | Time 2 | Time 1 | Time 2 | Time 1 | Time 2 | Time 1 | Time 2 | Time 1 | Time 2 | Time 1 | Time 2 | Time 1 | Time 2 |
| **Global sample** (n=305) | Mean (SD)  Min - Max  n (missing) | 2.0 (2.8)  0.0 – 10.0  304 (1) | 2.0 (2.9)  0.0 – 10.0  244 (61) | 1.6 (2.6)  0.0 – 10.0  304 (1) | 1.7 (2.7)  0.0 – 10.0  241 (64) | 1.9 (2.9)  0.0 – 10.0  304 (1) | 1.9 (2.9)  0.0 – 10.0  244 (61) | 2.2 (3.2)  0.0 – 10.0  305 (0) | 2.3 (3.3)  0.0 – 10.0  243 (62) | 1.5 (2.5)  0.0 – 10.0  305 (0) | 1.7 (2.7)  0.0 – 10.0  245 (60) | 0.7 (1.7)  0.0 – 10.0  303 (2) | 1.1 (2.1)  0.0 – 10.0  245 (60) | 1.1 (2.2)  0.0 – 10.0  303 (2) | 1.3 (2.3)  0.0 – 10.0  241 (64) |
| **Survey language** |  |  |  |  |  |  |  |  |  |  |  |  |  |  |  |
| English (n=83) | Mean (SD) | 2.1 (2.8) | 2.1 (3.3) | 1.8 (2.8) | 1.8 (2.8) | 2.6 (3.6) | 2.6 (3.2) | 2.7 (3.6) | 2.7 (3.7) | 1.2 (2.5) | 1.2 (2.5) | 0.9 (1.7) | 0.9 (1.7) | 1.0 (2.2) | 1.0 (2.2) |
|  | Min - Max | 0.0 – 10.0 | 0.0 – 10.0 | 0.0 – 10.0 | 0.0 – 10.0 | 0.0 – 10.0 | 0.0 – 10.0 | 0.0 – 10.0 | 0.0 – 10.0 | 0.0 – 10.0 | 0.0 – 10.0 | 0.0 – 10.0 | 0.0 – 10.0 | 0.0 – 10.0 | 0.0 – 10.0 |
|  | n (missing) | 83 (0) | 63 (20) | 83 (0) | 63 (20) | 82 (1) | 63 (20) | 83 (0) | 62 (21) | 83 (0) | 63 (20) | 82 (1) | 63 (20) | 83 (0) | 63 (20) |
| French (n=222) | Mean (SD) | 2.0 (2.7) | 2.0 (2.7) | 1.5 (2.5) | 1.5 (2.5) | 1.7 (2.6) | 1.7 (2.7) | 2.0 (3.0) | 2.2 (3.1) | 1.6 (2.5) | 1.8 (2.8) | 0.7 (1.7) | 1.0 (2.0) | 1.1 (2.2) | 1.2 (2.1) |
|  | Min - Max | 0.0 – 10.0 | 0.0 – 10.0 | 0.0 – 10.0 | 0.0 – 10.0 | 0.0 – 10.0 | 0.0 – 10.0 | 0.0 – 10.0 | 0.0 – 10.0 | 0.0 – 10.0 | 0.0 – 10.0 | 0.0 – 10.0 | 0.0 – 10.0 | 0.0 – 10.0 | 0.0 – 10.0 |
|  | n (missing) | 221 (1) | 181 (41) | 221 (1) | 178 (44) | 222 (0) | 181 (41) | 222 (0) | 181 (41) | 222 (0) | 182 (40) | 221 (1) | 182 (40) | 220 (2) | 178 (44) |
| **Country of residence** |  |  |  |  |  |  |  |  |  |  |  |  |  |  |  |
| Canada (n=261) | Mean (SD) | 2.1 (2.9) | 2.1 (2.9) | 1.7 (2.7) | 1.8 (2.7) | 2.2 (3.1) | 2.1 (2.9) | 2.5 (3.3) | 2.6 (3.4) | 1.5 (2.6) | 1.7 (2.8) | 0.8 (1.8) | 1.2 (2.3) | 1.1 (2.2) | 1.4 (2.4) |
|  | Min - Max | 0.0 – 10.0 | 0.0 – 10.0 | 0.0 – 10.0 | 0.0 – 10.0 | 0.0 – 10.0 | 0.0 – 10.0 | 0.0 – 10.0 | 0.0 – 10.0 | 0.0 – 10.0 | 0.0 – 10.0 | 0.0 – 10.0 | 0.0 – 10.0 | 0.0 – 10.0 | 0.0 – 10.0 |
|  | n (missing) | 260 (1) | 207 (54) | 260 (1) | 204 (57) | 260 (1) | 207 (54) | 261 (0) | 206 (55) | 261 (0) | 208 (53) | 259 (2) | 208 (53) | 259 (2) | 204 (57) |
| France (n=41) | Mean (SD) | 1.0 (1.9) | 1.5 (2.1) | 1.2 (2.1) | 1.6 (2.1) | 0.5 (1.2) | 1.2 (2.2) | 0.3 (0.9) | 0.7 (1.7) | 0.8 (1.8) | 1.4 (2.7) | 0.2 (0.7) | 0.3 (0.8) | 1.0 (2.2) | 0.7 (1.6) |
|  | Min - Max | 0.0 – 7.0 | 0.0 – 7.0 | 0.0 – 9.0 | 0.0 – 7.0 | 0.0 – 5.0 | 0.0 – 9.0 | 0.0 – 5.0 | 0.0 – 8.0 | 0.0 – 10.0 | 0.0 – 10.0 | 0.0 – 3.0 | 0.0 – 4.0 | 0.0 – 10.0 | 0.0 – 5.0 |
|  | n (missing) | 41 (0) | 34 (7) | 41 (0) | 34 (7) | 41 (0) | 34 (7) | 41 (0) | 34 (7) | 41 (0) | 34 (7) | 41 (0) | 34 (7) | 41 (0) | 34 (7) |
| Other (n=2) | Mean (SD) | 2.5 (0.7) | 0.0 (0.0) | 1.0 (1.4) | 0.0 (0.0) | 1.5 (2.1) | 0.0 (0.0) | 6.0 (2.8) | 0.0 (0.0) | 3.5 (2.1) | 0.0 (0.0) | 1.0 (1.4) | 0 (0) | 1.0 (1.4) | 0.0 (0.0) |
|  | Min - Max | 2.0 – 3.0 | 0.0 – 0.0 | 0.0 – 2.0 | 0.0 – 0.0 | 0.0 – 3.0 | 0.0 – 0.0 | 4.0 – 8.0 | 0.0 – 0.0 | 2.0 – 5.0 | 0.0 – 0.0 | 0.0 – 2.0 | 0.0 – 0.0 | 0.0 – 2.0 | 0.0 – 0.0 |
|  | n (missing) | 2 (0) | 2 (0) | 2 (0) | 2 (0) | 2 (0) | 2 (0) | 2 (0) | 2 (0) | 2 (0) | 2 (0) | 2 (0) | 2 (0) | 2 (0) | 2 (0) |
| **Immigration status** |  |  |  |  |  |  |  |  |  |  |  |  |  |  |  |
| Immigrant (n=161) | Mean (SD) | 2.2 (2.8) | 2.5 (3.2) | 1.6 (2.7) | 1.8 (2.9) | 2.3 (3.2) | 2.2 (2.9) | 2.8 (3.4) | 3.2 (3.5) | 1.8 (2.8) | 2.0 (2.9) | 0.9 (2.0) | 1.4 (2.5) | 1.2 (2.4) | 1.7 (2.7) |
|  | Min - Max | 0.0 – 10.0 | 0.0 – 10.0 | 0.0 – 10.0 | 0.0 – 10.0 | 0.0 – 10.0 | 0.0 – 10.0 | 0.0 – 10.0 | 0.0 – 10.0 | 0.0 – 10.0 | 0.0 – 10.0 | 0.0 – 10.0 | 0.0 – 10.0 | 0.0 – 10.0 | 0.0 – 10.0 |
|  | n (missing) | 160 (1) | 117 (44) | 160 (1) | 116 (45) | 160 (1) | 118 (43) | 161 (0) | 117 (44) | 161 (0) | 118 (43) | 159 (2) | 118 (43) | 161 (0) | 115 (46) |
| Non-immigrant (n=142) | Mean (SD) | 1.8 (2.6) | 1.6 (2.5) | 1.6 (2.5) | 1.6 (2.4) | 1.5 (2.5) | 1.7 (2.7) | 1.6 (2.9) | 1.4 (2.8) | 1.0 (2.1) | 1.3 (2.5) | 0.6 (1.2) | 0.7 (1.6) | 0.9 (2.0) | 1.0 (1.8) |
|  | Min - Max | 0.0 – 10.0 | 0.0 – 10.0 | 0.0 – 10.0 | 0.0 – 10.0 | 0.0 – 10.0 | 0.0 – 10.0 | 0.0 – 10.0 | 0.0 – 10.0 | 0.0 – 10.0 | 0.0 – 10.0 | 0.0 – 7.0 | 0.0 – 8.0 | 0.0 – 10.0 | 0.0 – 7.0 |
|  | n (missing) | 142 (0) | 125 (17) | 142 (0) | 123 (19) | 142 (0) | 124 (18) | 142 (0) | 124 (18) | 142 (0) | 125 (17) | 142 (0) | 125 (17) | 140 (2) | 124 (18) |
| **Age (years)** |  |  |  |  |  |  |  |  |  |  |  |  |  |  |  |
| < 50 (n=135) | Mean (SD) | 2.3 (2.8) | 2.2 (3.2) | 1.9 (2.7) | 2.2 (3.0) | 2.3 (3.3) | 1.9 (2.7) | 2.6 (3.5) | 2.5 (3.6) | 1.6 (2.6) | 1.6 (2.8) | 0.7 (1.5) | 1.0 (2.2) | 1.2 (2.2) | 1.3 (2.5) |
|  | Min - Max | 0.0 – 10.0 | 0.0 – 10.0 | 0.0 – 10.0 | 0.0 – 10.0 | 0.0 – 10.0 | 0.0 – 10.0 | 0.0 – 10.0 | 0.0 – 10.0 | 0.0 – 10.0 | 0.0 – 10.0 | 0.0 – 10.0 | 0.0 – 10.0 | 0.0 – 10.0 | 0.0 – 10.0 |
|  | n (missing) | 135 (0) | 107 (28) | 135 (0) | 105 (30) | 134 (1) | 106 (29) | 135 (0) | 106 (29) | 135 (0) | 107 (28) | 133 (2) | 107 (28) | 134 (1) | 106 (29) |
| ≥ 50 (n=160) | Mean (SD) | 1.8 (2.7) | 1.8 (2.5) | 1.4 (2.5) | 1.4 (2.3) | 1.7 (2.7) | 1.9 (2.9) | 1.8 (2.9) | 2.1 (3.0) | 1.2 (2.2) | 1.8 (2.7) | 0.7 (1.8) | 1.1 (2.1) | 1.0 (2.2) | 1.3 (2.0) |
|  | Min - Max | 0.0 – 10.0 | 0.0 – 10.0 | 0.0 – 10.0 | 0.0 – 10.0 | 0.0 – 10.0 | 0.0 – 10.0 | 0.0 – 10.0 | 0.0 – 10.0 | 0.0 – 10.0 | 0.0 – 10.0 | 0.0 – 10.0 | 0.0 – 10.0 | 0.0 – 10.0 | 0.0 – 10.0 |
|  | n (missing) | 159 (1) | 129 (31) | 159 (1) | 127 (33) | 160 (0) | 129 (31) | 160 (0) | 128 (32) | 160 (0) | 129 (31) | 160 (0) | 129 (31) | 159 (1) | 126 (34) |
| **Level of education** |  |  |  |  |  |  |  |  |  |  |  |  |  |  |  |
| Primary/Elementary (n=19) | Mean (SD) | 1.3 (2.4) | 1.8 (2.0) | 1.6 (2.9) | 2.4 (3.6) | 1.6 (2.7) | 1.2 (2.0) | 1.4 (2.6) | 1.3 (2.3) | 1.4 (2.9) | 1.5 (2.6) | 0.4 (1.2) | 0.4 (0.8) | 0.6 (1.3) | 2.0 (2.3) |
|  | Min - Max | 0.0 – 8.0 | 0.0 – 6.0 | 0.0 – 10.0 | 0.0 – 10.0 | 0.0 – 9.0 | 0.0 – 5.0 | 0.0 – 8.0 | 0.0 – 7.0 | 0.0 – 10.0 | 0.0 – 8.0 | 0.0 – 5.0 | 0.0 – 2.0 | 0.0 – 5.0 | 0.0 – 5.0 |
|  | n (missing) | 19 (0) | 13 (6) | 19 (0) | 13 (6) | 18 (1) | 13 (6) | 19 (0) | 13 (6) | 19 (0) | 13 (6) | 19 (0) | 13 (6) | 19 (0) | 12 (7) |
| Secondary (High school) / Professional degree (n=109) | Mean (SD) | 1.9 (2.6) | 1.9 (2.7) | 1.5 (2.6) | 1.2 (2.3) | 1.5 (2.5) | 1.9 (3.0) | 2.0 (3.0) | 2.3 (3.2) | 1.3 (2.3) | 1.5 (2.4) | 0.6 (1.4) | 0.8 (1.6) | 0.9 (2.1) | 1.2 (2.2) |
|  | Min - Max | 0.0 – 10.0 | 0.0 – 10.0 | 0.0 – 10.0 | 0.0 – 10.0 | 0.0 – 10.0 | 0.0 – 10.0 | 0.0 – 10.0 | 0.0 – 10.0 | 0.0 – 10.0 | 0.0 – 9.0 | 0.0 – 7.0 | 0.0 – 7.0 | 0.0 – 10.0 | 0.0 – 10.0 |
|  | n (missing) | 109 (0) | 82 (27) | 109 (0) | 82 (27) | 109 (0) | 82 (27) | 109 (0) | 83 (26) | 109 (0) | 83 (26) | 109 (0) | 83 (26) | 108 (1) | 83 (26) |
| College (post-secondary) /  CEGEP / Technical degree (n=59) | Mean (SD) | 2.6 (3.1) | 2.8 (3.5) | 1.9 (2.9) | 2.4 (3.2) | 2.6 (3.5) | 2.9 (3.1) | 3.2 (3.9) | 2.7 (3.4) | 1.9 (3.0) | 2.2 (3.1) | 1.0 (2.3) | 1.9 (2.9) | 1.8 (3.1) | 1.8 (2.7) |
|  | Min - Max | 0.0 – 10.0 | 0.0 – 10.0 | 0.0 – 10.0 | 0.0 – 10.0 | 0.0 – 10.0 | 0.0 – 10.0 | 0.0 – 10.0 | 0.0 – 10.0 | 0.0 – 10.0 | 0.0 – 10.0 | 0.0 – 10.0 | 0.0 – 10.0 | 0.0 – 10.0 | 0.0 – 10.0 |
|  | n (missing) | 59 (0) | 47 (12) | 58 (1) | 45 (14) | 59 (0) | 47 (12) | 59 (0) | 47 (12) | 59 (0) | 47 (12) | 57 (2) | 47 (12) | 58 (1) | 45 (14) |
| University (n=104) | Mean (SD) | 1.9 (2.8) | 1.6 (2.7) | 1.5 (2.3) | 1.8 (2.6) | 2.0 (3.0) | 1.6 (2.7) | 1.9 (3.0) | 2.1 (3.4) | 1.3 (2.3) | 1.6 (2.8) | 0.8 (1.6) | 1.0 (2.0) | 1.0 (1.8) | 1.1 (2.2) |
|  | Min - Max | 0.0 – 10.0 | 0.0 – 10.0 | 0.0 – 9.0 | 0.0 – 10.0 | 0.0 – 10.0 | 0.0 – 10.0 | 0.0 – 10.0 | 0.0 – 10.0 | 0.0 – 10.0 | 0.0 – 10.0 | 0.0 – 10.0 | 0.0 – 10.0 | 0.0 – 8.0 | 0.0 – 10.0 |
|  | n (missing) | 103 (1) | 89 (15) | 104 (0) | 88 (16) | 104 (0) | 89 (15) | 104 (0) | 88 (16) | 104 (0) | 89 (15) | 104 (0) | 89 (15) | 104 (0) | 88 (16) |
| Other (n=8) | Mean (SD) | 2.2 (2.0) | 3.9 (3.0) | 2.6 (3.3) | 2.0 (2.5) | 2.2 (2.7) | 1.3 (2.2) | 3.5 (3.4) | 2.5 (3.7) | 2.8 (3.6) | 2.4 (3.6) | 0.4 (0.7) | 0.6 (1.1) | 1.0 (1.7) | 1.0 (2.6) |
|  | Min - Max | 0.0 – 6.0 | 2.0 – 10.0 | 0.0 – 10.0 | 0.0 – 7.0 | 0.0 – 8.0 | 0.0 – 6.0 | 0.0 – 8.0 | 0.0 – 9.0 | 0.0 – 9.0 | 0.0 – 10.0 | 0.0 – 2.0 | 0.0 – 3.0 | 0.0 – 5.0 | 0.0 – 7.0 |
|  | n (missing) | 8 (0) | 7 (1) | 8 (0) | 7 (1) | 8 (0) | 7 (1) | 8 (0) | 6 (2) | 8 (0) | 7 (1) | 8 (0) | 7 (1) | 8 (0) | 7 (1) |
| **Sex** |  |  |  |  |  |  |  |  |  |  |  |  |  |  |  |
| Female (n=94) | Mean (SD) | 2.3 (3.1) | 2.6 (3.2) | 2.0 (3.0) | 1.9 (3.0) | 2.1 (3.3) | 2.1 (3.0) | 2.8 (3.4) | 2.9 (3.6) | 1.9 (3.1) | 2.3 (3.2) | 1.0 (2.3) | 1.0 (2.2) | 1.5 (2.9) | 1.5 (2.5) |
|  | Min - Max | 0.0 – 10.0 | 0.0 – 10.0 | 0.0 – 10.0 | 0.0 – 10.0 | 0.0 – 10.0 | 0.0 – 10.0 | 0.0 – 10.0 | 0.0 – 10.0 | 0.0 – 10.0 | 0.0 – 10.0 | 0.0 – 10.0 | 0.0 – 10.0 | 0.0 – 10.0 | 0.0 – 10.0 |
|  | n (missing) | 93 (1) | 68 (26) | 93 (1) | 65 (29) | 93 (1) | 67 (27) | 94 (0) | 68 (26) | 94 (0) | 68 (26) | 92 (2) | 68 (26) | 93 (1) | 66 (28) |
| Male (n=207) | Mean (SD) | 1.8 (2.6) | 1.8 (2.7) | 1.4 (2.4) | 1.7 (2.5) | 1.8 (2.7) | 1.9 (2.8) | 1.9 (3.0) | 2.0 (3.0) | 1.2 (2.2) | 1.4 (2.5) | 0.6 (1.3) | 1.1 (2.1) | 0.9 (1.8) | 1.2 (2.1) |
|  | Min - Max | 0.0 – 10.0 | 0.0 – 10.0 | 0.0 – 10.0 | 0.0 – 10.0 | 0.0 – 10.0 | 0.0 – 10.0 | 0.0 – 10.0 | 0.0 – 10.0 | 0.0 – 10.0 | 0.0 – 10.0 | 0.0 – 6.0 | 0.0 – 10.0 | 0.0 – 10.0 | 0.0 – 10.0 |
|  | n (missing) | 207 (0) | 172 (35) | 207 (0) | 172 (35) | 207 (0) | 173 (34) | 207 (0) | 171 (36) | 207 (0) | 173 (34) | 207 (0) | 173 (34) | 206 (1) | 171 (36) |
| **Sexual orientation** |  |  |  |  |  |  |  |  |  |  |  |  |  |  |  |
| Heterosexual (n=119) | Mean (SD) | 1.9 (2.7) | 2.0 (2.8) | 1.5 (2.5) | 1.4 (2.3) | 1.6 (2.5) | 1.6 (2.6) | 2.2 (3.0) | 2.6 (3.2) | 1.5 (2.4) | 2.0 (2.8) | 0.9 (1.9) | 1.2 (2.3) | 1.1 (2.2) | 1.4 (2.4) |
|  | Min - Max | 0.0 – 10.0 | 0.0 – 10.0 | 0.0 – 10.0 | 0.0 – 10.0 | 0.0 – 10.0 | 0.0 – 10.0 | 0.0 – 10.0 | 0.0 – 10.0 | 0.0 – 10.0 | 0.0 – 10.0 | 0.0 – 10.0 | 0.0 – 10.0 | 0.0 – 10.0 | 0.0 – 10.0 |
|  | n (missing) | 118 (1) | 90 (29) | 118 (1) | 87 (32) | 118 (1) | 89 (30) | 119 (0) | 90 (29) | 119 (0) | 90 (29) | 117 (2) | 90 (29) | 118 (1) | 88 (31) |
| Homosexual (n=137) | Mean (SD) | 1.9 (2.6) | 1.8 (2.7) | 1.5 (2.2) | 1.8 (2.6) | 1.7 (2.7) | 2.0 (2.7) | 1.9 (3.0) | 1.7 (2.9) | 1.2 (2.2) | 1.5 (2.6) | 0.6 (1.2) | 0.9 (1.9) | 1.0 (1.9) | 1.2 (2.1) |
|  | Min - Max | 0.0 – 10.0 | 0.0 – 10.0 | 0.0 – 8.0 | 0.0 – 10.0 | 0.0 – 10.0 | 0.0 – 10.0 | 0.0 – 10.0 | 0.0 – 10.0 | 0.0 – 10.0 | 0.0 – 10.0 | 0.0 – 5.0 | 0.0 – 10.0 | 0.0 – 10.0 | 0.0 – 10.0 |
|  | n (missing) | 137 (0) | 121 (16) | 137 (0) | 121 (16) | 137 (0) | 122 (15) | 137 (0) | 120 (17) | 137 (0) | 122 (15) | 137 (0) | 122 (15) | 136 (1) | 121 (16) |
| Bisexual (n=22) | Mean (SD) | 1.8 (3.1) | 2.6 (3.6) | 2.2 (3.6) | 2.4 (3.4) | 3.2 (4.1) | 2.4 (3.3) | 2.8 (3.8) | 2.4 (3.6) | 1.6 (3.0) | 1.0 (2.5) | 0.6 (1.8) | 0.9 (1.7) | 1.0 (2.3) | 1.2 (2.4) |
|  | Min - Max | 0.0 – 10.0 | 0.0 – 10.0 | 0.0 – 10.0 | 0.0 – 10.0 | 0.0 – 10.0 | 0.0 – 9.0 | 0.0 – 10.0 | 0.0 – 10.0 | 0.0 – 10.0 | 0.0 – 10.0 | 0.0 – 7.0 | 0.0 – 6.0 | 0.0 – 8.0 | 0.0 – 7.0 |
|  | n (missing) | 22 (0) | 16 (6) | 22 (0) | 16 (6) | 22 (0) | 16 (6) | 22 (0) | 16 (6) | 22 (0) | 16 (6) | 22 (0) | 16 (6) | 22 (0) | 16 (6) |
| Other (n=5) | Mean (SD) | 3.0 (3.7) | 4.3 (5.1) | 6.0 (3.8) | 4.3 (5.1) | 5.8 (4.0) | 3.3 (5.8) | 6.8 (3.5) | 8.0 (3.5) | 3.2 (3.9) | 3.0 (5.2) | 1.4 (2.2) | 5.7 (5.1) | 1.8 (3.5) | 3.3 (5.8) |
|  | Min - Max | 0.0 – 9.0 | 0.0 – 10.0 | 2.0 – 10.0 | 0.0 – 10.0 | 1.0 – 10.0 | 0.0 – 10.0 | 3.0 – 10.0 | 4.0 – 10.0 | 0.0 – 10.0 | 0.0 – 9.0 | 0.0 – 5.0 | 0.0 – 10.0 | 0.0 – 8.0 | 0.0 – 10.0 |
|  | n (missing) | 5 (0) | 3 (2) | 5 (0) | 3 (2) | 5 (0) | 3 (2) | 5 (0) | 3 (2) | 5 (0) | 3 (2) | 5 (0) | 3 (2) | 5 (0) | 3 (2) |
| Prefer not to answer (n=18) | Mean (SD) | 2.2 (3.4) | 2.5 (4.1) | 1.6 (3.1) | 2.3 (4.1) | 2.3 (4.0) | 3.3 (4.6) | 2.6 (3.8) | 4.0 (4.8) | 1.7 (3.4) | 1.5 (3.2) | 0.8 (2.5) | 0.2 (0.4) | 1.9 (3.6) | 1.2 (3.1) |
|  | Min - Max | 0.0 – 10.0 | 0.0 – 10.0 | 0.0 – 10.0 | 0.0 – 10.0 | 0.0 – 10.0 | 0.0 – 10.0 | 0.0 – 10.0 | 0.0 – 10.0 | 0.0 – 10.0 | 0.0 – 10.0 | 0.0 – 10.0 | 0.0 – 1.0 | 0.0 – 10.0 | 0.0 – 10.0 |
|  | n (missing) | 18 (0) | 11 (7) | 18 (0) | 11 (7) | 18 (0) | 11 (7) | 18 (0) | 11 (7) | 18 (0) | 11 (7) | 18 (0) | 11 (7) | 18 (0) | 10 (7) |
| **Administration mode** |  |  |  |  |  |  |  |  |  |  |  |  |  |  |  |
| Face to face interview | Mean (SD) | 1.5 (2.3) | 2.0 (2.6) | 1.3 (2.6) | 1.5 (2.7) | 1.2 (2.1) | 1.7 (2.7) | 2.4 (3.0) | 3.0 (3.1) | 1.3 (2.1) | 1.8 (2.5) | 0.7 (1.7) | 0.9 (1.9) | 0.8 (1.9) | 0.9 (1.7) |
|  | Min - Max | 0.0 – 10.0 | 0.0 – 10.0 | 0.0 – 10.0 | 0.0 – 10.0 | 0.0 – 10.0 | 0.0 – 10.0 | 0.0 – 10.0 | 0.0 – 10.0 | 0.0 – 10.0 | 0.0 – 10.0 | 0.0 – 10.0 | 0.0 – 10.0 | 0.0 – 10.0 | 0.0 – 7.0 |
|  | n (missing) | 69 (0) | 47 (6) | 69 (0) | 47 (6) | 69 (0) | 47 (6) | 69 (0) | 48 (5) | 69 (0) | 48 (5) | 69 (0) | 48 (5) | 69 (0) | 46 (7) |
| Self-administration online | Mean (SD) | 1.9 (2.7) | 2.0 (2.9) | 1.7 (2.6) | 1.7 (2.5) | 2.0 (3.0) | 1.8 (2.8) | 2.0 (3.2) | 2.0 (3.2) | 1.4 (2.5) | 1.6 (2.7) | 0.8 (1.5) | 1.0 (2.0) | 1.1 (2.1) | 1.4 (2.4) |
|  | Min - Max | 0.0 – 10.0 | 0.0 – 10.0 | 0.0 – 10.0 | 0.0 – 10.0 | 0.0 – 10.0 | 0.0 – 10.0 | 0.0 – 10.0 | 0.0 – 10.0 | 0.0 – 10.0 | 0.0 – 10.0 | 0.0 – 10.0 | 0.0 – 10.0 | 0.0 – 10.0 | 0.0 – 10.0 |
|  | n (missing) | 184 (0) | 166 (0) | 183 (1) | 163 (3) | 183 (1) | 166 (0) | 184 (0) | 164 (2) | 184 (0) | 166 (0) | 183 (1) | 166 (0) | 182 (2) | 164 (2) |
| Self-administration on paper | Mean (SD) | 2.7 (3.0) | 2.2 (3.2) | 1.4 (2.4) | 1.9 (3.3) | 2.5 (3.5) | 3.7 (3.8) | 2.4 (3.3) | 3.7 (4.2) | 1.6 (2.9) | 1.9 (3.2) | 0.8 (2.1) | 2.3 (3.2) | 1.3 (2.7) | 1.8 (3.0) |
|  | Min - Max | 0.0 – 10.0 | 0.0 – 10.0 | 0.0 – 10.0 | 0.0 – 10.0 | 0.0 – 10.0 | 0.0 – 10.0 | 0.0 – 10.0 | 0.0 – 10.0 | 0.0 – 10.0 | 0.0 – 10.0 | 0.0 – 10.0 | 0.0 – 10.0 | 0.0 – 10.0 | 0.0 – 10.0 |
|  | n (missing) | 44 (1) | 19 (1) | 45 (0) | 19 (1) | 45 (0) | 19 (1) | 45 (0) | 19 (1) | 45 (0) | 19 (1) | 44 (1) | 19 (1) | 45 (0) | 19 (1) |
| Phone interview | Mean (SD) | - | 1.0 (1.4) | - | 0 (0) | - | 0 (0) | - | 1.5 (2.1) | - | 0 (0) | - | 0 (0) | - | 0 (0) |
|  | Min - Max | - | 0.0 – 2.0 | - | 0.0 – 0.0 | - | 0.0 – 0.0 | - | 0.0 – 3.0 | - | 0.0 – 0.0 | - | 0.0 – 0.0 | - | 0.0 – 0.0 |
|  | n (missing) | - | 2 (1) | - | 2 (1) | - | 2 (1) | - | 2 (1) | - | 2 (1) | - | 2 (1) | - | 2 (1) |

*Note*. Time 1 = baseline, Time 2 = 4 weeks later.
