# Supplementary material for: A multi-phase project to develop a patient-reported measure of barriers to antiretroviral therapy adherence for use in HIV care: The 7-Item I-Score
Source: PLoS One. 2026 Jan 6;21(1):e0324241. doi: 10.1371/journal.pone.0324241 (PMC12774347; doi:10.1371/journal.pone.0324241)
Supplement: S2 Table — (DOCX) [file pone.0324241.s003.docx]

**S2 Table. Descriptive statistics of the five dependent variables at each time point, for the global sample and stratified by sociodemographic group and mode of survey administration.**

|  |  | **ART adherence: last 30 days** | | | **ART adherence: last 7 days** | | | **Intention to adhere to ART** | | | **Viral load: self-reported** | | | **Viral load: plasma** | | | |
| --- | --- | --- | --- | --- | --- | --- | --- | --- | --- | --- | --- | --- | --- | --- | --- | --- | --- |
|  |  | ≥ 95% | < 95% | Missing | All pills taken | ≥ 1 missed pill | Missing | M=5 | M<5 | Missing | Undetec | Not  undetec | Missing | | Undetec | Not  undetec | Missing |
| **Global sample** (n=305) | Time 1 | 222 (72.8%)  176 (57.7%) | 69 (22.6%)  57 (18.7%) | 14  (4.6%)  72  (23.6%) | 264 (86.6%)  201 (65.9%) | 40  (13.1%)  43  (14.1%) | 1  (0.3%)  61  (20.0%) | 189 (62.0%)  153 (50.2%) | 114 (37.4%)  92 (30.2%) | 2  (0.6%)  60  (19.7%) | 266 (87.2%)  223 (73.1%) | 24  (7.9%)  13  (4.3%) | 15  (4.9%)  69  (22.6%) | | 93 (30.5%)  62 (20.3%) | 17  (5.6%)  10  (3.3%) | 195  (63.9%)  233  (76.4%) |
|  | Time 2 |  |  |  |  |  |  |  |  |  |  |  |  |  |  |  |  |
| **Survey language** |  |  |  |  |  |  |  |  |  |  |  |  |  | |  |  |  |
| English (n=83) | Time 1 | 66  (79.5%) | 15  (18.1%) | 2  (2.4%) | 68  (81.9%) | 15  (18.1%) | 0  (0.0%) | 56  (67.5%) | 27  (32.5%) | 0  (0.0%) | 71  (85.5%) | 7  (8.4%) | 5  (6.1%) | | 34  (41.0%) | 2  (2.4%) | 47  (56.6%) |
|  | Time 2 | 45  (54.2%) | 15  (18.1%) | 23  (27.7%) | 52  (62.7%) | 11  (13.3%) | 20  (24.1%) | 48  (57.8%) | 15  (18.1%) | 20  (24.1%) | 56  (67.5%) | 4  (4.8%) | 23  (27.7%) | | 25  (30.1%) | 2  (2.4%) | 56  (67.5%) |
| French (n=222) | Time 1 | 156 (70.3%) | 54 (24.3%) | 12  (5.4%) | 196 (88.3%) | 25  (11.3%) | 1  (0.4%) | 133 (59.9%) | 87 (39.2%) | 2  (0.9%) | 195 (87.8%) | 17  (7.7%) | 10  (4.5%) | | 59 (26.6%) | 15  (6.8%) | 148  (66.6%) |
|  | Time 2 | 131 (59.0%) | 42 (18.9%) | 49  (22.1%) | 149 (67.1%) | 32  (14.4%) | 41  (18.5%) | 105 (47.3%) | 77 (34.7%) | 40  (18.0%) | 167 (75.2%) | 9  (4.1%) | 46  (20.7%) | | 37 (16.7%) | 8  (3.6%) | 177  (79.7%) |
| **Country of residence** |  |  |  |  |  |  |  |  |  |  |  |  |  | |  |  |  |
| Canada (n=261) | Time 1 | 193 (73.9%) | 55 (21.1%) | 13  (5.0%) | 222 (85.1%) | 38  (14.6%) | 1  (0.3%) | 159 (60.9%) | 100 (38.3%) | 2  (0.8%) | 226 (86.6%) | 20  (7.7%) | 15  (5.7%) | | 93 (35.6%) | 17  (6.5%) | 151  (57.9%) |
|  | Time 2 | 150 (57.5%) | 48 (18.4%) | 63  (24.1%) | 170 (65.1%) | 38  (14.6%) | 53  (20.3%) | 127 (48.7%) | 82 (31.4%) | 52  (19.9%) | 187 (71.6%) | 12  (4.6%) | 62  (23.8%) | | 62 (23.8%) | 10  (3.8%) | 189  (72.4%) |
| France (n=41) | Time 1 | 27 (65.9%) | 14 (34.1%) | 0  (0.0%) | 39  (95.1%) | 2  (4.9%) | 0  (0.0%) | 28 (68.3%) | 13 (31.7%) | 0  (0.0%) | 37 (90.2%) | 4  (9.8%) | 0  (0.0%) | | 0  (0.0%) | 0  (0.0%) | 0  (0.0%) |
|  | Time 2 | 25 (61.0%) | 8 (19.5%) | 8  (19.5%) | 30  (73.2%) | 4  (9.8%) | 7  (17.0%) | 25 (61.0%) | 9 (22.0%) | 7  (17.0%) | 33 (80.5%) | 1  (2.5%) | 7  (17.0%) | | 0  (0.0%) | 0  (0.0%) | 0  (0.0%) |
| Other (n=2) | Time 1 | 1  (50.0%) | 0  (0.0%) | 1  (50.0%) | 2  (100.0%) | 0  (0.0%) | 0  (0.0%) | 1  (50.0%) | 1  (50.0%) | 0  (0.0%) | 0  (0.0%) | 2  (100.0%) | 0  (0.0%) | | 0  (0.0%) | 0  (0.0%) | 0  (0.0%) |
|  | Time 2 | 1  (50.0%) | 1  (50.0%) | 0  (0.0%) | 1  (50.0%) | 1  (50.0%) | 0  (0.0%) | 1  (50.0%) | 1  (50.0%) | 0  (0.0%) | 0  (0.0%) | 2  (100.0%) | 0  (0.0%) | | 0  (0.0%) | 0  (0.0%) | 0  (0.0%) |
| **Immigration status** |  |  |  |  |  |  |  |  |  |  |  |  |  | |  |  |  |
| Immigrant (n=161) | Time 1 | 114 (70.8%) | 37 (23.0%) | 10  (6.2%) | 142 (88.2%) | 19  (11.8%) | 0  (0.0%) | 93 (57.8%) | 66 (41.0%) | 2  (1.2%) | 139 (86.4%) | 11  (6.8%) | 11  (6.8%) | | 59 (36.6%) | 13  (8.1%) | 89  (55.3%) |
|  | Time 2 | 87 (54.0%) | 23 (14.3%) | 51  (31.7%) | 99  (61.5%) | 19  (11.8%) | 43  (26.7%) | 64 (39.8%) | 55 (34.2%) | 42  (26.0%) | 108 (67.1%) | 7  (4.3%) | 46  (28.6%) | | 35 (21.8%) | 6  (3.7%) | 120  (74.5%) |
| Non-immigrant (n=142) | Time 1 | 106 (74.7%) | 32 (22.5%) | 4  (2.8%) | 120 (84.5%) | 21  (14.8%) | 1  (0.7%) | 95 (66.9%) | 47 (33.1%) | 0  (0.0%) | 125 (88.0%) | 13  (9.2%) | 4  (2.8%) | | 34 (23.9%) | 4  (2.8%) | 104  (73.3%) |
|  | Time 2 | 88 (62.0%) | 34 (23.9%) | 20  (14.1%) | 101 (71.1%) | 24  (16.9%) | 17  (12.0%) | 89 (62.7%) | 36 (25.3%) | 17  (12.0%) | 113 (79.6%) | 6  (4.2%) | 23  (13.2%) | | 27 (19.0%) | 4  (2.8%) | 111  (78.2%) |
| **Age (years)** |  |  |  |  |  |  |  |  |  |  |  |  |  | |  |  |  |
| < 50 (n=135) | Time 1 | 96 (71.1%) | 32 (23.7%) | 7  (5.2%) | 116 (85.9%) | 19  (14.1%) | 0  (0.0%) | 81 (60.0%) | 54 (40.0%) | 0  (0.0%) | 118 (87.4%) | 11  (8.1%) | 6  (4.5%) | | 40 (29.6%) | 8  (5.9%) | 87  (64.5%) |
|  | Time 2 | 74 (54.8%) | 32 (23.7%) | 29  (21.5%) | 84  (62.2%) | 24  (17.8%) | 27  (20.0%) | 69 (51.1%) | 38 (28.1%) | 28  (20.8%) | 101 (74.8%) | 5  (3.7%) | 29  (21.5%) | | 32 (23.7%) | 2  (1.5%) | 101  (74.8%) |
| ≥ 50 (n=160) | Time 1 | 119 (74.4%) | 36 (22.5%) | 5  (3.1%) | 138 (86.3%) | 21  (13.1%) | 1  (0.6%) | 102 (63.7%) | 56 (35.0%) | 2  (1.3%) | 140 (87.5%) | 12  (7.5%) | 8  (5.0%) | | 49 (30.6%) | 9  (5.6%) | 102  (63.8%) |
|  | Time 2 | 95 (59.4%) | 24 (15.0%) | 41  (25.6%) | 110 (68.8%) | 18  (11.2%) | 32  (20.0%) | 81 (50.6%) | 49 (30.6%) | 30  (18.8%) | 115 (71.9%) | 6  (3.7%) | 39  (24.4%) | | 29 (18.1%) | 8  (5.0%) | 123  (76.9%) |
| **Level of education** |  |  |  |  |  |  |  |  |  |  |  |  |  | |  |  |  |
| Primary/Elementary  (n=19) | Time 1 | 10 (52.6%) | 7 (36.8%) | 2  (10.6%) | 14  (73.7%) | 5  (26.3%) | 0  (0.0%) | 6 (31.6%) | 12 (63.1%) | 1  (5.3%) | 12 (63.1%) | 6  (31.6%) | 1  (5.3%) | | 4 (21.1%) | 3  (15.8%) | 12  (63.1%) |
|  | Time 2 | 8 (42.1%) | 5 (26.3%) | 6  (31.6%) | 9  (47.3%) | 4  (21.1%) | 6  (31.6%) | 6 (31.6%) | 7 (36.8%) | 6  (31.6%) | 11 (57.9%) | 2  (10.5%) | 6  (31.6%) | | 1  (5.3%) | 1  (5.3%) | 17  (89.4%) |
| Secondary (High school) /Professional degree (n=109) | Time 1 | 81 (74.3%) | 24 (22.0%) | 4  (3.7%) | 95  (87.2%) | 13  (11.9%) | 1  (0.9%) | 65 (59.6%) | 43 (39.5%) | 1  (0.9%) | 96 (88.1%) | 7  (6.4%) | 6  (5.5%) | | 33 (30.3%) | 4  (3.7%) | 72  (66.0%) |
|  | Time 2 | 64 (58.7%) | 12 (11.0%) | 33  (30.3%) | 74  (67.9%) | 10  (9.2%) | 25  (22.9%) | 52 (47.7%) | 32 (29.4%) | 25  (22.9%) | 78 (71.5%) | 4  (3.7%) | 27  (24.8%) | | 22 (20.2%) | 3  (2.7%) | 84  (77.1%) |
| College (post-secondary) /  CEGEP / Technical degree (n=59) | Time 1 | 41 (69.5%) | 16 (27.1%) | 2  (3.4%) | 47  (79.7%) | 12  (20.3%) | 0  (0.0%) | 36 (61.0%) | 23 (39.0%) | 0  (0.0%) | 51 (86.4%) | 3  (5.1%) | 5  (8.5%) | | 20 (33.9%) | 2  (3.4%) | 37  (62.7%) |
|  | Time 2 | 30 (50.9%) | 13 (22.0%) | 16  (27.1%) | 34  (57.7%) | 12  (20.3%) | 13  (22.0%) | 27 (45.8%) | 20 (33.9%) | 12  (20.3%) | 39 (66.1%) | 3  (5.1%) | 17  (28.8%) | | 11 (18.6%) | 1  (1.7%) | 47  (79.7%) |
| University (n=104) | Time 1 | 80 (76.9%) | 18 (17.3%) | 6  (5.8%) | 96  (92.3%) | 8  (7.7%) | 0  (0.0%) | 74 (71.2%) | 30 (28.8%) | 0  (0.0%) | 95 (91.4%) | 7  (6.7%) | 2  (1.9%) | | 34 (32.7%) | 8  (7.7%) | 62  (59.6%) |
|  | Time 2 | 67 (64.4%) | 22 (21.2%) | 15  (14.4%) | 76  (73.1%) | 13  (12.5%) | 15  (14.4%) | 63 (60.6%) | 26 (25.0%) | 15  (14.4%) | 84 (80.8%) | 2  (1.9%) | 18  (17.3%) | | 26 (25.0%) | 5  (4.8%) | 73  (70.2%) |
| Other (n=8) | Time 1 | 5 (62.5%) | 3 (37.5%) | 0  (0.0%) | 7  (87.5%) | 1  (12.5%) | 0  (0.0%) | 4 (50.0%) | 4 (50.0%) | 0  (0.0%) | 7 (87.5%) | 1  (12.5%) | 0  (0.0%) | | 1 (12.5%) | 0  (0%) | 7  (87.5%) |
|  | Time 2 | 4 (50.0%) | 3 (37.5%) | 1  (12.5%) | 5  (62.5%) | 2  (25.0%) | 1  (12.5%) | 4 (50.0%) | 3 (37.5%) | 1  (12.5%) | 6 (75.0%) | 1  (12.5%) | 1  (12.5%) | | 1 (12.5%) | 0  (0%) | 7  (87.5%) |
| **Sex** |  |  |  |  |  |  |  |  |  |  |  |  |  | |  |  |  |
| Female (n=94) | Time 1 | 59 (62.8%) | 25 (26.6%) | 10  (10.6%) | 78  (83.0%) | 16  (17.0%) | 0  (0.0%) | 47 (50.0%) | 46 (48.9%) | 1  (1.1%) | 76 (80.9%) | 11 (11.7%) | 7  (7.4%) | | 28 (29.8%) | 3  (3.2%) | 63  (67.0%) |
|  | Time 2 | 42 (44.7%) | 20 (21.3%) | 32  (34.0%) | 50  (53.2%) | 16  (17.0%) | 28  (29.8%) | 33 (35.1%) | 35 (37.2%) | 26  (27.7%) | 56 (59.6%) | 8  (8.5%) | 30  (31.9%) | | 12 (12.8%) | 2  (2.1%) | 80  (85.1%) |
| Male (n=207) | Time 1 | 160 (77.3%) | 43 (20.8%) | 4  (1.9%) | 183 (88.4%) | 23  (11.1%) | 1  (0.5%) | 139 (67.1%) | 67 (32.4%) | 1  (0.5%) | 186 (89.8%) | 13  (6.3%) | 8  (3.9%) | | 63 (30.4%) | 14  (6.8%) | 130  (62.8%) |
|  | Time 2 | 132 (63.8%) | 36 (17.4%) | 39  (18.8%) | 149 (72.0%) | 26  (12.6%) | 32  (15.4%) | 118 (57.0%) | 56 (27.1%) | 33  (15.9%) | 163 (78.8%) | 5  (2.4%) | 39  (18.8%) | | 48 (23.2%) | 8  (3.9%) | 151  (72.9%) |
| **Sexual orientation** |  |  |  |  |  |  |  |  |  |  |  |  |  | |  |  |  |
| Heterosexual (n=119) | Time 1 | 81 (68.1%) | 34 (28.6%) | 4  (3.4%) | 101 (84.9%) | 18  (15.1%) | 0  (0.0%) | 62 (52.1%) | 56 (47.1%) | 1  (0.8%) | 105 (88.2%) | 11  (9.3%) | 3  (2.5%) | | 31 (26.1%) | 5  (4.2%) | 83  (69.7%) |
|  | Time 2 | 66 (55.4%) | 19 (16.0%) | 34  (28.6%) | 74  (62.2%) | 16  (13.4%) | 29  (24.4%) | 45 (37.8%) | 46 (38.7%) | 28  (23.5%) | 82 (68.9%) | 6  (5.0%) | 31  (26.1%) | | 15 (12.6%) | 4  (3.4%) | 100  (84.0%) |
| Homosexual (n=137) | Time 1 | 110 (80.3%) | 23 (16.8%) | 4  (2.9%) | 122 (89.1%) | 14  (10.2%) | 1  (0.7%) | 95 (69.4%) | 41 (29.9%) | 1  (0.7%) | 124 (90.5%) | 10  (7.3%) | 3  (2.2%) | | 42 (30.6%) | 9  (6.6%) | 86  (62.8%) |
|  | Time 2 | 91 (66.4%) | 26 (19.0%) | 20  (14.6%) | 103 (75.1%) | 19  (13.9%) | 15  (11.0%) | 88 (64.2%) | 34 (24.8%) | 15  (11.0%) | 111 (81.0%) | 5  (3.7%) | 21  (15.3%) | | 36 (26.3%) | 5  (3.6%) | 96  (70.1%) |
| Bisexual (n=22) | Time 1 | 14 (63.6%) | 8 (36.4%) | 0  (0.0%) | 17  (77.3%) | 5  (22.7%) | 0  (0.0%) | 14 (63.6%) | 8 (36.4%) | 0  (0.0%) | 18 (81.8%) | 3  (13.7%) | 1  (4.5%) | | 5 (22.7%) | 1  (4.5%) | 16  (72.7%) |
|  | Time 2 | 10 (45.4%) | 6 (27.3%) | 6  (27.3%) | 11  (50.0%) | 5  (22.7%) | 6  (27.3%) | 11 (50.0%) | 5 (22.7%) | 6  (27.3%) | 14 (63.6%) | 2  (9.1%) | 6  (27.3%) | | 4 (18.2%) | 0  (0%) | 18  (81.8%) |
| Other (n=5) | Time 1 | 4 (80.0%) | 1 (20.0%) | 0  (0.0%) | 3  (60.0%) | 2  (40.0%) | 0  (0.0%) | 4 (80.0%) | 1 (20.0%) | 0  (0.0%) | 5 (100.0%) | 0  (0.0%) | 0  (0.0%) | | 2 (40.0%) | 0  (0.0%) | 3  (60.0%) |
|  | Time 2 | 0  (0.0%) | 3 (60.0%) | 2  (40.0%) | 2  (40.0%) | 1  (20.0%) | 2  (40.0%) | 2 (40.0%) | 1 (20.0%) | 2  (40.0%) | 3  (60.0%) | 0  (0.0%) | 2  (40.0%) | | 1 (20.0%) | 1  (20.0%) | 3  (60.0%) |
| Prefer not to answer  (n=18) | Time 1 | 11 (61.1%) | 2 (11.1%) | 5  (27.8%) | 18 (100.0%) | 0  (0.0%) | 0  (0.0%) | 11 (61.1%) | 7 (38.9%) | 0  (0.0%) | 10 (55.6%) | 0  (0.0%) | 8  (44.4%) | | 11 (61.1%) | 2  (11.1%) | 5  (27.8%) |
|  | Time 2 | 8 (44.4%) | 2 (11.1%) | 8  (44.4%) | 10  (55.6%) | 1  (5.5%) | 7  (38.9%) | 6 (33.3%) | 5 (27.8%) | 7  (38.9%) | 10 (55.6%) | 0  (0.0%) | 8  (44.4%) | | 5 (27.8%) | 0  (0.0%) | 13  (72.2%) |
| **Administration mode** |  |  |  |  |  |  |  |  |  |  |  |  |  | |  |  |  |
| Face to face interview | Time 1  (n=69) | 48 (69.6%) | 18 (26.1%) | 3  (4.3%) | 59  (85.5%) | 10  (14.5%) | 0  (0.0%) | 28 (40.6%) | 40 (58.0%) | 1  (1.4%) | 58 (84.1%) | 7  (10.1%) | 4  (5.8%) | | 13 (18.8%) | 2  (2.9%) | 54  (78.3%) |
|  | Time 2  (n=53) | 39 (73.6%) | 9 (17.0%) | 5  (9.4%) | 38  (71.7%) | 10  (18.9%) | 5  (9.4%) | 13 (24.5%) | 35 (66.1%) | 5  (9.4%) | 42 (79.3%) | 6  (11.3%) | 5  (9.4%) | | 2  (3.8%) | 0  (0.0%) | 51  (96.2%) |
| Self-administration online | Time 1  (n=184) | 138 (75.0%) | 42 (22.8%) | 4  (2.2%) | 159 (86.4%) | 25  (13.6%) | 0  (0.0%) | 124 (67.4%) | 60 (32.6%) | 0  (0.0%) | 163 (88.6%) | 13  (7.1%) | 8  (4.3%) | | 53 (28.8%) | 9  (4.9%) | 122  (66.3%) |
|  | Time 2  (n=166) | 110 (66.3%) | 44 (26.5%) | 12  (7.2%) | 138 (83.1%) | 27  (16.3%) | 1  (0.6%) | 116 (69.9%) | 50 (30.1%) | 0  (0.0%) | 151 (91.0%) | 7  (4.2%) | 8  (4.8%) | | 57 (34.3%) | 8  (4.8%) | 101  (60.9%) |
| Self-administration  on paper | Time 1  (n=45) | 29 (64.4%) | 9 (20.0%) | 7  (15.6%) | 39  (86.7%) | 5  (11.1%) | 1  (2.2%) | 33 (73.3%) | 11 (24.5%) | 1  (2.2%) | 38 (84.4%) | 4  (8.9%) | 3  (6.7%) | | 25 (55.6%) | 6  (13.3%) | 14  (31.1%) |
|  | Time 2  (n=20) | 17 (85.0%) | 2 (10.0%) | 1  (5.0%) | 14 (70.0%) | 5  (25.0%) | 1  (5.0%) | 14 (70.0%) | 5 (25.0%) | 1  (5.0%) | 19 (95.0%) | 0  (0.0%) | 1  (5.0%) | | 2 (10.0%) | 1  (5.0%) | 17  (85.0%) |
| Phone interview | Time 1  (n=0) | 0  (0.0%) | 0  (0.0%) | 0  (0.0%) | 0  (0.0%) | 0  (0.0%) | 0  (0.0%) | 0  (0.0%) | 0  (0.0%) | 0  (0.0%) | 0  (0.0%) | 0  (0.0%) | 0  (0.0%) | | 0  (0.0%) | 0  (0.0%) | 0  (0.0%) |
|  | Time 2  (n=3) | 0  (0.0%) | 2  (66.7%) | 1  (33.3%) | 1  (33.3%) | 1  (33.3%) | 1  (33.3%) | 1  (33.3%) | 1  (33.3%) | 1  (33.3%) | 0  (0.0%) | 2  (66.7%) | 1  (33.3%) | | 1  (33.3%) | 1  (33.3%) | 1  (33.3%) |

*Note*. Time 1 = baseline, Time 2 = 4 weeks later. ART: antiretroviral therapy; M: Mean; Undetec: undetectable viral load.
