# Supplementary material for: A multi-phase project to develop a patient-reported measure of barriers to antiretroviral therapy adherence for use in HIV care: The 7-Item I-Score
Source: PLoS One. 2026 Jan 6;21(1):e0324241. doi: 10.1371/journal.pone.0324241 (PMC12774347; doi:10.1371/journal.pone.0324241)
Supplement: S3 Table — (DOCX) [file pone.0324241.s004.docx]

**S3 Table. Loadings at Baseline (Time 1) Between the Seven Principal Components Obtained by a Varimax Rotation and the I-Score’s Seven Items.**

|  | PC1 | PC2 | PC3 | PC4 | PC5 | PC6 | PC7 |
| --- | --- | --- | --- | --- | --- | --- | --- |
| Thoughts and feelings | 0.27 | 0.21 | 0.25 | 0.20 | 0.17 | 0.19 | **0.84** |
| Habits and activities | 0.13 | 0.11 | **0.95** | 0.11 | 0.10 | 0.13 | 0.18 |
| Social situation | **0.88** | 0.14 | 0.16 | 0.16 | 0.18 | 0.25 | 0.24 |
| Economic situation | 0.23 | 0.11 | 0.14 | 0.19 | 0.15 | **0.91** | 0.16 |
| Medication | 0.15 | 0.21 | 0.12 | **0.92** | 0.13 | 0.19 | 0.16 |
| Care | 0.15 | 0.20 | 0.11 | 0.12 | **0.93** | 0.14 | 0.14 |
| Health | 0.13 | **0.92** | 0.12 | 0.21 | 0.21 | 0.11 | 0.17 |

*Note*. The highest loading of each principal component is indicated in bold.
