# Supplementary material for: A multi-phase project to develop a patient-reported measure of barriers to antiretroviral therapy adherence for use in HIV care: The 7-Item I-Score
Source: PLoS One. 2026 Jan 6;21(1):e0324241. doi: 10.1371/journal.pone.0324241 (PMC12774347; doi:10.1371/journal.pone.0324241)
Supplement: S5 Table — (DOCX) [file pone.0324241.s006.docx]

**S5 Table. Odds ratios for the seven covariates (with 95% confidence intervals) of the ten logistic regressions, for the global sample and stratified by sociodemographic group and mode of survey administration.**

|  | Covariate | **ART adherence:**  **last 30 days** | | **ART adherence:**  **last 7 days** | | **Intention to**  **adhere to ART** | | **Viral load:**  **self-reported** | | **Viral load:**  **plasma** | |
| --- | --- | --- | --- | --- | --- | --- | --- | --- | --- | --- | --- |
|  |  | Time 1 | Time 2 | Time 1 | Time 2 | Time 1 | Time 2 | Time 1 | Time 2 | Time 1 | Time 2 |
|  |  |  |  |  |  |  |  |  |  |  |  |
| **Global sample** (n=305) | Thoughts and feelings | 0.89 (0.77, 1.03) | 0.87 (0.73, 1.03) | 0.98 (0.82, 1.19) | 0.87 (0.73, 1.04) | 0.96 (0.85, 1.09) | 0.88 (0.75, 1.03) | 1.01 (0.81, 1.29) | 1.40 (0.93, 2.44) | 0.88 (0.66, 1.19) | 2.53 (1.11, 7.96) |
|  | Habits and activities | **0.86 (0.76, 0.97)** | **0.79 (0.68, 0.90)** | **0.80 (0.69, 0.91)** | 0.87 (0.76, 1.01) | 0.93 (0.83, 1.03) | 0.97 (0.85, 1.10) | **0.74 (0.62, 0.89)** | 0.81 (0.63, 1.06) | **0.73 (0.55, 0.94)** | 0.67 (0.40, 1.12) |
|  | Social situation | 1.11 (0.96, 1.29) | 1.04 (0.89, 1.24) | 0.93 (0.79, 1.09) | 1.01 (0.86, 1.21) | 1.02 (0.91, 1.14) | 1.15 (1.00, 1.35) | 1.15 (0.88, 1.58) | 1.00 (0.71, 1.51) | 0.90 (0.70, 1.17) | 1.07 (0.71, 2.03) |
|  | Economic situation | 1.01 (0.91, 1.14) | 1.01 (0.89, 1.16) | 1.05 (0.92, 1.23) | 1.00 (0.88, 1.15) | **0.91 (0.82, 0.99)** | 0.96 (0.86, 1.07) | 1.20 (0.96, 1.59) | 0.97 (0.76, 1.32) | 1.31 (0.95, 1.95) | 1.01 (0.62, 1.94) |
|  | Medication | 1.00 (0.87, 1.16) | 1.11 (0.93, 1.34) | 1.01 (0.85, 1.23) | 1.04 (0.87, 1.27) | 0.93 (0.83, 1.05) | 0.89 (0.77, 1.01) | 1.25 (0.95, 1.82) | 1.03 (0.79, 1.50) | 1.54 (0.93, 2.99) | 1.11 (0.70, 1.99) |
|  | Care | 0.93 (0.76, 1.13) | 0.94 (0.75, 1.18) | 0.84 (0.67, 1.05) | 0.84 (0.65, 1.06) | 1.05 (0.88, 1.26) | 0.92 (0.75, 1.12) | 1.12 (0.77, 1.79) | 1.68 (0.89, 5.11) | 1.14 (0.77, 1.98) | 1.14 (0.54, 2.84) |
|  | Health | 0.94 (0.81, 1.11) | 1.01 (0.84, 1.24) | 1.11 (0.90, 1.40) | 1.21 (0.97, 1.55) | 0.99 (0.86, 1.14) | 0.98 (0.83, 1.16) | **0.73 (0.59, 0.91)** | **0.75 (0.57, 0.97)** | 0.74 (0.48, 1.16) | **0.28 (0.09, 0.64)** |

|  | Covariate | **ART adherence:**  **last 30 days** | | **ART adherence:**  **last 7 days** | | **Intention to**  **adhere to ART** | | **Viral load:**  **self-reported** | | **Viral load:**  **plasma** | |
| --- | --- | --- | --- | --- | --- | --- | --- | --- | --- | --- | --- |
|  |  | Time 1 | Time 2 | Time 1 | Time 2 | Time 1 | Time 2 | Time 1 | Time 2 | Time 1 | Time 2 |
| **Survey language** |  |  |  |  |  |  |  |  |  |  |  |
| English (n=83) | Thoughts and feelings | **0.69 (0.48, 0.95)** | 0.91 (0.58, 1.42) | 0.85 (0.61, 1.17) | 1.21 (0.75, 2.16) | 0.99 (0.77, 1.27) | 0.98 (0.67, 1.45) | 0.68 (0.37, 1.17) | * | * | * |
|  | Habits and activities | **0.75 (0.58, 0.94)** | **0.55 (0.36, 0.77)** | **0.73 (0.57, 0.91)** | **0.51 (0.31, 0.74)** | **0.76 (0.61, 0.92)** | 1.05 (0.77, 1.52) | **0.68 (0.45, 0.99)** |  |  |  |
|  | Social situation | 1.28 (0.92, 1.94) | 1.38 (0.93, 2.46) | 1.06 (0.81, 1.48) | 1.13 (0.76, 1.93) | 0.89 (0.75, 1.08) | 1.40 (0.99, 2.20) | 1.63 (0.93, 3.34) |  |  |  |
|  | Economic situation | 0.90 (0.71, 1.17) | 0.76 (0.52, 1.02) | 0.90 (0.70, 1.15) | 0.74 (0.49, 1.02) | 0.98 (0.82, 1.16) | 0.78 (0.58, 1.01) | 1.08 (0.73, 1.95) |  |  |  |
|  | Medication | 1.04 (0.74, 1.50) | 1.24 (0.80, 2.18) | 0.93 (0.69, 1.29) | 1.18 (0.71, 2.08) | 1.10 (0.85, 1.47) | 0.81 (0.57, 1.16) | 0.95 (0.58, 1.65) |  |  |  |
|  | Care | 0.68 (0.42, 1.00) | 0.62 (0.36, 1.00) | 0.74 (0.49, 1.05) | **0.51 (0.27, 0.82)** | 1.00 (0.74, 1.40) | 1.11 (0.69, 2.13) | 1.04 (0.53, 2.42) |  |  |  |
|  | Health | 1.12 (0.80, 1.65) | 0.99 (0.53, 1.81) | 1.02 (0.72, 1.55) | 1.50 (0.79, 2.93) | 0.96 (0.71, 1.34) | 0.69 (0.40, 1.09) | 0.83 (0.43, 1.41) |  |  |  |
| French (n=222) | Thoughts and feelings | 0.90 (0.74, 1.09) | 0.81 (0.65, 1.00) | 0.98 (0.77, 1.28) | **0.75 (0.60, 0.94)** | 0.91 (0.76, 1.08) | 0.86 (0.71, 1.04) | 1.17 (0.85, 1.75) | 1.81 (0.97, 4.87) | 0.82 (0.54, 1.18) | 1.85 (0.66, 6.92) |
|  | Habits and activities | 0.89 (0.76, 1.04) | 0.86 (0.73, 1.02) | **0.82 (0.68, 0.99)** | 1.02 (0.85, 1.24) | 1.01 (0.88, 1.17) | 0.99 (0.85, 1.16) | **0.71 (0.55, 0.91)** | 0.81 (0.58, 1.21) | 0.81 (0.60, 1.07) | 0.72 (0.39, 1.44) |
|  | Social situation | 1.08 (0.89, 1.31) | 0.99 (0.81, 1.23) | 0.90 (0.72, 1.15) | 1.01 (0.81, 1.27) | 1.04 (0.89, 1.23) | 1.11 (0.92, 1.36) | 1.09 (0.77, 1.73) | 0.91 (0.49, 1.79) | 0.92 (0.66, 1.33) | 1.42 (0.59, 6.23) |
|  | Economic situation | 1.04 (0.91, 1.21) | 1.11 (0.95, 1.34) | 1.17 (0.95, 1.50) | 1.11 (0.94, 1.33) | 0.89 (0.78, 1.00) | 1.00 (0.88, 1.14) | 1.22 (0.89, 1.84) | 0.89 (0.64, 1.37) | 1.27 (0.84, 2.07) | 0.97 (0.48, 3.68) |
|  | Medication | 0.99 (0.85, 1.18) | 1.03 (0.85, 1.29) | 1.01 (0.80, 1.34) | 0.97 (0.79, 1.24) | 0.92 (0.79, 1.06) | 0.89 (0.75, 1.04) | 1.45 (0.96, 2.74) | 1.24 (0.83, 2.59) | 1.61 (0.92, 3.49) | 1.28 (0.69, 2.91) |
|  | Care | 1.03 (0.81, 1.33) | 1.04 (0.77, 1.41) | 0.85 (0.59, 1.20) | 0.88 (0.63, 1.22) | 1.08 (0.86, 1.36) | 0.83 (0.63, 1.07) | 1.03 (0.61, 2.08) | 1.22 (0.63, 3.69) | 1.05 (0.59, 2.02) | 1.27 (0.53, 3.93) |
|  | Health | 0.92 (0.77, 1.10) | 1.04 (0.84, 1.32) | 1.13 (0.86, 1.62) | 1.23 (0.94, 1.70) | 1.01 (0.85, 1.21) | 1.07 (0.89, 1.31) | **0.74 (0.56, 0.96)** | **0.74 (0.54, 0.99)** | 0.77 (0.48, 1.28) | **0.26 (0.06, 0.67)** |

|  | Covariate | **ART adherence:**  **last 30 days** | | **ART adherence:**  **last 7 days** | | **Intention to**  **adhere to ART** | | **Viral load:**  **self-reported** | | **Viral load:**  **plasma** | |
| --- | --- | --- | --- | --- | --- | --- | --- | --- | --- | --- | --- |
|  |  | Time 1 | Time 2 | Time 1 | Time 2 | Time 1 | Time 2 | Time 1 | Time 2 | Time 1 | Time 2 |
| **Country of residence** |  |  |  |  |  |  |  |  |  |  |  |
| Canada (n=261) | Thoughts and feelings | 0.89 (0.76, 1.04) | 0.90 (0.75, 1.08) | 0.97 (0.81, 1.18) | 0.88 (0.73, 1.06) | 0.97 (0.85, 1.11) | 0.91 (0.77, 1.07) | 1.02 (0.79, 1.33) | 1.48 (0.92, 2.86) | 0.88 (0.66, 1.19) | 2.53 (1.11, 7.96) |
|  | Habits and activities | **0.84 (0.73, 0.95)** | **0.74 (0.63, 0.86)** | **0.77 (0.67, 0.89)** | 0.86 (0.74, 1.01) | **0.89 (0.79, 0.99)** | 0.95 (0.83, 1.09) | **0.71 (0.58, 0.87)** | 0.77 (0.59, 1.02) | **0.73 (0.55, 0.94)** | 0.67 (0.40, 1.12) |
|  | Social situation | 1.10 (0.95, 1.30) | 1.04 (0.88, 1.25) | 0.93 (0.80, 1.10) | 0.99 (0.84, 1.19) | 1.01 (0.90, 1.13) | 1.16 (1.00, 1.37) | 1.11 (0.86, 1.54) | 1.01 (0.72, 1.56) | 0.90 (0.70, 1.17) | 1.07 (0.71, 2.03) |
|  | Economic situation | 0.97 (0.86, 1.10) | 0.99 (0.86, 1.14) | 1.06 (0.92, 1.24) | 0.98 (0.86, 1.13) | 0.91 (0.83, 1.01) | 0.94 (0.84, 1.05) | 1.24 (0.97, 1.68) | 0.97 (0.76, 1.33) | 1.31 (0.95, 1.95) | 1.01 (0.62, 1.94) |
|  | Medication | 1.05 (0.89, 1.24) | 1.23 (1.00, 1.56) | 1.11 (0.91, 1.39) | 1.20 (0.97, 1.54) | 0.97 (0.85, 1.10) | 0.91 (0.78, 1.06) | 1.49 (1.00, 2.47) | 1.12 (0.81, 1.76) | 1.54 (0.93, 2.99) | 1.11 (0.70, 1.99) |
|  | Care | 0.92 (0.75, 1.14) | 0.92 (0.72, 1.18) | 0.85 (0.67, 1.06) | 0.86 (0.66, 1.09) | 1.04 (0.87, 1.26) | 0.93 (0.75, 1.14) | 1.14 (0.78, 1.84) | 1.77 (0.90, 5.44) | 1.14 (0.77, 1.98) | 1.14 (0.54, 2.84) |
|  | Health | 0.96 (0.80, 1.16) | 0.99 (0.78, 1.26) | 1.04 (0.83, 1.33) | 1.10 (0.86, 1.44) | 1.00 (0.85, 1.18) | 1.01 (0.83, 1.24) | **0.65 (0.48, 0.86)** | **0.68 (0.47, 0.98)** | 0.74 (0.46, 1.16) | **0.28 (0.09, 0.64)** |
| France (n=41) | Thoughts and feelings | 0.60 (0.30, 1.08) | 0.92 (0.41, 2.38) | * | ***** | * | * | * | * | NA | NA |
|  | Habits and activities | 1.25 (0.81, 2.12) | 0.92 (0.53, 1.75) |  |  |  |  |  |  |  |  |
|  | Social situation | 1.33 (0.65, 3.12) | 0.52 (0.18, 1.24) |  |  |  |  |  |  |  |  |
|  | Economic situation | * | 0.54 (0.09, 2.52) |  |  |  |  |  |  |  |  |
|  | Medication | 0.76 (0.34, 1.15) | 0.31 (0.03, 1.94) |  |  |  |  |  |  |  |  |
|  | Care | 0.54 (0.11, 1.93) | * |  |  |  |  |  |  |  |  |
|  | Health | 0.79 (0.46, 1.14) | 1.30 (0.73, 3.60) |  |  |  |  |  |  |  |  |

|  | Covariate | **ART adherence:**  **last 30 days** | | **ART adherence:**  **last 7 days** | | **Intention to**  **adhere to ART** | | **Viral load:**  **self-reported** | | **Viral load:**  **plasma** | |
| --- | --- | --- | --- | --- | --- | --- | --- | --- | --- | --- | --- |
|  |  | Time 1 | Time 2 | Time 1 | Time 2 | Time 1 | Time 2 | Time 1 | Time 2 | Time 1 | Time 2 |
| **Immigration status** |  |  |  |  |  |  |  |  |  |  |  |
| Immigrant (n=161) | Thoughts and feelings | 0.90 (0.74, 1.11) | 0.85 (0.66, 1.08) | 1.06 (0.81, 1.43) | 0.82 (0.63, 1.07) | 1.11 (0.93, 1.33) | 0.90 (0.72, 1.12) | 1.04 (0.71, 1.70) | 1.17 (0.62, 3.01) | 1.01 (0.71, 1.44) | * |
|  | Habits and activities | 0.94 (0.80, 1.10) | 0.83 (0.68, 1.01) | 0.83 (0.68, 1.01) | 1.11 (0.87, 1.53) | 0.94 (0.81, 1.08) | 0.98 (0.82, 1.18) | **0.72 (0.53, 0.94)** | 1.32 (0.75, 5.85) | **0.74 (0.53, 0.98)** | 0.70 (0.32, 1.56) |
|  | Social situation | 1.16 (0.98, 1.42) | 1.01 (0.81, 1.27) | 0.91 (0.73, 1.13) | 1.07 (0.85, 1.40) | 1.06 (0.92, 1.23) | 1.22 (1.00, 1.50) | * | 1.25 (0.75, 2.95) | 0.82 (0.58, 1.14) | 1.05 (0.52, 3.14) |
|  | Economic situation | 0.96 (0.83, 1.11) | 1.07 (0.89, 1.31) | 1.01 (0.84, 1.24) | 1.00 (0.84, 1.22) | 0.88 (0.77, 1.00) | 0.96 (0.83, 1.11) | 1.41 (0.96, 2.85) | 0.91 (0.65, 1.39) | 1.35 (0.94, 2.20) | 1.10 (0.60, 2.70) |
|  | Medication | 0.93 (0.78, 1.11) | 1.05 (0.85, 1.36) | **0.79 (0.64, 0.98)** | 0.90 (0.73, 1.13) | **0.84 (0.71, 0.98)** | 0.84 (0.69, 1.00) | 1.29 (0.81, 3.54) | 1.20 (0.81, 2.81) | 1.13 (0.58, 2.27) | 0.71 (0.21, 1.84) |
|  | Care | 0.87 (0.69, 1.11) | 0.85 (0.62, 1.14) | **0.66 (0.47, 0.88)** | 0.84 (0.61, 1.13) | 0.99 (0.79, 1.25) | 0.89 (0.68, 1.14) | 0.93 (0.52, 1.94) | 1.25 (0.65, 4.92) | 1.14 (0.75, 2.14) | 0.94 (0.40, 2.89) |
|  | Health | 1.02 (0.82, 1.30) | 1.16 (0.85, 1.64) | **1.52 (1.08, 2.26)** | 1.19 (0.86, 1.73) | 1.14 (0.93, 1.45) | 1.04 (0.82, 1.35) | 0.81 (0.52, 1.39) | 0.70 (0.43, 1.18) | 0.95 (0.58, 1.78) | 0.49 (0.12, 1.09) |
| Non-immigrant (n=142) | Thoughts and feelings | 0.88 (0.69, 1.11) | 0.86 (0.66, 1.11) | 0.90 (0.69, 1.19) | 0.82 (0.61, 1.09) | 0.86 (0.68, 1.07) | 0.88 (0.69, 1.11) | 1.01 (0.74, 1.39) | 1.82 (0.95, 5.19) | * | * |
|  | Habits and activities | **0.75 (0.59, 0.92)** | **0.73 (0.58, 0.90)** | **0.62 (0.47, 0.80)** | **0.70 (0.54, 0.89)** | 0.90 (0.74, 1.10) | 0.93 (0.76, 1.14) | 0.82 (0.61, 1.09) | **0.58 (0.36, 0.88)** |  |  |
|  | Social situation | 1.02 (0.79, 1.39) | 1.13 (0.86, 1.60) | 0.92 (0.70, 1.29) | 0.98 (0.74, 1.40) | 0.94 (0.75, 1.20) | 1.05 (0.83, 1.40) | 0.99 (0.73, 1.52) | 0.88 (0.54, 1.77) |  |  |
|  | Economic situation | 1.14 (0.92, 1.47) | 0.93 (0.75, 1.16) | 0.97 (0.77, 1.26) | 1.02 (0.78, 1.34) | 0.96 (0.80, 1.15) | 0.99 (0.82, 1.22) | 1.14 (0.84, 1.64) | 1.20 (0.67, 2.72) |  |  |
|  | Medication | 1.14 (0.86, 1.61) | 1.12 (0.85, 1.55) | **1.79 (1.15, 3.08)** | 1.46 (0.97, 2.43) | 1.03 (0.82, 1.31) | 1.00 (0.79, 1.29) | 1.09 (0.77, 1.70) | 0.81 (0.39, 1.67) |  |  |
|  | Care | 1.02 (0.67, 1.53) | 1.02 (0.68, 1.54) | 1.45 (0.88, 2.49) | 0.74 (0.43, 1.22) | 1.08 (0.74, 1.56) | 1.01 (0.70, 1.45) | 1.31 (0.78, 2.74) | * |  |  |
|  | Health | 0.86 (0.69, 1.09) | 0.94 (0.73, 1.24) | 0.96 (0.74, 1.30) | 1.20 (0.88, 1.79) | 0.83 (0.66, 1.04) | 0.89 (0.70, 1.12) | **0.72 (0.54, 0.93)** | 0.72 (0.49, 1.11) |  |  |

|  | Covariate | **ART adherence:**  **last 30 days** | | **ART adherence:**  **last 7 days** | | **Intention to**  **adhere to ART** | | **Viral load:**  **self-reported** | | **Viral load:**  **plasma** | |
| --- | --- | --- | --- | --- | --- | --- | --- | --- | --- | --- | --- |
|  |  | Time 1 | Time 2 | Time 1 | Time 2 | Time 1 | Time 2 | Time 1 | Time 2 | Time 1 | Time 2 |
| **Age (years)** |  |  |  |  |  |  |  |  |  |  |  |
| < 50 (n=135) | Thoughts and feelings | 0.95 (0.77, 1.17) | 1.02 (0.82, 1.27) | 1.20 (0.92, 1.65) | 0.97 (0.79, 1.21) | 1.03 (0.86, 1.23) | 0.86 (0.70, 1.06) | * | * | 0.80 (0.48, 1.39) | * |
|  | Habits and activities | 0.91 (0.77, 1.08) | **0.68 (0.55, 0.82)** | **0.75 (0.61, 0.92)** | 0.84 (0.70, 1.00) | 0.98 (0.84, 1.15) | 0.95 (0.80, 1.13) |  |  | **0.60 (0.33, 0.94)** |  |
|  | Social situation | 1.02 (0.85, 1.25) | 0.99 (0.80, 1.23) | 0.82 (0.66, 1.01) | 1.02 (0.84, 1.26) | 0.99 (0.85, 1.15) | 1.18 (0.98, 1.45) |  |  | 0.73 (0.33, 1.28) |  |
|  | Economic situation | 1.12 (0.95, 1.35) | 1.02 (0.87, 1.23) | 1.06 (0.88, 1.31) | 0.94 (0.80, 1.11) | **0.86 (0.75, 0.98)** | 0.88 (0.75, 1.02) |  |  | 1.80 (0.91, 5.21) |  |
|  | Medication | 0.89 (0.74, 1.07) | 1.00 (0.80, 1.30) | **0.80 (0.63, 1.00)** | 0.95 (0.77, 1.22) | 0.87 (0.73, 1.03) | 0.89 (0.73, 1.08) |  |  | * |  |
|  | Care | 0.98 (0.71, 1.41) | 0.75 (0.53, 1.02) | **0.67 (0.45, 0.94)** | 0.79 (0.57, 1.06) | 1.15 (0.88, 1.57) | 0.93 (0.70, 1.25) |  |  | 1.40 (0.74, 6.42) |  |
|  | Health | 0.88 (0.71, 1.09) | 1.20 (0.90, 1.69) | 1.37 (0.98, 2.05) | 1.21 (0.90, 1.72) | 0.94 (0.76, 1.16) | 0.92 (0.72, 1.17) |  |  | 0.72 (0.19, 3.47) |  |
| ≥ 50 (n=160) | Thoughts and feelings | **0.69 (0.12, 0.91)** | **0.62 (0.42, 0.86)** | 0.78 (0.57, 1.05) | **0.66 (0.44, 0.94)** | 0.83 (0.66, 1.03) | 0.86 (0.65, 1.12) | 1.22 (0.84, 1.78) | 1.59 (0.78, 4.87) | 1.09 (0.50, 2.49) | * |
|  | Habits and activities | 0.85 (0.67, 1.04) | 1.01 (0.78, 1.31) | **0.76 (0.59, 0.95)** | 0.96 (0.73, 1.30) | 0.87 (0.72, 1.04) | 0.98 (0.78, 1.22) | **0.63 (0.44, 0.86)** | 0.80 (0.52, 1.48) | 0.69 (0.29, 1.37) |  |
|  | Social situation | **1.44 (1.04, 2.12)** | 1.16 (0.83, 1.80) | 1.16 (0.84, 1.72) | 1.05 (0.76, 1.65) | 1.20 (0.95, 1.58) | 1.08 (0.85, 1.45) | 1.05 (0.76, 1.75) | 0.86 (0.53, 1.89) | 1.02 (0.71, 1.79) |  |
|  | Economic situation | 0.92 (0.75, 1.13) | 1.01 (0.81, 1.29) | 1.09 (0.86, 1.42) | 1.16 (0.90, 1.59) | 1.01 (0.86, 1.21) | 1.11 (0.93, 1.36) | 1.31 (0.92, 2.06) | 0.98 (0.63, 2.02) | 1.15 (0.69, 2.14) |  |
|  | Medication | 1.32 (0.97, 1.91) | 1.18 (0.87, 1.67) | 1.33 (0.92, 2.08) | 1.23 (0.85, 1.92) | 0.94 (0.75, 1.17) | 0.96 (0.77, 1.21) | 2.06 (0.99, 7.66) | * | 1.25 (0.79, 2.47) |  |
|  | Care | **0.68 (0.46, 0.94)** | 1.13 (0.77, 1.67) | 0.84 (0.56, 1.22) | 0.77 (0.45, 1.21) | 0.87 (0.64, 1.17) | 0.84 (0.59, 1.17) | 0.92 (0.49, 1.66) | * | 1.16 (0.57, 2.57) |  |
|  | Health | 1.12 (0.86, 1.48) | 0.89 (0.66, 1.20) | 1.00 (0.75, 1.35) | 1.19 (0.85, 1.83) | 1.06 (0.85, 1.34) | 0.98 (0.77, 1.27) | **0.57 (0.39, 0.80)** | 0.70 (0.45, 1.15) | 0.65 (0.30, 1.23) |  |

|  | Covariate | **ART adherence:**  **last 30 days** | | **ART adherence:**  **last 7 days** | | **Intention to**  **adhere to ART** | | **Viral load:**  **self-reported** | | **Viral load:**  **plasma** | |
| --- | --- | --- | --- | --- | --- | --- | --- | --- | --- | --- | --- |
|  |  | Time 1 | Time 2 | Time 1 | Time 2 | Time 1 | Time 2 | Time 1 | Time 2 | Time 1 | Time 2 |
| **Level of education** |  |  |  |  |  |  |  |  |  |  |  |
| Secondary (High school)/ Professional degree (n=109) | Thoughts and feelings | 1.00 (0.78, 1.30) | **0.59 (0.36, 0.86)** | 1.04 (0.78, 1.46) | **0.59 (0.38, 0.87)** | 0.97 (0.79, 1.20) | 0.81 (0.60, 1.06) | 0.98 (0.69, 1.43) | * | * | * |
|  | Habits and activities | **0.73 (0.56, 0.93)** | 0.79 (0.56, 1.07) | 0.84 (0.66, 1.09) | 1.05 (0.77, 1.53) | 0.94 (0.78, 1.14) | 0.98 (0.78, 1.24) | **0.65 (0.43, 0.93)** | 0.72 (0.41, 1.33) | 0.78 (0.36, 1.53) |  |
|  | Social situation | 1.37 (0.96, 2.05) | 0.96 (0.56, 1.70) | 0.89 (0.64, 1.26) | 1.16 (0.74, 1.93) | 1.02 (0.81, 1.27) | 1.17 (0.89, 1.60) | 1.36 (0.67, 2.87) | 1.09 (0.56, 3.10) | 0.54 (0.13, 1.29) |  |
|  | Economic situation | 1.15 (0.91, 1.52) | 1.21 (0.86, 1.91) | 1.04 (0.82, 1.38) | 1.09 (0.83, 1.57) | 0.88 (0.73, 1.04) | 1.05 (0.87, 1.29) | 1.24 (0.85, 2.08) | 0.99 (0.61, 1.86) | * |  |
|  | Medication | 0.97 (0.71, 1.35) | * | 1.19 (0.84, 1.81) | 1.07 (0.70, 1.79) | 1.06 (0.85, 1.33) | 0.93 (0.70, 1.20) | 1.50 (0.79, 4.68) | 0.97 (0.49, 2.82) | 1.12 (0.27, 4.50) |  |
|  | Care | 0.80 (0.54, 1.18) | 0.55 (0.27, 1.04) | 0.83 (0.55, 1.25) | 0.69 (0.40, 1.17) | 0.90 (0.64, 1.24) | 0.76 (0.51, 1.09) | 0.83 (0.39, 1.97) | 0.84 (0.35, 3.30) | 0.72 (0.28, 2.06) |  |
|  | Health | 0.80 (0.62, 1.01) | 0.88 (0.61, 1.36) | 0.87 (0.67, 1.17) | 1.23 (0.83, 2.13) | 0.92 (0.73, 1.14) | 1.16 (0.90, 1.61) | **0.65 (0.44, 0.90)** | 0.75 (0.49, 1.19) | 0.69 (0.30, 2.18) |  |
| College (postsecondary)/ CEGEP/Technical degree (n=59) | Thoughts and feelings | 0.82 (0.55, 1.21) | 1.41 (0.88, 2.59) | 0.92 (0.59, 1.45) | 1.53 (0.91, 3.00) | 0.88 (0.62, 1.25) | 1.12 (0.74, 1.74) | * | * | * | * |
|  | Habits and activities | 0.88 (0.56, 1.35) | 0.56 (0.25, 1.01) | 0.66 (0.38, 1.02) | 0.43 (0.12, 1.12) | 0.88 (0.58, 1.26) | 0.78 (0.47, 1.22) |  |  |  |  |
|  | Social situation | 1.44 (0.94, 2.64) | 1.02 (0.54, 2.37) | 1.26 (0.79, 2.39) | 1.58 (0.59, 5.12) | 1.21 (0.84, 2.03) | 1.53 (0.95, 3.11) |  |  |  |  |
|  | Economic situation | 1.05 (0.83, 1.41) | 0.96 (0.67, 1.37) | 0.98 (0.75, 1.35) | 0.85 (0.57, 1.24) | 1.12 (0.90, 1.47) | 0.86 (0.64, 1.12) |  |  |  |  |
|  | Medication | 0.77 (0.50, 1.07) | 0.79 (0.47, 1.14) | 0.62 (0.33, 1.01) | 0.79 (0.44, 1.18) | 0.77 (0.49, 1.06) | 0.62 (0.30, 1.14) |  |  |  |  |
|  | Care | 1.10 (0.67, 1.82) | 1.84 (0.84, 5.24) | 0.77 (0.35, 1.51) | 0.79 (0.26, 2.21) | 0.79 (0.48, 1.24) | 1.15 (0.62, 2.17) |  |  |  |  |
|  | Health | 0.92 (0.64, 1.29) | 1.09 (0.70, 1.97) | **2.33 (1.22, 5.52)** | * | 1.18 (0.88, 1.68) | 0.82 (0.52, 1.22) |  |  |  |  |
|  |  |  |  |  |  |  |  |  |  |  |  |
| University (n=104) | Thoughts and feelings | 0.89 (0.61, 1.28) | 0.98 (0.68, 1.42) | 1.13 (0.70, 1.86) | 0.78 (0.49, 1.19) | 1.13 (0.84, 1.56) | 0.85 (0.61, 1.17) | * | * | * | * |
|  | Habits and activities | 0.80 (0.60, 1.04) | **0.67 (0.50, 0.87)** | **0.64 (0.43, 0.91)** | **0.69 (0.51, 0.93)** | 0.92 (0.72, 1.17) | 1.06 (0.83, 1.40) |  |  |  |  |
|  | Social situation | 1.07 (0.81, 1.55) | 1.05 (0.79, 1.50) | 0.91 (0.64, 1.45) | 1.14 (0.78, 1.93) | 0.99 (0.80, 1.29) | 1.22 (0.92, 1.73) |  |  |  |  |
|  | Economic situation | 0.83 (0.64, 1.06) | 0.86 (0.68, 1.11) | 0.89 (0.64, 1.26) | 0.77 (0.55, 1.06) | **0.67 (0.50, 0.84)** | 0.90 (0.72, 1.13) |  |  |  |  |
|  | Medication | 1.02 (0.73, 1.53) | 0.96 (0.71, 1.36) | 1.09 (0.65, 2.16) | 2.00 (0.96, 4.81) | 1.03 (0.78, 1.38) | 0.83 (0.63, 1.09) |  |  |  |  |
|  | Care | 0.72 (0.41, 1.11) | 0.81 (0.53, 1.20) | 0.66 (0.36, 1.08) | 0.72 (0.38, 1.11) | **1.97 (1.17, 3.76)** | 1.10 (0.75, 1.82) |  |  |  |  |
|  | Health | 1.62 (1.00, 2.82) | 1.26 (0.84, 1.97) | 1.21 (0.73, 2.15) | 1.22 (0.74, 2.11) | 0.65 (0.41, 1.00) | 0.80 (0.54, 1.16) |  |  |  |  |

|  | Covariate | **ART adherence:**  **last 30 days** | | **ART adherence:**  **last 7 days** | | **Intention to**  **adhere to ART** | | **Viral load:**  **self-reported** | | **Viral load:**  **plasma** | |
| --- | --- | --- | --- | --- | --- | --- | --- | --- | --- | --- | --- |
|  |  | Time 1 | Time 2 | Time 1 | Time 2 | Time 1 | Time 2 | Time 1 | Time 2 | Time 1 | Time 2 |
| **Sex** |  |  |  |  |  |  |  |  |  |  |  |
| Female (n=94) | Thoughts and feelings | 0.86 (0.63, 1.18) | 0.58 (0.29, 1.04) | 1.10 (0.82, 1.58) | 0.80 (0.53, 1.17) | 0.94 (0.74, 1.19) | 1.07 (0.70, 1.62) | **2.12 (1.03, 4.37)** | * | * | * |
|  | Habits and activities | 0.85 (0.68, 1.04) | 0.70 (0.47, 1.00) | **0.79 (0.61, 0.98)** | 0.89 (0.69, 1.16) | 0.93 (0.77, 1.11) | 0.80 (0.59, 1.03) | **0.57 (0.36, 0.79)** |  |  |  |
|  | Social situation | 1.02 (0.80, 1.36) | 1.31 (0.79, 2.53) | 0.83 (0.66, 1.07) | 1.01 (0.71, 1.52) | 1.04 (0.86, 1.27) | 1.39 (0.99, 2.17) | 0.76 (0.44, 1.30) |  |  |  |
|  | Economic situation | 1.18 (0.95, 1.53) | 1.22 (0.90, 1.83) | 1.04 (0.83, 1.35) | 1.12 (0.88, 1.49) | 0.94 (0.80, 1.10) | 1.01 (0.82, 1.27) | 1.32 (0.90, 2.09) |  |  |  |
|  | Medication | 1.02 (0.80, 1.33) | 1.41 (0.91, 2.38) | 0.99 (0.75, 1.35) | 1.10 (0.78, 1.64) | 0.95 (0.78, 1.15) | 0.78 (0.56, 1.03) | 1.28 (0.75, 2.46) |  |  |  |
|  | Care | 0.85 (0.63, 1.12) | 0.87 (0.58, 1.26) | 0.78 (0.53, 1.07) | 0.92 (0.64, 1.32) | 1.10 (0.85, 1.46) | 0.72 (0.47, 1.03) | 0.89 (0.47, 1.64) |  |  |  |
|  | Health | 1.01 (0.79, 1.33) | 1.13 (0.78, 1.71) | 1.35 (0.94, 2.13) | 1.19 (0.86, 1.78) | 0.97 (0.77, 1.22) | 1.14 (0.86, 1.58) | **0.61 (0.36, 0.95)** |  |  |  |
| Male (n=207) | Thoughts and feelings | 0.92 (0.76, 1.10) | 0.98 (0.80, 1.20) | 0.93 (0.75, 1.18) | 0.90 (0.73, 1.13) | 0.96 (0.82, 1.13) | **0.83 (0.69, 0.98)** | 0.80 (0.59, 1.08) | 1.19 (0.62, 3.69) | 0.87 (0.59, 1.38) | 2.50 (1.12, 7.69) |
|  | Habits and activities | 0.87 (0.74, 1.02) | **0.76 (0.64, 0.91)** | **0.77 (0.64, 0.92)** | 0.86 (0.71, 1.04) | 0.95 (0.82, 1.10) | 1.04 (0.88, 1.25) | 0.87 (0.68, 1.19) | 1.13 (0.68, 3.35) | **0.61 (0.37, 0.89)** | 0.63 (0.31, 1.30) |
|  | Social situation | 1.22 (1.00, 1.53) | 1.08 (0.88, 1.35) | 1.10 (0.87, 1.43) | 1.08 (0.87, 1.39) | 0.99 (0.85, 1.16) | 1.10 (0.91, 1.35) | 1.60 (0.96, 3.65) | 1.18 (0.64, 3.59) | 1.03 (0.74, 1.60) | 1.06 (0.69, 2.13) |
|  | Economic situation | 0.90 (0.78, 1.05) | 0.90 (0.76, 1.08) | 1.01 (0.84, 1.25) | 0.94 (0.79, 1.14) | 0.89 (0.78, 1.01) | 0.93 (0.80, 1.08) | 1.19 (0.87, 2.17) | 1.06 (0.66, 2.53) | 1.43 (0.83, 3.33) | 0.84 (0.44, 1.76) |
|  | Medication | 0.97 (0.81, 1.18) | 0.97 (0.80, 1.21) | 0.97 (0.78, 1.26) | 0.96 (0.77, 1.25) | 0.91 (0.77, 1.08) | 0.91 (0.76, 1.09) | 1.18 (0.83, 2.43) | 1.03 (0.72, 2.30) | 1.87 (0.99, 4.62) | 1.49 (0.82, 3.41) |
|  | Care | 1.15 (0.84, 1.63) | 1.12 (0.78, 1.66) | 0.90 (0.61, 1.36) | 0.79 (0.54, 1.16) | 1.06 (0.80, 1.40) | 1.03 (0.76, 1.42) | * | 0.91 (0.39, 2.83) | 1.44 (0.68, 3.85) | 1.82 (0.5, 8.28) |
|  | Health | 0.84 (0.68, 1.04) | 0.91 (0.71, 1.20) | 0.94 (0.73, 1.28) | 1.20 (0.87, 1.74) | 1.00 (0.82, 1.24) | 0.88 (0.70, 1.10) | 0.75 (0.54, 1.01) | 0.77 (0.55, 1.19) | 0.43 (0.16, 1.06) | **0.16 (0.03, 0.54)** |

|  | Covariate | **ART adherence:**  **last 30 days** | | **ART adherence:**  **last 7 days** | | **Intention to**  **adhere to ART** | | **Viral load:**  **self-reported** | | **Viral load:**  **plasma** | |
| --- | --- | --- | --- | --- | --- | --- | --- | --- | --- | --- | --- |
|  |  | Time 1 | Time 2 | Time 1 | Time 2 | Time 1 | Time 2 | Time 1 | Time 2 | Time 1 | Time 2 |
| **Sexual orientation** |  |  |  |  |  |  |  |  |  |  |  |
| Heterosexual (n=119) | Thoughts and feelings | 1.00 (0.75, 1.36) | **0.53 (0.30, 0.85)** | 1.26 (0.89, 1.89) | 0.62 (0.38, 1.00) | 1.03 (0.78, 1.38) | 0.97 (0.67, 1.39) | 1.03 (0.61, 1.78) | * | * | * |
|  | Habits and activities | **0.75 (0.58, 0.92)** | 0.91 (0.63, 1.27) | **0.70 (0.53, 0.89)** | 0.99 (0.73, 1.36) | 0.83 (0.65, 1.01) | 0.83 (0.60, 1.09) | **0.62 (0.42, 0.85)** |  |  |  |
|  | Social situation | 0.86 (0.63, 1.15) | 1.35 (0.83, 2.39) | 0.68 (0.45, 1.01) | 1.24 (0.80, 2.07) | 1.03 (0.75, 1.39) | 1.13 (0.80, 1.64) | 0.90 (0.49, 1.70) |  |  |  |
|  | Economic situation | 1.01 (0.85, 1.21) | 1.29 (0.97, 1.85) | 1.15 (0.90, 1.58) | 1.14 (0.89, 1.56) | **0.78 (0.64, 0.94)** | 0.98 (0.81, 1.18) | 1.50 (0.97, 2.99) |  |  |  |
|  | Medication | 0.91 (0.70, 1.18) | 1.36 (0.86, 2.50) | 1.00 (0.73, 1.42) | 1.12 (0.77, 1.81) | 0.81 (0.61, 1.04) | 0.78 (0.57, 1.04) | * |  |  |  |
|  | Care | 0.87 (0.60, 1.22) | 0.70 (0.41, 1.10) | 0.73 (0.45, 1.08) | 0.79 (0.50, 1.20) | 1.21 (0.87, 1.78) | 0.95 (0.67, 1.37) | 0.80 (0.45, 1.52) |  |  |  |
|  | Health | 1.19 (0.85, 1.74) | 0.93 (0.59, 1.51) | 1.19 (0.79, 1.92) | 1.06 (0.70, 1.67) | 1.39 (0.98, 2.04) | 1.32 (0.93, 1.96) | **0.52 (0.27, 0.89)** |  |  |  |
| Homosexual (n=137) | Thoughts and feelings | 0.93 (0.73, 1.18) | 1.20 (0.91, 1.62) | 0.78 (0.58, 1.05) | 1.08 (0.82, 1.45) | 0.90 (0.74, 1.10) | 0.91 (0.72, 1.14) | 0.85 (0.57, 1.24) | 1.25 (0.65, 3.87) | 0.94 (0.59, 1.65) | * |
|  | Habits and activities | 0.84 (0.67, 1.04) | **0.70 (0.55, 0.87)** | **0.77 (0.59, 0.98)** | 0.82 (0.65, 1.05) | 0.90 (0.74, 1.08) | 1.02 (0.83, 1.29) | 1.09 (0.73, 2.14) | 1.20 (0.69, 3.82) | 0.64 (0.32, 1.04) |  |
|  | Social situation | 1.12 (0.87, 1.50) | 1.07 (0.84, 1.41) | 1.19 (0.87, 1.72) | 1.03 (0.80, 1.38) | 0.98 (0.80, 1.21) | 1.05 (0.85, 1.34) | 1.40 (0.83, 3.59) | 1.13 (0.63, 3.32) | 0.90 (0.61, 1.48) |  |
|  | Economic situation | 0.94 (0.78, 1.15) | **0.78 (0.60, 0.97)** | 1.01 (0.80, 1.35) | 0.82 (0.65, 1.02) | 0.91 (0.77, 1.07) | 0.87 (0.72, 1.05) | 1.12 (0.80, 2.17) | 0.99 (0.59, 2.32) | 1.25 (0.70, 2.84) |  |
|  | Medication | 1.08 (0.86, 1.41) | 1.09 (0.84, 1.48) | 1.32 (0.92, 2.21) | 1.13 (0.83, 1.66) | 0.99 (0.81, 1.21) | 1.03 (0.83, 1.31) | 1.10 (0.77, 2.16) | 1.01 (0.68, 2.19) | * |  |
|  | Care | 1.25 (0.80, 2.10) | 1.20 (0.76, 2.00) | 0.91 (0.49, 1.79) | 0.76 (0.47, 1.20) | 0.97 (0.67, 1.40) | 0.98 (0.68, 1.43) | * | 0.93 (0.40, 2.88) | * |  |
|  | Health | **0.73 (0.55, 0.93)** | 0.96 (0.72, 1.37) | 1.03 (0.75, 1.58) | 1.15 (0.81, 1.84) | 0.87 (0.69, 1.08) | 0.83 (0.64, 1.06) | 0.77 (0.56, 1.04) | 0.82 (0.58, 1.28) | 0.33 (0.07, 1.01) |  |

|  | Covariate | **ART adherence:**  **last 30 days** | | **ART adherence:**  **last 7 days** | | **Intention to**  **adhere to ART** | | **Viral load:**  **self-reported** | | **Viral load:**  **plasma** | |
| --- | --- | --- | --- | --- | --- | --- | --- | --- | --- | --- | --- |
|  |  | Time 1 | Time 2 | Time 1 | Time 2 | Time 1 | Time 2 | Time 1 | Time 2 | Time 1 | Time 2 |
| **Administration mode** |  |  |  |  |  |  |  |  |  |  |  |
| Face-to-face interview (n=69) | Thoughts and feelings | 0.88 (0.53, 1.29) | 0.41 (0.11, 1.14) | 1.16 (0.82, 1.78) | 0.59 (0.28, 1.07) | 0.93 (0.62, 1.30) | 0.35 (0.09, 1.01) | ***** | 1.84 (0.94, 5.50) | * | * |
|  | Habits and activities | **0.52 (0.32, 0.75)** | 1.26 (0.82, 2.41) | **0.62 (0.39, 0.86)** | 1.22 (0.84, 2.14) | 0.84 (0.61, 1.10) | 0.66 (0.23, 1.20) |  | 0.83 (0.54, 1.36) |  |  |
|  | Social situation | 1.02 (0.60, 1.77) | 0.61 (0.22, 1.49) | 0.61 (0.33, 1.10) | 1.09 (0.60, 2.20) | 0.87 (0.55, 1.27) | 0.72 (0.26, 1.52) |  | 0.61 (0.24, 1.36) |  |  |
|  | Economic situation | 1.15 (0.84, 1.69) | 1.20 (0.68, 2.53) | 1.33 (0.90, 2.24) | 1.31 (0.90, 2.10) | **0.68 (0.48, 0.89)** | 1.05 (0.73, 1.52) |  | 0.92 (0.57, 1.57) |  |  |
|  | Medication | 0.91 (0.57, 1.49) | * | * | 0.98 (0.68, 1.49) | 1.13 (0.78, 1.66) | 1.00 (0.57, 1.80) |  | 1.65 (0.84, 5.25) |  |  |
|  | Care | **2.37 (1.16, 6.34)** | 0.88 (0.23, 3.38) | * | 1.33 (0.53, 5.46) | 1.68 (1.00, 2.96) | 1.13 (0.55, 2.55) |  | 1.20 (0.47, 5.35) |  |  |
|  | Health | 0.98 (0.64, 1.67) | 0.86 (0.48, 1.55) | 0.72 (0.47, 1.91) | 0.86 (0.49, 1.57) | 1.04 (0.71, 1.58) | 1.75 (0.86, 7.35) |  | 1.05 (0.55, 3.55) |  |  |
| Self-administration online (n=184) | Thoughts and feelings | 0.84 (0.68, 1.03) | 0.99 (0.80, 1.23) | 0.93 (0.72, 1.22) | 0.97 (0.76, 1.24) | 0.89 (0.74, 1.07) | 0.85 (0.69, 1.04) | 1.06 (0.73, 1.64) | 1.96 (0.92, 5.62) | 0.84 (0.54, 1.41) | * |
|  | Habits and activities | 0.96 (0.80, 1.14) | **0.76 (0.63, 0.89)** | 0.82 (0.67, 1.01) | **0.78 (0.64, 0.94)** | 1.00 (0.85, 1.19) | 1.01 (0.86, 1.20) | 0.82 (0.61, 1.13) | 0.79 (0.53, 1.23) | **0.59 (0.33, 0.92)** | **0.50 (0.23, 0.97)** |
|  | Social situation | 1.11 (0.92, 1.38) | 1.05 (0.86, 1.30) | 1.02 (0.82, 1.31) | 0.99 (0.80, 1.27) | 0.94 (0.80, 1.10) | 1.03 (0.85, 1.26) | 1.16 (0.80, 1.84) | 1.15 (0.53, 3.15) | 0.99 (0.70, 1.63) | 1.18 (0.67, 2.61) |
|  | Economic situation | 1.01 (0.87, 1.19) | 0.93 (0.78, 1.11) | 1.00 (0.84, 1.23) | 0.92 (0.76, 1.12) | 1.00 (0.87, 1.15) | 1.07 (0.91, 1.28) | 1.05 (0.80, 1.51) | 0.95 (0.47, 1.94) | 1.35 (0.82, 2.95) | 0.82 (0.42, 1.70) |
|  | Medication | 0.94 (0.80, 1.12) | 0.96 (0.79, 1.19) | 0.86 (0.71, 1.06) | 1.01 (0.80, 1.32) | 0.89 (0.76, 1.04) | 0.89 (0.74, 1.08) | 1.01 (0.78, 1.42) | 0.85 (0.58, 1.39) | 1.47 (0.75, 3.66) | 1.46 (0.77, 3.42) |
|  | Care | 0.85 (0.64, 1.13) | 0.86 (0.66, 1.13) | 0.74 (0.53, 1.02) | 0.73 (0.53, 1.00) | 0.87 (0.66, 1.13) | 0.95 (0.74, 1.23) | 1.35 (0.78, 2.77) | * | 1.02 (0.48, 2.49) | 1.04 (0.37, 3.40) |
|  | Health | 0.93 (0.76, 1.15) | 1.03 (0.80, 1.33) | 1.11 (0.85, 1.51) | 1.35 (1.00, 1.89) | 0.98 (0.80, 1.21) | 0.83 (0.66, 1.04) | **0.69 (0.51, 0.92)** | **0.41 (0.22, 0.68)** | 0.80 (0.39, 2.00) | **0.15 (0.02, 0.49)** |

*Note*. *Inconclusive estimate, Green cell = statistically significant result.
