# Supplementary material for: A multi-phase project to develop a patient-reported measure of barriers to antiretroviral therapy adherence for use in HIV care: The 7-Item I-Score
Source: PLoS One. 2026 Jan 6;21(1):e0324241. doi: 10.1371/journal.pone.0324241 (PMC12774347; doi:10.1371/journal.pone.0324241)
Supplement: Attachment — Submitted filename: PONE-D-24-21594_reviewer and author response.pdf [file pone.0324241.s009.pdf]

PLOS ONE

A multi-phase project to develop a patient-reported measure of barriers to antiretroviral therapy adherence for use in HIV care: The 7-Item I-Score

--Manuscript Draft--

**Manuscript Number:**	PONE-D-24-21594
**Article Type:**	Research Article
**Full Title:**	A multi-phase project to develop a patient-reported measure of barriers to antiretroviral therapy adherence for use in HIV care: The 7-Item I-Score
**Short Title:**	Development of a 7-item patient-reported measure of adherence barriers for HIV care (the I-Score)
**Corresponding Author:**	Kim Engler, Ph.D.<br>Research Institute of the McGill University Health Centre<br>Montreal, Quebec CANADA
**Keywords:**	HIV; antiretroviral therapy; Adherence; Patient-Reported Outcome Measure; measurement properties; validity; cross-cultural validity; reliability; measurement error; Conceptual Framework; cognitive testing; HIV care; Canada
**Abstract:**	Introduction While adherence to antiretroviral therapy (ART) is a priority in HIV care, there is no consensus on how to assess and manage adherence and its problems in this context.<br>Objectives Our goal was to develop and validate a new, short patient-reported outcome measure (PROM) of barriers to daily oral ART adherence specifically for use in HIV care in Canada and France.<br>Methods The overarching design was that of a multi-phase, multi-site instrument development project, involving a qualitative research synthesis (to develop a conceptual framework from which to generate items), cognitive interviews (for content validity testing of the preliminary items), and a longitudinal survey-based study where the revised 7-item instrument (the I-Score) was administered at two timepoints (baseline, 4 weeks), along with five indirect measures of ART adherence, including self-reported HIV viral load and, for a subset of Montreal participants, tested viral load. The survey data was used to analyze the instrument's measurement properties, namely structural validity (e.g., with Pearson inter-item correlations); construct validity, including cross-cultural validity, for a variety of sociodemographic groups (with receiver operating curve (ROC) analyses and areas under the curve (AUC); reliability (with the intraclass correlation coefficient (ICC)); and measurement error (with the standard error of measurement (SEM). Study participants were adults with HIV on antiretroviral therapy recruited from hospital-based infectious disease centers and community-based organizations in Montreal, Toronto, and Paris.<br>Results The qualitative synthesis of 41 studies led to a framework composed of 6 barrier domains. One item was generated per domain. The 6-item measure was submitted to cognitive testing with 12 adults with HIV, leading to several changes, including the division of one item into two, creating a 7-item measure. Overall, 305 adults with HIV participated in the survey. Inter-item correlations were low to moderate, suggesting no redundant items. Among conclusive results, AUC's were all above the predefined threshold (≥ 0.70) for self-reported viral load and plasma viral load for the global sample and across all subgroups examined. The ICC, at 0.81, was also above the predefined threshold (³ 0.70). The SEM was 0.43.<br>Conclusion The evidence generated supports the 7-item I-Score PROM's content validity, construct validity/cross-cultural validity, reliability, and acceptable measurement error, in its specified context of use.
**Order of Authors:**	Kim Engler, PhD
	David Lessard, PhD
	Serge Vicente, PhD
	Darrell HS Tan, MD, PhD
	Karine Lacombe, MD, PhD

Powered by Editorial Manager® and ProduXion Manager® from Aries Systems Corporation

	Bertrand Lebouché, MD, PhD
**Additional Information:**	
**Question**	**Response**
**Financial Disclosure**<br><br>Enter a financial disclosure statement that describes the sources of funding for the work included in this submission. Review the submission guidelines for detailed requirements. View published research articles from *PLOS ONE* for specific examples.<br><br>This statement is required for submission and **will appear in the published article** if the submission is accepted. Please make sure it is accurate.<br><br>**Funded studies**<br>Enter a statement with the following details:<br>• Initials of the authors who received each award<br>• Grant numbers awarded to each author<br>• The full name of each funder<br>• URL of each funder website<br>• Did the sponsors or funders play any role in the study design, data collection and analysis, decision to publish, or preparation of the manuscript?<br><br>Did you receive funding for this work?	Yes
Please add funding details.<br>as follow-up to "**Financial Disclosure**<br><br>Enter a financial disclosure statement that describes the sources of funding for the work included in this submission. Review the submission guidelines for detailed requirements. View published research articles from *PLOS ONE* for specific examples.<br><br>This statement is required for submission and **will appear in the published article** if the submission is accepted. Please make sure it is accurate.	This work received support from the Canadian Institutes of Health Research (CIHR) HIV Clinical Trials Network (CTN) (CTN283). It was conducted within the program of a SPOR Mentorship Chair in Innovative Clinical Trials awarded by the CIHR to BL (Grant No. 383427). It benefited from methodological expertise and funding from the Quebec SPOR Support Unit -McGill Methodological Developments Platform (Grant number M006). It was also supported, in part, by a research grant from the Investigator Initiated Studies Program of Merck Canada Inc. (Grant number IISP-53538), which had no hand in the design, conduct, or writing up of this work. French (France) participation was supported, in part, by funding from MSD Avenir awarded to KL and managed by the AP-HP Foundation (MSDAVENIR DS-2018-0072).

**Funded studies**<br>Enter a statement with the following details:<br>• Initials of the authors who received each award<br>• Grant numbers awarded to each author<br>• The full name of each funder<br>• URL of each funder website<br>• Did the sponsors or funders play any role in the study design, data collection and analysis, decision to publish, or preparation of the manuscript?<br><br>Did you receive funding for this work?"	
Please select the country of your main research funder (please select carefully as in some cases this is used in fee calculation).<br>as follow-up to "**Financial Disclosure**<br><br>Enter a financial disclosure statement that describes the sources of funding for the work included in this submission. Review the submission guidelines for detailed requirements. View published research articles from *PLOS ONE* for specific examples.<br><br>This statement is required for submission and **will appear in the published article** if the submission is accepted. Please make sure it is accurate.<br><br>**Funded studies**<br>Enter a statement with the following details:<br>• Initials of the authors who received each award<br>• Grant numbers awarded to each author<br>• The full name of each funder<br>• URL of each funder website<br>• Did the sponsors or funders play any role in the study design, data collection and analysis, decision to publish, or preparation of the manuscript?<br><br>Did you receive funding for this work?"	CANADA - CA
**Competing Interests**	BL has received research support and consulting fees from ViiV Healthcare, Merck,

Use the instructions below to enter a competing interest statement for this submission. On behalf of all authors, disclose any competing interests that could be perceived to bias this work—acknowledging all financial support and any other relevant financial or non-financial competing interests.<br><br>This statement is required for submission and **will appear in the published article** if the submission is accepted. Please make sure it is accurate and that any funding sources listed in your Funding Information later in the submission form are also declared in your Financial Disclosure statement.<br><br>View published research articles from *PLOS ONE* for specific examples.<br><br>**NO authors have competing interests**<br><br>Enter: *The authors have declared that no competing interests exist.*<br><br>**Authors with competing interests**<br><br>Enter competing interest details beginning with this statement:<br><br>*I have read the journal's policy and the authors of this manuscript have the following competing interests: [insert competing interests here]*<br><br>* typeset	and Gilead. KL has received travel support and consulting fees from ViiV Healthcare, Merck, and Gilead. DHST's institution has received research support for investigator-initiated research studies from Abbvie and Gilead, and for participation in industry-sponsored trials from Glaxo Smith Kline. The other authors have no competing interests to declare.
**Ethics Statement**<br><br>Enter an ethics statement for this submission. This statement is required if the study involved:<br><br>• Human participants<br>• Human specimens or tissue<br>• Vertebrate animals or cephalopods<br>• Vertebrate embryos or tissues	The I-Score Study obtained ethics approval from the Research Ethics Board (REB) of the McGill University Health Centre (MUHC) in Montreal, Quebec, Canada (original approval received December 1, 2014: 2015-2331, 14-229 PSY). In addition to annual renewals until November 29, 2023, two subsequent amendments were also approved by the MUHC REB (an amendment to the I-Score measure's validation plan: approved January 18, 2022; an amendment for the retrospective analysis of viral load test results of patients with HIV at the Chronic Viral Illness Service of the McGill University Health Centre, waiving the requirement for informed consent: approved September 21, 2023). For the Toronto site (St. Michael's Hospital), REB approval was originally obtained from Unity Health Toronto, Toronto, Ontario, Canada, on November 10, 2016 (REB#

Powered by Editorial Manager® and ProduXion Manager® from Aries Systems Corporation

• Field research<br><br>Write "N/A" if the submission does not require an ethics statement.<br><br>General guidance is provided below. Consult the submission guidelines for detailed instructions. **Make sure that all information entered here is included in the Methods section of the manuscript.**	16-104) and renewed until November 10, 2023. For the affiliated French sites, approval was obtained from the REB of the Nantes University Health Centre in Nantes, France, on March 6, 2018 (RC17_0310 N° EUDRACT: 2017-A02460-53; French legislation requires no subsequent annual renewals). The project to meaningfully involve people with HIV in the I-Score Study, in part through a patient advisory committee, was approved by the MUHC REB on September 8, 2015 (project number: 15-188-MUHC).

Powered by Editorial Manager® and ProduXion Manager® from Aries Systems Corporation

**Format for specific study types**<br><br>**Human Subject Research (involving human participants and/or tissue)**<br>• Give the name of the institutional review board or ethics committee that approved the study<br>• Include the approval number and/or a statement indicating approval of this research<br>• Indicate the form of consent obtained (written/oral) or the reason that consent was not obtained (e.g. the data were analyzed anonymously)<br><br>**Animal Research (involving vertebrate animals, embryos or tissues)**<br>• Provide the name of the Institutional Animal Care and Use Committee (IACUC) or other relevant ethics board that reviewed the study protocol, and indicate whether they approved this research or granted a formal waiver of ethical approval<br>• Include an approval number if one was obtained<br>• If the study involved *non-human primates*, add *additional details* about animal welfare and steps taken to ameliorate suffering<br>• If anesthesia, euthanasia, or any kind of animal sacrifice is part of the study, include briefly which substances and/or methods were applied<br><br>**Field Research**<br><br>Include the following details if this study involves the collection of plant, animal, or other materials from a natural setting:<br>• Field permit number<br>• Name of the institution or relevant body that granted permission	
**Data Availability**<br><br>Authors are required to make all data underlying the findings described fully available, without restriction, and from the time of publication. PLOS allows rare exceptions to address legal and ethical concerns. See the PLOS Data Policy and FAQ for detailed information.	Yes - all data are fully available without restriction

A Data Availability Statement describing where the data can be found is required at submission. Your answers to this question constitute the Data Availability Statement and **will be published in the article**, if accepted.<br><br>**Important:** Stating 'data available on request from the author' is not sufficient. If your data are only available upon request, select 'No' for the first question and explain your exceptional situation in the text box.<br><br>Do the authors confirm that all data underlying the findings described in their manuscript are fully available without restriction?	
**Describe where the data may be found in full sentences. If you are copying our sample text, replace any instances of XXX with the appropriate details.**<br><br>• If the data are **held or will be held in a public repository**, include URLs, accession numbers or DOIs. If this information will only be available after acceptance, indicate this by ticking the box below. For example: *All XXX files are available from the XXX database (accession number(s) XXX, XXX.).*<br>• If the data are all contained **within the manuscript and/or Supporting Information files**, enter the following: *All relevant data are within the manuscript and its Supporting Information files.*<br>• If neither of these applies but you are able to provide **details of access elsewhere**, with or without limitations, please do so. For example:<br><br>*Data cannot be shared publicly because of [XXX]. Data are available from the XXX Institutional Data Access / Ethics Committee (contact via XXX) for researchers who meet the criteria for access to confidential data.*<br><br>*The data underlying the results presented in the study are available from (include the name of the third party*	The data underlying the results presented in the study are available from [will be specified upon acceptance].

and contact information or URL).<br>• This text is appropriate if the data are owned by a third party and authors do not have permission to share the data.<br><br>* typeset	
Additional data availability information:	

Manuscript
Click here to access/download;Manuscript;MANUSCRIPT_The 7 item I-Score July 15 2024.docx

A multi-phase project to develop a patient-reported measure of barriers to antiretroviral therapy adherence for use in HIV care: The 7-Item I-Score

Kim Engler[1]*, David Lessard[1¶], Serge Vicente[2¶], Darrell H. S. Tan[3,4,5], Karine Lacombe[6], Bertrand Lebouché[1,7]

[1] Center for Outcomes Research and Evaluation, Research Institute of the McGill University Health Centre, Montreal, Quebec, Canada

[2] Department of Mathematics and Statistics, University of Montreal, Montreal, Quebec, Canada

[3] Division of Infectious Diseases, St. Michael's Hospital, Toronto, Canada

[4] Department of Medicine, University of Toronto, Toronto, Canada

[5] Institute of Health Policy, Management and Evaluation, University of Toronto, Toronto, Canada

[6] Service des maladies infectieuses et tropicales, Hôpital Saint-Antoine, Assistance Publique-Hôpitaux de Paris, Paris, France

[7] Department of Family Medicine, McGill University, Montreal, Quebec, Canada

*Corresponding author.

E-mail: kimcengler@gmail.com

[¶]These authors contributed equally to this work.

Abstract

Introduction While adherence to antiretroviral therapy (ART) is a priority in HIV care, there is no consensus on how to assess and manage adherence and its problems in this context.

Objectives Our goal was to develop and validate a new, short patient-reported outcome measure (PROM) of barriers to daily oral ART adherence specifically for use in HIV care in Canada and France.

Methods The overarching design was that of a multi-phase, multi-site instrument development project, involving a qualitative research synthesis (to develop a conceptual framework from which to generate items), cognitive interviews (for content validity testing of the preliminary items), and a longitudinal survey-based study where the revised 7-item instrument (the I-Score) was administered at two timepoints (baseline, 4 weeks), along with five indirect measures of ART adherence, including self-reported HIV viral load and, for a subset of Montreal participants, tested viral load. The survey data was used to analyze the instrument's measurement properties, namely structural validity (e.g., with Pearson inter-item correlations); construct validity, including cross-cultural validity, for a variety of sociodemographic groups (with receiver operating curve (ROC) analyses and areas under the curve (AUC); reliability (with the intraclass correlation coefficient (ICC)); and measurement error (with the standard error of measurement (SEM). Study participants were adults with HIV on antiretroviral therapy recruited from hospital-based infectious disease centers and community-based organizations in Montreal, Toronto, and Paris.

Results The qualitative synthesis of 41 studies led to a framework composed of 6 barrier domains. One item was generated per domain. The 6-item measure was submitted to cognitive testing with 12 adults with HIV, leading to several changes, including the division of one item into two, creating a 7-item measure. Overall, 305 adults with HIV participated in the survey. Inter-item correlations were low to moderate, suggesting no redundant items. Among conclusive results, AUC's were all above the predefined threshold (≥ 0.70) for self-reported viral load and plasma viral load for the global sample and across all subgroups examined. The ICC, at 0.81, was also above the predefined threshold (≥ 0.70). The SEM was 0.43.

Conclusion The evidence generated supports the 7-item I-Score PROM's content validity, construct validity/cross-cultural validity, reliability, and acceptable measurement error, in its specified context of use.

Data availability statement

Data are available upon reasonable request.

Strengths and limitations of this study

- This project employed a multi-phase design to generate a range of qualitative and quantitative evidence in support of a new patient-reported outcome measure.
- It produced evidence of cross-cultural validity and the standard error of measurement, which are uncommonly reported measurement properties of adherence-related patient-report instruments.

- The small survey sample size of certain socioeconomic subgroups contributed to some inconclusive results in the construct validity analyses (areas under the curve).
- Establishing the generalizability of the results to all people with HIV in each country (Canada and France) requires further investigation.

Introduction

The prevention and management of HIV infection continues to be a pressing concern worldwide. In Canada, the Public Health Agency estimated the number of people living with HIV (PLHIV) in 2020 to be over 62,000[1] and recorded a 25% increase in new diagnoses, from 2021 to 2022.[2] In Montreal (Quebec), the second most populous city in the country, new HIV diagnoses more than doubled over the same period.[3]

Among PLHIV, antiretroviral therapy (ART) is crucial to control the infection and its onward transmission. However, PLHIV report a wide range of impediments to taking their medication[4] and almost half of adults with HIV in Canada (45%) are estimated to have suboptimal ART adherence,[5] comparable to global estimates.[6,7] Healthcare providers are not always accurate judges of patient adherence[8] and HIV viral load is often used as a proxy in clinical practice.[9] Importantly, most PLHIV on ART in Canada (95%) are estimated to have achieved the treatment goal of viral suppression (i.e. with a last HIV RNA test result of <200 copies/ml),[1] similar to global estimates.[10] Nevertheless, concerns remain not only for the residual 5%, but for sub-population differences in the attainment of viral suppression, with lower levels observed among females, individuals who inject drugs, and Indigenous peoples in Canada.[1] Additional concerns include attrition over time among the virally suppressed;[11] unplanned treatment interruptions[12] or cessation;[13] and the role of adherence in persistent low-level viremia (e.g., consecutive viral loads ≥50 copies/ml) and the latter's clinical implications, such as ART resistance.[14, 15]

Adherence is a recognized priority for HIV service delivery.[16] However, there is no consensus and limited guidance on the most reliable way to identify adherence challenges, including among patients engaged in care models involving less interaction with the healthcare system.[16] Overall, these observations highlight the complexity of adherence, potential gaps in clinical care, and the need for innovative solutions. Furthermore, it seems especially urgent to emphasize a preventative approach, screening for adherence barriers before they impact clinical outcomes. Patient-reported outcome measures could fulfill this function and potentially also contribute to more person-centered ART adherence management.

The I-Score Study

As a part of the Canadian HIV Clinical Trials Network (CTN) study CTN283, our team developed a short, 7-item patient-reported instrument (The I-Score) to measure perceived ART adherence barriers in routine HIV care to foster their systematic and streamlined assessment and improve their management. The "I" in I-Score refers to the concept of "minimal interference" in life to summarize HIV expert opinions on characteristics of the ideal antiretroviral therapy, as analyzed by our team in earlier work[17], which helped inspire the measure's development. The I-Score was mainly intended for electronic administration in clinical practice (for example, via a patient portal) in Montreal

(Quebec) and Paris (France) among adults with HIV on daily oral ART. However, hard copy administration remains an option.

Further justification for the development of this measure comes from our team's evaluation of existing patient-reported measures of barriers to ART adherence, which were found to lack comprehensiveness,[18] and our needs assessment for the features of a patient portal among PLHIV and healthcare professionals (n=114) in the target settings;[19] this survey-based assessment identified that 90% or more of both groups were interested in portal-based administration of patient-reported measures focused on the experience of treatment and HIV self-management, categories within which the I-Score falls. Furthermore, this instrument' development benefited from the input of a patient advisory committee, the I-Score Consulting Team, the early activities of which are presented elsewhere.[20]

Over the years, during this multi-phase, multi-site instrument development project (the I-Score Study), we have accumulated evidence to support the tool's validity for our context of use. With this manuscript, our overarching objective is to describe this patient-reported outcome measure's (PROM) development process to date and its results. We will present the methods and findings related to its: a) conceptual framework; b) cognitive testing; and c) measurement properties. The instrument was generated in English and subsequently translated to French, and both language versions were evaluated for suitability for use in both Canadian/Quebec French or English and France French contexts.

Details on our translation approach are published elsewhere.[21] The general components of the I-Score's development process are presented in Fig 1.

Figure 1. The 7-item I-Score's development process to date.

Patient and public involvement

From this project's inception, people with HIV have been regularly consulted as advisory committee members[20] and/or as patient-partners (e.g., co-author SV). They have been repeatedly and meaningfully engaged in the planning (e.g., giving input on data collection tools), conduct (e.g., participating in and providing feedback on analyses), and knowledge translation of the work produced through this project. Involved people with HIV have co-authored related peer-reviewed publications on their experience[22-23] and presented at HIV conferences. All participating people with HIV who were part of our involvement activities in the I-Score Study gave their informed and written consent.

Ethics approval for the I-Score Study

The I-Score Study obtained ethics approval from the Research Ethics Board (REB) of the McGill University Health Centre (MUHC) in Montreal, Quebec, Canada (original approval received December 1, 2014: 2015-2331, 14-229 PSY). In addition to annual renewals until November 29, 2023, two subsequent amendments were also approved by the MUHC REB (an amendment to the I-Score measure's validation plan: approved January 18, 2022; an amendment for the retrospective analysis of viral load test results of patients with HIV at the Chronic Viral Illness Service of the McGill University Health Centre, waiving the requirement for informed consent: approved September 21, 2023). For the Toronto site (St. Michael's Hospital), REB approval was originally obtained from Unity Health Toronto, Toronto, Ontario, Canada, on November 10, 2016 (REB# 16-104) and renewed until November 10,

2023. For the affiliated French sites, approval was obtained from the REB of the Nantes University Health Centre in Nantes, France, on March 6, 2018 (RC17_0310 N° EUDRACT: 2017-A02460-53; French legislation requires no subsequent annual renewals). The project to meaningfully involve people with HIV in the I-Score Study, in part through a patient advisory committee, was approved by the MUHC REB on September 8, 2015 (project number: 15-188-MUHC).

The conceptual framework

To inform item generation (content elicitation) for the I-Score, a conceptual framework was developed by the team. For this purpose, as we were especially interested in the patient perspective on barriers to ART adherence, we conducted, with a librarian-guided search strategy, a qualitative synthesis (using thematic analysis) of 41 studies employing qualitative methods among adults with HIV on ART in developed countries.[24] The resultant conceptual framework has six domains and 20 subdomains of barriers. The six domains are: Cognitive and emotional aspects ("Your thoughts and feelings"), Lifestyle factors ("Your activities"), Social and material context ("Your situation"), Characteristics of ART ("Your medication"), Health experience and state ("Your health"), and Healthcare services and system ("Your care"). Saturation levels for all domains and subdomains are reported in the associated publication.[21] These show that from 66% to 100% of the sample of qualitative studies contributed content to each domain. A color figure of the conceptual framework is also available.[25] Interestingly, its domains overlap considerably with those of the World Health Organization (WHO) model of factors affecting adherence to long-term therapy,[26] discovered after our framework's creation. Key distinctions include our separation of the WHO's Patient-related factors into two domains (Cognitive and emotional aspects; Lifestyle factors), and our inclusion of stigma within the Social and material context, instead of within Patient-related factors.

When we mapped the content of existing patient-reported measures of barriers to ART adherence to our conceptual framework, we observed that, on average, they covered 4 of our 6 domains and only 7 of our 20 subdomains.[18] The least frequently addressed was the Healthcare services and system domain, with only a fifth (22%) of the 31 instruments reviewed including any related content. This is consistent with the limited comprehensiveness observed in generic measures of medication adherence barriers[27] and the relative underrepresentation of healthcare team and system-related barriers, across adherence barrier measures.[28] Average length of the reviewed instruments was also 14 items which may be longer than ideal for use across care settings.[9]

While our qualitative synthesis identified over 70 distinct barriers to ART adherence,[24] a lengthy measure would not be feasible for routine administration in HIV care (while it may be more appropriate for research purposes which our team intends to explore). For this specific context of use, we therefore drafted one item per framework domain to essentially capture the presence of barriers within a given domain. We defined barrier domains as "areas of life" that "make it difficult to take" ART and difficulty was defined as medication-taking that "was either skipped, delayed or very unpleasant." After several rounds of team feedback (including representation of healthcare professionals, measurement specialists, people with HIV, and research staff), the English and French measures were submitted to cognitive testing.

Cognitive testing

Objective and methods

The main goals of cognitive testing were to assess the measure's comprehensiveness, relevance, and comprehensibility from the perspective of people with HIV, to ensure content validity. Purposive sampling within adult clinical settings (Chronic Viral Illness Service-McGill University Health Centre in Montreal, Quebec and Service des maladies infectieuses et tropicales-Saint-Antoine Hospital, Assistance Publique-Hôpitaux de Paris (AP-HP), in Paris, France) was used to identify 12 diverse cognitive interview participants to ensure adequate representation of the three main interest groups (4 participants each): 1) francophone Quebeckers; 2) anglophone Quebeckers; and 3) francophone Parisians. A qualified member of the research team obtained written informed consent and conducted the semi-structured interviews in either English or French (DL), following a predefined script (S1 File), to receive input on the measure's content, including its instruction[illegible]he six items, response format, and recall period; to assess comprehension; and to gauge perceived comprehensiveness, relevance, and acceptability. All interviews were audio-recorded and transcribed verbatim. Content analysis was used to categorize the[illegible]ormation collected and identify areas for improvement. Independent coding by two individuals was done with one focus group transcript to calibrate the coding scheme. Analysis was performed with Taguette,[29] [illegible] free, open-source qualitative research tool.

Results

The cognitive interviews, individually or by focus group, were held from September 23 to November 16, 2020. They include[illegible]hree focus groups with from 2 to 4 participants, and three individual interviews. Each lasted from 102 to 133 minutes in duration.

Interview participants included nine males and three females, all born between 1992 and 1956. Some descriptive data was missing for one male. Five lived in the Montreal area (Quebec), one in Pointe-Claire (Quebec), and four in Paris (France). Six participants identified as homosexual, four as heterosexual and one as bisexual. Four were anglophone and the remainder, francophone. More than half (7) reported university as their highest level of education, while three reported college and one, high school. Half reported immigrating to their country of residence. None had ever injected drugs, and all were satisfied with their current HIV medication. The participants had been living with HIV from a little over 1 to 35 years (M =13.3, SD = 11.8).

Areas for improvement were identified for item ordering, item content, the response scale, the instructions, and the recall period. These are summarized in Table 1. The analyses were discussed at a team meeting, leading to the described implemented changes. Notably, after this stage, the measure increased by 1 item to 7 items.

Table 1. Changes implemented to the I-Score following cognitive testing.

**Aspect**	**Summary of participant reasoning (when provided)**	**Cognitive interview session***						**Implemented change**
		1	2	3	4	5	6	
**Response**	Gradations in the response scale would be better	x		x				We transformed the

Aspect	Summary of participant reasoning (when provided)	Cognitive interview session*						Implemented change
		1	2	3	4	5	6	
**format**	(from 0 or 1 to 10) as they are customary, facilitate answering and/or allow for greater precision.							response format from a continuum to an 11-point scale from 0 to 10.
**Instructions**	Gradations in the response scale would be better (from 0 or 1 to 10) as they are customary, facilitate answering and/or allow for greater precision.	x		x				We revised the instructions to match the new response format (from “Mark your response on the line” to “Answer with a number from 0 (Never made it difficult) to 10 (Always made it difficult).
**Recall period**	The timeframe (i.e., the past 4 weeks) is too short to capture some barriers (e.g., winter travel) or for experienced treatment-takers. Other timeframes are preferable (e.g., past year, past 6 months, past 3 months, past 2 months, since your last consultation).	x	x			x		We will reconsider the measure’s recall period when next implementing the I-Score in HIV care.
**Item order**	All other answers depend on the acceptance or not of one’s HIV status [which is addressed in item 2, “Your thoughts and feelings”].			x			x	We shifted item 2 (Your thoughts and feelings) up to become the first item.
	Items 4 and 6 are related.			x				We put items 4 (Your medication) and 6 (Your care) one after the other.
	Items 1 and 3 are related.			x				We put items 1 (Your habits and activities) and 3 (Your situation) one after the other.
**Content of item 2** (Your thoughts and feelings)	All other answers depend on the acceptance or not of one’s HIV status [which is addressed in item 2, Your thoughts and feelings].						x	In item 2, we made “acceptance of having HIV” the first example barrier provided.
**Content of item 3** (Your situation)	The item is hard to answer/too long, given the many issues covered within this domain. Similar issues (relationships and stigma vs housing and finances) can be grouped together in separate items.	x		x			x	We divided item 3 into two items, one for social and stigma-related barriers and one for financial and housing-related barriers.
	The domain title “Your situation” is imprecise. Relationship and stigma barriers can be labelled “Your social situation.”			x				We qualified the domain title. As item 3 was divided into two items, their domain titles became “Your social situation” and Your economic situation.”

Aspect	Summary of participant reasoning (when provided)	Cognitive interview session*						Implemented change
		1	2	3	4	5	6	
**Content of item 5** (Your health)	The French expression 'santé globale' is preferable.			x				In the French version, we replaced 'santé générale' by 'santé globale.' For consistency, in the English version, we changed 'general health' to 'overall health.'
	The item is difficult to answer or understand (or could be for some people). The meaning or role of the specific barriers, especially, that of 'infection-related symptoms' is not clear. The item should be rewritten.	x	x			x		We reformulated the item to simplify the language and refer directly to 'HIV symptoms.'

*Session 1: Sept. 23 2020 (4 participants); Session 2: Sept. 24 2020 (2 participants); Session 3: Oct. 5 2020 (1 participant); Session 4: Nov. 5 2020 (1 participant); Session 5: Nov. 9 2020 (1 participant); Session 6: Nov. 16 2020 (3 participants).

Measurement properties

The 7-item I-Score corresponds to a formative measurement model, where, for instance, the items are not expected to be highly correlated or interchangeable. Therefore, some sources of evidence (e.g., related to internal consistency) are not required to establish measure quality.[30]

Objectives

Our specific objectives, at this point, were to: assess the 7-item I-Score's structural validity, concurrent and predictive validities, reliability, and measurement error. We also considered measurement invariance and cross-cultural validity by examining the influence of several sociodemographic factors and mode of survey administration on evidence of the I-Score's concurrent and predictive validities.

Methods

Design and sample

This online survey-based study followed a prospective, longitudinal design with two periods of observation (Time 1: baseline and Time 2: Week 4). Eligible participants self-identified as at least 18 years old, living with HIV, currently on antiretroviral therapy, and a resident of Canada or France. At least 25% of the sample needed to be composed of self-identified women (whether cis- or trans- gender) to ensure their adequate representation.[31] Convenience sampling was used. Participants were recruited by the staff of participating healthcare settings (McGill University Health Centre (Montreal, Canada); St. Michael's Hospital (Toronto, Canada); Saint-Antoine Hospital (Paris, France)) and HIV community-based organizations (e.g., Gap-Vies, Portail VIH/Sida du Québec (Montreal, Canada)). Written informed consent was obtained from all participants. The targeted sample size was based on a 5:1 ratio of participants to evaluated measure items, deemed adequate by some statisticians for principal components analysis (PCA)[32, 33] (5 participants x 49 items = 245 participants). Note that along with the

7-item I-Score which assesses barrier domains, 42 additional items on specific barriers identified by our qualitative synthesis[24] and retained from a Delphi consensus process[21, 25] were administered as well and will be eventually submitted to PCA to develop additional instruments for research and/or care (results not presented).

Data collection

The surveys were built and managed on Research Electronic Data Capture (REDCap),[31] a secure web application. From January 19, 2022, to February 21, 2023, participants completed the 7-item I-Score, at baseline (Time 1) and 4 weeks later (Time 2). Note that recruitment from the Toronto site began November 14, 2022. Four self-reported adherence-related variables were also measured, for hypothesis testing, at each ti[illegible] point: 1) ART adherence in the last 30 days, using a continuous scale from 0% to 100%;[35] 2) ART adherence in the last 7 days,[36] using an ordinal scale from 1 (=all HIV pills taken) to 5 (=no HIV pills taken); 3) intention to adhere to ART,[37] measured by the average of three questions, using an ordinal scale from 1 (=strongly disagree) to 5 (=strongly agree), where Cronbach alpha for this instrument in our sample was 0.87; and 4) self-reported last viral load test result (a measure created for this study), using a nominal scale with three categories ("undetectable", "detectable", "I don't know"). For participants recruited at the Chronic Viral Illness Service of the McGill University Health Centre in Montreal, we extracted anonymized plasma HIV viral load test results (number of HIV copies per milliliter of blood) from the patients' medical files that were closest to each timepoint (retrieved March 20, 2024). Note that the median time elapsed between the Time 1 and Time 2 plasma viral load test results for these participants was 123.5 days (approximately 4 months' duration). The choice of the dependent variables reflects the lack of a gold standard for ART adherence measurement[38] and for most PROMs,[39] hence our inclusion of a mix of indirect 'subjective' and 'objective'[40] measures of adherence. The following categorical sociodemographic characteristics of the participants were also collected: selected survey language (English or French) as well as self-reported country of residence (Canada or France), immigration status (i.e., whether born outside the country of residence or not), age in years, level of education, sex given at birth, and sexual orientation. Furthermore, the mode of survey completion was recorded, especially, whether self- or other-administered.

Main study variables

In the logistic regression analyses (described in the next section), the I-Score's seven items served as independent variables and all five dependent variables were dichotomized: 1) ART adherence in the last 30 days was coded 1 if its value was greater or equal to 95% (a commonly used optimal ART adherence threshold)[41] and to 0 otherwise; 2) ART adherence in the last 7 days was coded 1 if its value was 1 (=all HIV pills taken) and 0 otherwise; 3) intention to adhere to ART was coded 1 if its value was an average of 5 and 0 if less than 5; 4) self-reported viral load was set to 1 if its category was "undetectable" and 0 otherwise; and 5) plasma viral load test results were coded 1 if the number of HIV copies per mL of blood was less than 20 (recognized as an optimal level of viral suppression)[42] and 0 otherwise.

Statistical analyses

All analyses were performed with R (version 4.3.1).[43] Descriptive statistics are provided for the participants' sociodemographic characteristics, mode of survey administration, and the independent and dependent variables, including when stratified by the characteristics of interest. Missing data are reported for all variables. All provided *p*-values respect standards for reporting and interpreting statistical significance in medical research.[44]

As an indicator of structural validity, the redundancy of the I-Score's seven items was evaluated with inter-item Pearson correlations at Time 1, with strongly correlated items (i.e., a correlation coefficient of at least 0.80), considered for possible elimination.[45] In addition, investigating whether each I-Score item represents a unique and independent dimension of barriers to ART adherence, we performed a principal component analysis (PCA) on the inter-item correlation matrix at both Time 1 and Time 2. We considered seven principal components and extracted the corresponding loadings. If the associations between the principal components and the I-Score's seven items were difficult to interpret, we applied a varimax rotation which aims to improve interpretation of the loadings, compelling them to be either highly positive (or negative) or zero, while keeping the components uncorrelated. The rotation makes the loadings more extreme, so that the high and low correlations between the I-Score items and the principal components can be more easily identified. Structural validity was indicated if each of the I-Score's seven item was strongly (loading of ≥ 0.80) and independently associated with only one of the seven principal components.

To evaluate the concurrent validity of the I-Score's seven items, five logistic regressions were fitted to the data, one for each dichotomized dependent variable, using only Time 1 data. All seven items of the I-Score were considered covariates. Covariate significance was set at a p-value of $\alpha < 5\%$. We also calculated the receiver operating characteristic (ROC) curve for each logistic regression, and the corresponding area under the curve (AUC). Note that AUC values range from 0 to 1, where 1 indicates a model that predicts with 100% accuracy. To classify the predictive capacity of each logistic regression based on the corresponding AUC, we used the thresholds proposed by Hosmer et al.[46] ($0.5 < \text{AUC} < 0.70$ = 'Poor'; $0.70 \leq \text{AUC} < 0.80$ = 'Acceptable'; $0.8 \leq \text{AUC} < 0.9$ = 'Excellent'; $\text{AUC} \geq 0.9$ = 'Outstanding') and Swets[47] (AUC: < 0.70 = 'Low'; $0.70 \leq \text{AUC} < 0.90$ 'Useful'; $\text{AUC} \geq 0.90$ = 'High' accuracy). Evaluation of the I-Score's predictive validity proceeded as described for testing its concurrent validity, while using Time 2 data for the dependent variables. Subgroup analyses were performed to evaluate the sensitivity of the models to the participants' sociodemographic characteristics and the mode of survey administration (to determine measurement invariance).

To assess the I-Score's reliability, we employed a test-retest reliability approach, a common methodology used in the development of psychometric tools. Test-retest reliability indicates the degree to which scale scores obtained from the same participants remain consistent over a short time interval (in our case from Time 1 to Time 2, 4 weeks later). With the unrotated PCA performed on the inter-item correlation matrix at both Times 1 and 2 for the I-Score's structural validity, we summarized the information contained in its seven items by retaining principal components based on Kaiser's criterion (i.e., those corresponding to an eigenvalue greater than 1). We also validated the number of retained principal components with a scree plot, selecting the number that appears prior to the plot's elbow. We expected to find a single principal component that explains a large proportion of the variance in the data, with similar loadings across the I-Score's seven items. Once each participant's score was obtained by

summing the products of each loading with the corresponding I-Score item's standardized response, test-retest reliability was then assessed with the intraclass correlation coefficient (ICC), computed from the obtained scores. Following COnsensus-based Standards for the selection of health Measurement INstruments (COSMIN)[30] recommendations, the ICC was calculated with a two-way random effects model, with good reliability indicated by an ICC ≥ 0.70.

To estimate measurement error, we followed COSMIN[30] recommendations to use the standard error of measurement (SEM) for continuous scores based on a test-retest design. The SEM, a statistic obtained from the ICC, estimates how a participant's repeated measures with the same instrument tend to be distributed around his/her "real" score. The SEM directly relates to the reliability of an instrument: the larger the SEM, the lower the reliability, and the lower the precision of measurement. The SEM had a possible range of 0 to 1, with a SEM closer to 0 considered more favorable.

Working hypotheses and performance thresholds

As mentioned, for our purposes, adequate structural validity would be indicated by Pearson inter-item correlation coefficients below 0.80 (absence of redundant items) as well as high (≥ 0.80) and independent factor loadings of the items on each of the 7 components of the PCA.

For issues of construct validity, our hypotheses were as follows:

a) With the global sample, at least one I-Score item (covariate) would be statistically associated with each dependent variable in at least 75% of the logistic regression analyses (excluding inconclusive results).

b) With the global sample, the I-Score's accuracy would be at least "Acceptable; Useful" (AUC ≥ 0.70) for at least 75% of the analyses for each dependent variable (excluding inconclusive results).

c) For each subgroup examined, the I-Score's accuracy would be at least "Acceptable; Useful" (AUC ≥ 0.70) for at least 75% of the analyses across the dependent variables (excluding inconclusive results).

The choice of a 75% threshold was based on COSMIN criteria for the sufficiency of results in hypothesis or responsiveness testing to determine a PROM's measurement properties.[30]

Finally, to review, reliability would be indicated by an ICC of ≥ 0.70 and low measurement error, by a SEM closer to 0.

Results

Table 2 shows the sample's sociodemographic information. A total of n=305 participants were included in the analyses, 31% of whom were female, indicating that we surpassed our sample size and sex-based recruitment goals.

Table 2. Sociodemographic characteristics of the sample (n=305).

	n (%)
**Survey language**	

English	83 (27.2%)
French	222 (72.8%)
**Country of residence**	
Canada	261 (85.6%)
France	41 (13.4%)
Missing	3 (1.0%)
**Immigration status**	
Immigrant	161 (46.5%)
Non-immigrant	142 (52.8%)
Missing	2 (0.7%)
**Age (years)**[1]	
< 50	135 (44.2%)
≥ 50	160 (52.5%)
Missing	10 (3.3%)
**Level of education**	
Primary/Elementary	19 (6.2%)
Secondary (High school)/Professional degree	109 (35.8%)
College (post-secondary)/CEGEP/ Technical degree	59 (19.3%)
University	104 (34.1%)
Other	8 (2.6%)
Missing	6 (2.0%)
**Sex**	
Female	94 (30.8%)
Male	207 (67.9%)
Missing	4 (1.3%)
**Sexual orientation**	
Heterosexual	119 (39.0%)
Homosexual	137 (44.9%)
Bisexual	22 (7.2%)

Other	5 (1.7%)
Prefer not to answer	18 (5.9%)
Missing	4 (1.3%)

[1] Mean (SD) = 50.4 (13.0); Range: 19.0-82.0; for n=295 participants.

S1 and S2 Tables display the descriptive statistics of the previously described independent and dependent variables at Times 1 and 2 and stratified by the sample's sociodemographic characteristics, and the survey's mode of administration.

Structural validity

Table 3 shows the Pearson correlation coefficients for all pairs of the I-Score items. Coefficients ranged from 0.29 to 0.62. As none was greater than or equal to 0.80, no item was considered for elimination.

Table 3. Pearson inter-item correlation matrix.

**I-Score item**	Thoughts and feelings	Habits and activities	Social situation	Economic situation	Medication	Care
Habits and activities	0.51	-	-	-	-	-
Social situation	0.62	0.39	-	-	-	-
Economic situation	0.49	0.36	0.56	-	-	-
Medication	0.50	0.33	0.44	0.47	-	-
Care	0.44	0.29	0.43	0.39	0.36	-
Health	0.50	0.31	0.40	0.34	0.50	0.48

Tables 4 and 5 show the loadings of the seven principal components at Time 1 and Time 2 respectively, obtained after a varimax rotation. The highest loadings of each principal component are highlighted in bold.

Table 4. Loadings at baseline (Time 1) between the seven principal components obtained by a varimax rotation and the I-Score's seven items.

	PC1	PC2	PC3	PC4	PC5	PC6	PC7
Thoughts and feelings	0.27	0.21	0.25	0.20	0.17	0.19	**0.84**
Habits and activities	0.13	0.11	**0.95**	0.11	0.10	0.13	0.18
Social situation	**0.88**	0.14	0.16	0.16	0.18	0.25	0.24
Economic situation	0.23	0.11	0.14	0.19	0.15	**0.91**	0.16
Medication	0.15	0.21	0.12	**0.92**	0.13	0.19	0.16

Care	0.15	0.20	0.11	0.12	**0.93**	0.14	0.14
Health	0.13	**0.92**	0.12	0.21	0.21	0.11	0.17

Table 5. Loadings at Week 4 (Time 2) between the seven principal components obtained by a varimax rotation and the I-Score's seven items.

	PC1	PC2	PC3	PC4	PC5	PC6	PC7
Thoughts and feelings	**0.84**	0.28	0.21	0.26	0.17	0.21	0.20
Habits and activities	0.23	**0.90**	0.22	0.14	0.15	0.20	0.10
Social situation	0.18	0.21	0.14	0.20	0.20	**0.88**	0.23
Economic situation	0.19	0.11	0.22	0.20	0.23	0.23	**0.87**
Medication	0.24	0.16	0.19	**0.86**	0.24	0.21	0.21
Care	0.15	0.17	0.22	0.24	**0.86**	0.20	0.23
Health	0.19	0.23	**0.87**	0.18	0.21	0.15	0.22

For both timepoints, each principal component has only one high loading (ranging from 0.84 to 0.95) while the remaining ones are very low (ranging from 0.10 to 0.28). Each of the I-Score's seven item is thus strongly and uniquely associated with only one principal component, without any overlap between components.

Concurrent and predictive validity

Table 6 presents the results of the ten logistic regressions for the global sample and stratified by the considered sociodemographic characteristics and mode of survey administration to test the I-Score's concurrent (Time 1 to Time 1) and predictive (Time 1 to Time 2) validity. Inconclusive results, identified in the table, are due to the lack of convergence of the iterative procedure to obtain the regression coefficients' estimates or to abnormally high values for the estimated standard errors of the regression coefficients caused mainly by small subgroup sizes.

Table 6. Significant covariates and areas under the ROC curve (AUC) of the ten logistic regressions, with the interpretation thresholds of Hosmer et al. (H) and Swets (S), for the global sample and stratified by sociodemographic group and mode of survey administration.

		ART adherence/last 30 days		ART adherence/last 7 days		Intention to adhere to ART		Self-reported viral load		Plasma viral load test result	
		Significant covariates	AUC (H; S)	Significant covariates	AUC (H; S)	Significant covariates	AUC (H; S)	Significant covariates	AUC (H; S)	Significant covariates	AUC (H; S)
**Global sample** (n=305)	Time 1 to Time 1	Habits and activities (p=0.02)	0.68 (Poor; Low)	Habits and activities (p<0.001)	0.72 (Acceptable; Useful)	Economic situation (p=0.04)	0.66 (Poor; Low)	Habits and activities (p=0.001) Health (p=0.003)	0.80 (Excellent; Useful)	Habits and activities (p=0.02)	0.74 (Acceptable; Useful)
	n (missing)	284 (21)		297 (8)		296 (9)		285 (20)		108 (197)	
	Time 1 to Time 2	Habits and activities (p<0.001)	0.73 (Acceptable; Useful)	-	0.66 (Poor; Low)	-	0.65 (Poor; Low)	Health (p=0.03)	0.75 (Acceptable; Useful)	Health (p=0.01)	0.77 (Acceptable; Useful)
	n (missing)	230 (75)		241 (64)		241 (64)		234 (71)		72 (233)	
**Survey language** English (n=83)	Time 1 to Time 1	Thoughts and feelings (p=0.03) Habits and activities (p=0.01)	0.86 (Excellent; Useful)	Habits and activities (p=0.008)	0.83 (Excellent; Useful)	Habits and activities (p=0.006)	0.74 (Acceptable; Useful)	Habits and activities (p=0.04)	0.88 (Excellent; Useful)	-	0.77 (Acceptable; Useful)
	n (missing)	79 (4)		81 (2)		81 (2)		76 (7)		36 (47)	
	Time 1 to Time 2	Habits and activities (p=0.002)	0.90 (Outstanding; Highly accurate)	Habits and activities (p=0.002) Care (p=0.01)	0.90 (Outstanding; Highly accurate)	-	0.73 (Acceptable; Useful)	Inconclusive		Inconclusive	
	n (missing)	59 (24)		62 (21)		62 (21)		59 (24)		27 (56)	
French (n=222)	Time 1 to Time 1	-	0.66 (Poor; Low)	Habits and activities (p=0.04)	0.67 (Poor; Low)	-	0.66 (Poor; Low)	Habits and activities (p=0.006) Health	0.78 (Acceptable; Useful)	-	0.72 (Acceptable; Useful)

		ART adherence/last 30 days		ART adherence/last 7 days		Intention to adhere to ART		Self-reported viral load		Plasma viral load test result	
		Significant covariates	AUC (H; S)	Significant covariates	AUC (H; S)	Significant covariates	AUC (H; S)	Significant covariates	AUC (H; S)	Significant covariates	AUC (H; S)
								(p=0.02)			
	n (missing)	205 (17)		216 (6)		215 (7)		209 (13)		72 (150)	
	Time 1 to Time 2	-	0.66<br>(Poor; Low)	Thoughts and feelings (p=0.01)	0.70<br>(Acceptable; Useful)	-	0.65<br>(Poor; Low)	Health (p=0.04)	0.80<br>(Excellent; Useful)	Health (p=0.03)	0.77<br>(Acceptable; Useful)
	n (missing)	171 (51)		179 (43)		179 (43)		175 (47)		45 (177)	
**Country of residence**<br>Canada (n=261)	Time 1 to Time 1	Habits and activities (p=0.007)	0.70<br>(Acceptable; Useful)	Habits and activities (p<0.001)	0.73<br>(Acceptable; Useful)	Habits and activities (p=0.04)	0.65<br>(Poor; Low)	Habits and activities (p=0.001)<br>Health (p=0.003)	0.80<br>(Excellent; Useful)	Habits and activities (p=0.02)	0.74<br>(Acceptable; Useful)
	n (missing)	241 (20)		253 (8)		252 (9)		241 (20)		108 (153)	
	Time 1 to Time 2	Habits and activities (p<0.001)	0.75<br>(Acceptable; Useful)	-	0.67<br>(Poor; Low)	-	0.61<br>(Poor; Low)	Health (p=0.04)	0.76<br>(Acceptable; Useful)	Health (p=0.01)	0.77<br>(Acceptable; Useful)
	n (missing)	195 (66)		205 (56)		205 (56)		197 (64)		72 (189)	
France (n=41)	Time 1 to Time 1	-	0.73<br>(Acceptable; Useful)	Inconclusive		-	0.85<br>(Excellent; Useful)	Inconclusive		-	-
	n (missing)	41 (0)		41 (0)		41 (0)		41 (0)		0 (0)	
	Time 1 to Time 2	-	0.91<br>(Outstanding; Highly accurate)	Inconclusive		Inconclusive		Inconclusive		-	-
	n (missing)	33 (8)		34 (7)		34 (7)		34 (7)		0 (0)	
**Immigration status**<br>Immigrant	Time 1 to Time 1	-	0.68	Medication (p=0.04)	0.81<br>(Excellent;	Medication (p=0.03)	0.65	Habits and activities	0.86<br>(Excellent;	Habits and activities	0.75<br>(Acceptable;

		ART adherence/last 30 days		ART adherence/last 7 days		Intention to adhere to ART		Self-reported viral load		Plasma viral load test result	
		Significant covariates	AUC (H; S)	Significant covariates	AUC (H; S)	Significant covariates	AUC (H; S)	Significant covariates	AUC (H; S)	Significant covariates	AUC (H; S)
(n=161)			(Poor; Low)	Care (p=0.007) Health (p=0.02)	Useful)		(Poor; Low)	(p=0.02)	Useful)	(p=0.04)	Useful)
	n (missing)	146 (15)		156 (5)		154 (7)		147 (14)		70 (91)	
	Time 1 to Time 2	-	0.70 (Acceptable; Useful)	-	0.68 (Poor; Low)	-	0.70 (Acceptable; Useful)	-	0.72 (Acceptable; Useful)	-	0.85 (Excellent; Useful)
	n (missing)	109 (52)		117 (44)		117 (44)		114 (47)		41 (120)	
Non-immigrant (n=142)	Time 1 to Time 1	Habits and activities (p=0.01)	0.74 (Acceptable; Useful)	Habits and activities (p<0.001) Medication (p=0.02)	0.82 (Excellent; Useful)	-	0.70 (Acceptable; Useful)	Health (p=0.01)	0.78 (Acceptable; Useful)	Inconclusive	
	n (missing)	136 (6)		139 (3)		140 (2)		136 (6)		38 (104)	
	Time 1 to Time 2	Habits and activities (p=0.004)	0.72 (Acceptable; Useful)	Habits and activities (p=0.005)	0.74 (Acceptable; Useful)	-	0.68 (Poor; Low)	Habits and activities (p=0.01)	0.88 (Excellent; Useful)	-	0.82 (Excellent; Useful)
	n (missing)	120 (22)		123 (19)		123 (19)		118 (24)		31 (111)	
**Age (years)**											
< 50 (n=135)	Time 1 to Time 1	-	0.66 (Poor; Low)	Habits and activities (p=0.006) Medication (p=0.04) Care (p=0.03)	0.80 (Excellent; Useful)	Economic situation (p=0.02)	0.72 (Acceptable; Useful)	Inconclusive		Habits and activities (p=0.04)	0.84 (Excellent; Useful)
	n (missing)	124 (11)		131 (4)		131 (4)		126 (9)		47 (88)	
	Time 1 to Time 2	Habits and activities (p<0.001)	0.82 (Excellent; Useful)	-	0.71 (Acceptable; Useful)	-	0.71 (Acceptable; Useful)	Inconclusive		Inconclusive	

		ART adherence/last 30 days		ART adherence/last 7 days		Intention to adhere to ART		Self-reported viral load		Plasma viral load test result	
		Significant covariates	AUC (H; S)	Significant covariates	AUC (H; S)	Significant covariates	AUC (H; S)	Significant covariates	AUC (H; S)	Significant covariates	AUC (H; S)
	n (missing)	104 (31)		106 (29)		105 (30)		104 (31)		34 (101)	
≥ 50 (n=160)	Time 1 to Time 1	Thoughts and feelings (p=0.01) Social situation (p=0.04) Care (p=0.03)	0.74 (Acceptable; Useful)	Habits and activities (p=0.02)	0.80 (Excellent; Useful)	-	0.65 (Poor; Low)	Habits and activities (p=0.006) Health (p=0.002)	0.90 (Outstanding; Highly accurate)	-	0.72 (Acceptable; Useful)
	n (missing)	152 (8)		156 (4)		155 (5)		150 (10)		57 (103)	
	Time 1 to Time 2	Thoughts and feelings (p=0.007)	0.70 (Acceptable; Useful)	Thoughts and feelings (p=0.03)	0.70 (Acceptable; Useful)	-	0.61 (Poor; Low)	-	0.83 (Excellent; Useful)	-	0.80 (Excellent; Useful)
	n (missing)	118 (42)		127 (33)		128 (32)		121 (39)		37 (123)	
**Level of education**											
Secondary (High school)/ Professional degree (n=109)	Time 1 to Time 1	Habits and activities (p=0.02)	0.70 (Acceptable; Useful)	-	0.70 (Acceptable; Useful)	-	0.62 (Poor; Low)	Habits and activities (p=0.02) Health (p=0.01)	0.86 (Excellent; Useful)	-	0.75 (Acceptable; Useful)
	n (missing)	104 (5)		107 (2)		107 (2)		102 (7)		37 (72)	
	Time 1 to Time 2	Thoughts and feelings (p=0.01)	0.90 (Outstanding; Highly accurate)	Thoughts and feelings (p=0.01)	0.78 (Acceptable; Useful)	-	0.65 (Poor; Low)	-	0.80 (Excellent; Useful)	Inconclusive	
	n (missing)	75 (34)		83 (26)		83 (26)		81 (28)		25 (84)	
College (post-secondary)/CEGEP/ Technical degree (n=59)	Time 1 to Time 1	-	0.70 (Acceptable; Useful)	Health (p=0.03)	0.84 (Excellent; Useful)	-	0.68 (Poor; Low)	Inconclusive		Inconclusive	
	n (missing)	53 (6)		55 (4)		55 (4)		52 (7)		21 (38)	

		ART adherence/last 30 days		ART adherence/last 7 days		Intention to adhere to ART		Self-reported viral load		Plasma viral load test result	
		Significant covariates	AUC (H; S)	Significant covariates	AUC (H; S)	Significant covariates	AUC (H; S)	Significant covariates	AUC (H; S)	Significant covariates	AUC (H; S)
	Time 1 to Time 2	-	0.77<br>(Acceptable; Useful)	-	0.85<br>(Excellent; Useful)	-	0.70<br>(Acceptable; Useful)	Inconclusive		Inconclusive	
	n (missing)	41 (18)		44 (15)		44 (15)		41 (18)		12 (47)	
University (n=104)	Time 1 to Time 1	-	0.78<br>(Acceptable; Useful)	Habits and activities (p=0.02)	0.84<br>(Excellent; Useful)	Economic situation (p=0.002)<br>Care (p=0.02)	0.78<br>(Acceptable; Useful)	Inconclusive		-	0.81<br>(Excellent; Useful)
	n (missing)	97 (7)		103 (1)		103 (1)		101 (3)		41 (63)	
	Time 1 to Time 2	Habits and activities (p=0.004)	0.81<br>(Excellent; Useful)	Habits and activities (p=0.02)	0.84<br>(Excellent; Useful)	-	0.71<br>(Acceptable; Useful)	Inconclusive		-	0.77<br>(Acceptable; Useful)
	n (missing)	89 (15)		89 (15)		89 (15)		86 (18)		31 (73)	
**Sex**											
Female (n=94)	Time 1 to Time 1	-	0.75<br>(Acceptable; Useful)	Habits and activities (p=0.04)	0.82<br>(Excellent; Useful)	-	0.60<br>(Poor; Low)	Thoughts and feelings (p=0.04)<br>Habits and activities (p=0.003)<br>Health (p=0.04)	0.90<br>(Outstanding; Highly accurate)	Inconclusive	
	n (missing)	78 (16)		88 (6)		87 (7)		83 (11)		29 (65)	
	Time 1 to Time 2	-	0.77<br>(Acceptable; Useful)	-	0.64<br>(Poor; Low)	-	0.67<br>(Poor; Low)	Inconclusive		Inconclusive	
	n (missing)	60 (34)		64 (30)		65 (29)		62 (32)		14 (80)	
Male (n=207)	Time 1 to Time 1	-	0.67<br>(Poor; Low)	Habits and activities (p=0.005)	0.74<br>(Acceptable;	-	0.70<br>(Acceptable;	-	0.80<br>(Excellent;	Habits and activities (p=0.02)	0.78<br>(Acceptable;

		ART adherence/last 30 days		ART adherence/last 7 days		Intention to adhere to ART		Self-reported viral load		Plasma viral load test result	
		Significant covariates	AUC (H; S)	Significant covariates	AUC (H; S)	Significant covariates	AUC (H; S)	Significant covariates	AUC (H; S)	Significant covariates	AUC (H; S)
					Useful)		Useful)		Useful)		Useful)
	n (missing)	202 (5)		205 (2)		205 (2)		198 (9)		77 (130)	
	Time 1 to Time 2	Habits and activities (p=0.002)	0.72 (Acceptable; Useful)	-	0.70 (Acceptable; Useful)	Thoughts and feelings (p=0.04)	0.71 (Acceptable; Useful)	-	0.71 (Acceptable; Useful)	Health (p=0.01)	0.83 (Excellent; Useful)
	n (missing)	167 (40)		174 (33)		173 (34)		168 (39)		56 (151)	
**Sexual orientation**											
Heterosexual (n=119)	Time 1 to Time 1	Habits and activities (p=0.01)	0.77 (Acceptable; Useful)	Habits and activities (p=0.006)	0.81 (Excellent; Useful)	Economic situation (p=0.01)	0.70 (Acceptable; Useful)	Habits and activities (p=0.006) Health (p=0.02)	0.83 (Excellent; Useful)	-	0.78 (Acceptable; Useful)
	n (missing)	109 (10)		113 (6)		112 (7)		112 (7)		34 (85)	
	Time 1 to Time 2	Thoughts and feelings (p=0.01)	0.82 (Excellent; Useful)	-	0.73 (Acceptable; Useful)	-	0.67 (Poor; Low)	Inconclusive		Inconclusive	
	n (missing)	83 (36)		88 (31)		88 (31)		86 (33)		19 (100)	
Homosexual (n=137)	Time 1 to Time 1	Health (p=0.01)	0.72 (Acceptable; Useful)	Habits and activities (p=0.04)	0.73 (Acceptable; Useful)	-	0.72 (Acceptable; Useful)	-	0.73 (Acceptable; Useful)	-	0.73 (Acceptable; Useful)
	n (missing)	132 (5)		135 (2)		135 (2)		133 (4)		51 (86)	
	Time 1 to Time 2	Habits and activities (p=0.002) Economic situation (p=0.04)	0.75 (Acceptable; Useful)	-	0.74 (Acceptable; Useful)	-	0.68 (Poor; Low)	-	0.71 (Acceptable; Useful)	Health (p=0.04)	0.80 (Excellent; Useful)
	n (missing)	116 (21)		121 (16)		121 (16)		116 (21)		41 (96)	
**Administration mode**	Time 1 to										

		ART adherence/last 30 days		ART adherence/last 7 days		Intention to adhere to ART		Self-reported viral load		Plasma viral load test result	
		Significant covariates	AUC (H; S)	Significant covariates	AUC (H; S)	Significant covariates	AUC (H; S)	Significant covariates	AUC (H; S)	Significant covariates	AUC (H; S)
Face-to-face interview (n=69)	Time 1 (n=69)	Habits and activities (p=0.002)<br>Care (p=0.04)	0.83 (Excellent; Useful)	Habits and activities (p=0.01)	0.88 (Excellent; Useful)	Economic situation (p=0.01)	0.72 (Acceptable; Useful)	Inconclusive		Inconclusive	
	n (missing)	66 (3)		69 (0)		68 (1)		65 (4)		15 (54)	
	Time 1 to Time 2 (n=53)	-	0.90 (Outstanding; Highly accurate)	-	0.73 (Acceptable; Useful)	-	0.82 (Excellent; Useful)	-	0.77 (Acceptable; Useful)	Inconclusive	
	n (missing)	47 (6)		47 (6)		47 (6)		47 (6)		2 (51)	
Self-administration online (n=184)	Time 1 to Time 1 (n=184)	-	0.72 (Acceptable; Useful)	-	0.78 (Acceptable; Useful)	-	0.72 (Acceptable; Useful)	Health (p=0.01)	0.77 (Acceptable; Useful)	Habits and activities (p=0.04)	0.80 (Excellent; Useful)
	n (missing)	175 (9)		179 (5)		179 (5)		172 (12)		62 (122)	
	Time 1 to Time 2 (n=166)	Habits and activities (p=0.001)	0.75 (Acceptable; Useful)	Habits and activities (p=0.009)	0.72 (Acceptable; Useful)	-	0.72 (Acceptable; Useful)	Health (p=0.001)	0.85 (Excellent; Useful)	Thoughts and feelings (p=0.03)<br>Habits and activities (p=0.04)<br>Health (p=0.01)	0.91 (Outstanding; Highly accurate)
	n (missing)	152 (14)		163 (3)		163 (3)		157 (9)		65 (101)	

For hypothesis (a), the results of the ten logistic regression analyses with the full sample show that in 8 (80%), at least one I-Score item emerged as statistically significant. Thus, we successfully attained our threshold. The three significant items were Habits and activities (5 analyses), Health (3), and Economic situation (1).

For hypothesis (b), again with the full sample, the results of the ROC analyses and AUC's indicate that at least "Acceptable; Useful" I-Score model performance (AUC ≥ 0.70) was obtained in at least 75% of analyses for self-reported viral load (100%; AUC: 0.75-0.80) and plasma viral load test results (100%: AUC: 0.74-0.77). Results were below the threshold for ART adherence in the last 30 days (50%; AUC: 0.68-0.73), ART adherence in the last 7 days (50%; AUC: 0.66-0.72) and intention to adhere to ART (0%; AUC: 0.65-0.66). Hence, we achieved partial success for hypothesis (b).

For hypothesis (c), subgroup analyses generated the following percentage of AUC ≥ 0.70 results, not including inconclusive results: English survey respondents (100%; 8/8), French survey respondents (50%; 5/10), residents of Canada (70%; 7/10); residents of France (100%; 3/3), immigrants (70%; 7/10), non-immigrants (89%; 8/9), individuals aged less than 50 (86%; 6/7), individuals aged 50 or over (80%; 8/10), those with a high school/secondary education (78%; 7/9), the college-educated (83%; 5/6), the university-educated (100%; 8/8), females (57%; 4/7), males (90%; 9/10), heterosexuals (88%; 7/8), homosexuals (90%; 9/10), those completing the survey by face-to-face interview (100%; 7/7), and those self-administering the survey online (100%; 10/10). Overall, in the subgroup analyses, for respondents completing the survey in French and females, the results were clearly below threshold. Results were considered borderline for Canadian residents and immigrants. Our requirements were, therefore, mostly met for hypothesis (c).

Considering the results obtained per dependent variable, across the global sample and subgroup analyses, large differences in the percentage of analyses indicating an acceptable performance of the I-Score models (i.e. AUC ≥ 0.70) were observed. For intention to adhere to ART, only 51% of AUC's were above threshold (18/35), compared with 83% for adherence in the past 30 days (30/36), 85% for adherence in the last 7 days (29/34), and 100% for both self-reported viral load (24/24) and plasma viral load test results (23/23). Without the intention to adhere variable, the 75% threshold for hypothesis (c) would be met for women (80%; 4/5), but not for French survey respondents (63%; 5/8). The results for Canadian residents (88%; 7/8) and immigrants (75%; 6/8) would also surpass the target and no longer be borderline.

Additionally, across the subgroup analyses, the percentage of AUC's ≥ 0.70 (not considering inconclusive results) was 84% (62/74) for concurrent validity testing (baseline data only) and 82% (56/68) for analyses assessing predictive validity. Equal percentages were found for analyses with the global sample (i.e. 60% in both cases), further suggesting similar accuracy with concurrent and future outcomes, as evaluated.

Reliability

Table 7 shows the eigenvalues extracted from the correlation matrix, for each principal component, at each measurement time. The eigenvalues greater than 1, according to Kaiser's criterion, are highlighted in bold.

Table 7. Eigenvalues of the correlation matrix for each principal component (PC), at Times 1 and 2.

Time	PC1	PC2	PC3	PC4	PC5	PC6	PC7
Baseline	**3.63**	0.80	0.69	0.64	0.50	0.42	0.33
Week 4	**4.25**	0.68	0.54	0.47	0.44	0.34	0.28

As expected, we retained only the first principal component at each timepoint. The scree plots at Time 1 and Time 2 supported this decision. This component explained 52% and 61% of the variance in the data at Time 1 and Time 2, respectively. Table 8 shows the loadings on the retained principal component of the I-Score's seven items, for each timepoint.

Table 8. Loadings between the first principal component and the I-Score's seven items, at Times 1 and 2.

Time	Thoughts and feelings	Habits and activities	Social situation	Economic situation	Medication	Care	Health
Baseline	0.82	0.62	0.78	0.72	0.71	0.67	0.70
Week 4	0.81	0.73	0.77	0.78	0.80	0.79	0.77

The loadings are similar for each timepoint, indicating a roughly balanced contribution of each item to score computation. Finally, we obtained an ICC of 0.81, which is above the threshold defined by COSMIN standards, establishing the measure's reliability.

Measurement error

As to measurement error, we obtained a SEM of 0.43, for which it is desirable to be closer to 0 than 1.

Discussion

Given its perceived utility, our team set out to create a short, yet comprehensive, patient-reported measure of barriers to ART adherence for administration as a part of routine HIV care (the 7-item I-Score). This manuscript summarizes the evidence accumulated to date for its use in urban cities in Canada (mainly Montreal and Toronto) and France (Paris) among adults with HIV on ART. We focused on the instrument's development from conceptual framework generation and cognitive testing to measurement property assessment. In the process, we produced evidence of the measure's content validity, construct validity, and reliability, as well as on its measurement error.

Conceptual framework

The conceptual framework,[24] grounded in the results of 41 qualitative studies among PLHIV in developed countries, provided a strong foundation from which to generate measure items. Encompassing ART adherence barriers reported by PLHIV within countries beyond those of Canada and France in the framework provides a basis to argue for the I-Score's potential application or adaptation to other contexts of use. This is further supported by the considerable overlap between the domains we identified and those of the WHO's generic model of factors affecting adherence to long-term therapy.[26]

That the I-Score measures barrier domains is a unique feature of our instrument relative to other ART adherence barrier measures. While the basis of its comprehensiveness and conciseness, assessing domains does not provide information on the patient's precise barrier(s). For instance, with this measure, a patient will rate the frequency of adherence difficulty within the domain of Thoughts and feelings which includes barriers tied to "acceptance of having HIV, emotions (feeling sad, anxious, etc.), medication-related knowledge and beliefs or motivation to take medication." This is intentional. Our PROM offers a relatively quick screen meant to instigate patient-healthcare provider communication, a well-supported outcome of PROM use in clinical practice,[48] that is expected, in turn, to pinpoint barriers and improve their management.

Cognitive testing

We consider that the cognitive testing performed on the instrument conforms to COSMIN standards for such content validation studies.[49] Importantly, it identified several issues that could negatively impact the measure's ease of use, logical flow, and comprehensibility. As a result, we integrated participant-recommended gradations to the response scale, from 0 to 10, with a clear midpoint. Such visual analogue scales have been used for over a century and are common metrics for PROMs,[50] including in ART adherence measurement.[51] The choice of this response scale is further supported by their reported advantages for health state measurement, including their simplicity, validity, reliability, practicality, feasibility, and acceptability.[52] Another significant change made to the I-Score was the addition of an item (due to separating an existing item (i.e. Your situation) into two (Your social situation, Your economic situation)). In a recent Cochrane review of ART adherence measures[9] intended to help identify instruments that could be used in resource limited clinical settings to identify patients in need of adherence support, instruments with more than 8 items were excluded. This suggests that, at 7 items, the I-Score's length remains feasible for use in HIV care. As to the measure's recall period, it was set at the past 4 weeks, a timeframe recommended for self-reported ART adherence measures.[53] However, several participants found this period too short for barriers to arise. For pragmatic reasons, it was maintained to concord with the recall period of one of the adherence measures used to evaluate the I-Score's measurement properties. In the future, when seeking the measure's formal implementation in HIV care, it will be reconsidered by the team in collaboration with stakeholders. Finally, no comprehensiveness issues were raised in relation to the measure. While in two separate data collection sessions, participants mentioned the absence of sexuality, and in one, the absence of religion, further discussion revealed that they were not understood as barriers to ART adherence but as important aspects of life that they did not see represented in the instrument.

Measurement properties

The statistical analyses conducted support the retention of each item of the I-Score. The inter-item correlation coefficients were generally in the weak to moderate range[54] and a PCA on the 7 items with a varimax rotation produced a set of 7 principal components, where each item was highly and independently associated with each component (a one-to-one correspondence). These findings argue for the dimensional independence of the items, thus providing evidence of the measure's structural validity.

Results on the concurrent and predictive validity of the I-Score highlight its limited accuracy in classifying PLHIV based on their intention to adhere to ART. Results on the relationship between intention and medication adherence have been mixed[55] and, therefore, this finding may not be surprising. Arguably, behavioral adherence and viral load outcomes are of greater interest within the context of HIV care, and considering this, our success thresholds were largely met. Notably, with the global sample as well as in the subgroup analyses, 100% of conclusive results were above threshold (i.e., AUC $\geq$ 0.70) for self-reported HIV viral load (collected from the full sample) and plasma viral load test results (collected from a subsample of Montreal participants). These findings are encouraging, given the high variation observed in the ability of self-report measures of adherence to predict viral non-suppression in people with HIV.[9] The subgroup analyses also provide evidence of the cross-cultural validity of our instrument, which is rarely evaluated in adherence-related PROMs, despite the generation of translations.[56]

With an ICC of 0.81, our measure surpassed COSMIN's minimum threshold for adequate reliability and we reported its measurement error (SEM = 0.43), significant because the measurement error of patient-reported outcome measures of medication adherence is rarely provided.[56]

Limitations

The 7-item I-Score's measurement properties were assessed with a limited, albeit diverse, sample of people with HIV, largely from Montreal, Toronto, and Paris. Efforts are currently underway to obtain funding for a Canada-wide study with a more substantial sample of PLHIV and pharmacy refill data for further validation research. Notably, this will help us compensate for many of the inconclusive results obtained in the regression analyses due, in part, to small subgroup sizes.

No global scoring scheme has yet been determined for the measure and it is currently being piloted in HIV care in Montreal as a string of 7 independent items.[57] Likewise, a meaningful cutoff for the response scales to indicate a clinically significant barrier is also not available. These issues limit our instrument's interpretability and will be the focus of future research.

Furthermore, the subgroup analyses suggest that the performance of the French version of the I-Score within Canada is suboptimal for predicting concurrent or future self-reported adherence (but not self-reported or plasma HIV viral load). To better understand this finding, further cognitive testing of the instrument among francophones in Canada may be required.

Finally, the development of this measure began before the emergence of long-acting (LA) ART for the treatment of HIV. LA-ART may resolve a number of adherence barriers for some PLHIV (e.g., daily oral pills as a reminder of living with HIV or as carrying the risk of unintended disclosure of HIV status), while potentially giving rise to new ones (e.g., anxiety around injections).[58] Cognitive and measurement property testing of the instrument with PLHIV on LA regimens may be needed to ensure its applicability to this context of use.

Conclusion

The 7-item I-Score, a patient-reported measure of ART adherence barriers for use in HIV care, has good evidence of content validity, construct validity, and reliability, as well as acceptable measurement error. Its associations with self-reported and plasma viral load, overall, and across a range of sociodemographic groups, appear especially strong. Further research is needed to determine clinically meaningful scoring and cutoff thresholds to facilitate interpretability. Nevertheless, in a context where annual PROM administration is being recommended by HIV clinical guidelines to all PLHIV[59] and no consensus exists on which measures to employ,[60] the I-Score offers an option for use in adherence management. Contrary to other barrier measures,[18] it does not require the admission or presence of missed doses to score highly and may lead to less stigmatizing discussions and more preventative interventions. We expect the results of our clinical pilot study with the I-Score[57] to be informative in these regards.

Ethics statements

Patient consent for publication

Not applicable.

Ethics approval

The I-Score Study obtained ethics approval from the Research Ethics Board (REB) of the McGill University Health Centre (MUHC) in Montreal, Quebec, Canada (original approval received December 1, 2014: 2015-2331, 14-229 PSY). In addition to annual renewals until November 29, 2023, two subsequent amendments were also approved by the MUHC REB (an amendment to the I-Score measure's validation plan: approved January 18, 2022; an amendment for the retrospective analysis of viral load test results of patients with HIV at the Chronic Viral Illness Service of the McGill University Health Centre, waiving the requirement for informed consent: approved September 21, 2023). For the Toronto site (St. Michael's Hospital), REB approval was originally obtained from Unity Health Toronto, Toronto, Ontario, Canada, on November 10, 2016 (REB# 16-104) and renewed until November 10, 2023. For the affiliated French sites, approval was obtained from the REB of the Nantes University Health Centre in Nantes, France, on March 6, 2018 (RC17_0310 N° EUDRACT: 2017-A02460-53; French legislation requires no subsequent annual renewals). The project to meaningfully involve people with HIV in the I-Score Study, in part through a patient advisory committee, was approved by the MUHC REB on September 8, 2015 (project number: 15-188-MUHC).

Funding statement

This work received support from the Canadian Institutes of Health Research (CIHR) HIV Clinical Trials Network (CTN) (CTN283). It was conducted within the program of a SPOR Mentorship Chair in Innovative Clinical Trials awarded by the CIHR to BL (Grant number 383427). It benefited from methodological expertise and funding from the Quebec SPOR Support Unit -McGill Methodological Developments Platform (Grant number M006). It was also supported, in part, by a research grant from the Investigator Initiated Studies Program of Merck Canada Inc. (Grant number IISP-53538), which had no hand in the design, conduct, or writing up of this work. French (France) participation was supported, in part, by funding from MSD Avenir awarded to KL and managed by the AP-HP Foundation (Grant number MSDAVENIR DS-2018-0072). The opinions expressed in this study are those of the authors and do not necessarily represent those of Merck Canada Inc or its affiliates or related companies. BL is supported by two career awards: a Fonds de Recherche du Québec-Santé (FRQ-S) Senior Research Scholar award (#311200) and the LE 250, from Quebec's Ministry of Health for researchers in family medicine. From 2021 to 2023, SV was supported by a CTN Postdoctoral Fellowship Award. DHST is supported by a Tier 2 Canada Research Chair in HIV Prevention and STI Research.

Competing interests statement

BL has received research support and consulting fees from ViiV Healthcare, Merck, and Gilead. KL has received travel support and consulting fees from ViiV Healthcare, Merck, and Gilead. DHST's institution has received research support for investigator-initiated research studies from Abbvie and Gilead, and for participation in industry-sponsored trials from Glaxo Smith Kline. The other authors have no competing interests to declare.

Author contributions

KE and BL designed the project. DL planned and led the associated stakeholder engagement activities, coordinated the project, and conducted the cognitive interviews. SV and KE planned and performed the analyses, with input from BL and DL. BL, DL, DHST and KL contributed to participant recruitment. KE and SV drafted the manuscript. All critically revised the manuscript and approved its final version.

Acknowledgements

The authors warmly thank Dr. Golriz Pahlavan and Alexandre Aslan, for their unwavering belief in and support of this project since its inception; Hayette Rougier for her invaluable help with recruitment and administration of the Paris study site; Francesco Avallone for assistance with qualitative coding of the cognitive interviews; and all the people with HIV, healthcare professionals, and support staff who contributed to this work.

References

1. Public Health Agency of Canada. Estimates of HIV incidence, prevalence and Canada's progress on meeting the 90-90-90 HIV targets, 2020. 2022. Available at: https://www.canada.ca/en/public-health/services/publications/diseases-conditions/estimates-hiv-incidence-prevalence-canada-meeting-90-90-90-targets-2020.html
2. Public Health Agency of Canada. HIV in Canada: 2022 surveillance highlights. 2023. Available at: https://www.canada.ca/en/public-health/services/publications/diseases-conditions/hiv-2022-surveillance-highlights.html
3. Government of Quebec. Portrait épidémiologique des infections transmissibles sexuellement et par le sang, région de Montréal, 2013-2022 Mise à jour incluant les données sur l'infection par le virus de l'immunodéficience humaine, année 2022. Secteur Prévention et Contrôle des maladies infectieuses Direction régionale de santé publique de Montréal CIUSSS du Centre-Sud-de l'Île-de-Montréal, Novembre 2023. Available at: https://santemontreal.qc.ca/fileadmin/fichiers/professionnels/DRSP/sujets-a-z/ITSS/Portrait_epidemiologique-ITSS_Montreal_2023-11.pdf
4. Shubber Z, Mills EJ, Nachega JB, Vreeman R, Freitas M, Bock P, Nsanzimana S, Penazzato M, Appolo T, Doherty M, Ford N. Patient-Reported Barriers to Adherence to Antiretroviral Therapy: A Systematic Review and Meta-Analysis. PLoS Med 2016 Nov;13(11):e1002183.
5. Angel JB, Freilich J, Arthurs E, Ban JK, Lachaine J, Chounta V, Harris M. Adherence to oral antiretroviral therapy in Canada, 2010-2020. AIDS 2023;37(13):2031-2040.
6. Bezabhe WM, Chalmers L, Bereznicki LR, Peterson GM. Adherence to antiretroviral therapy and virologic failure: a meta-analysis. Medicine (Baltimore) 2016;95(15):e3361.
7. Ortego C, Huedo-Medina TB, Llorca J et al. Adherence to highly active antiretroviral therapy (HAART): a meta-analysis. AIDS Behav 2011;15:1381–1396.
8. Fredericksen R, Crane PK, Tufano J, Ralston J, Schmidt S, Brown T, Layman D, Harrington RD, Dhanireddy S, Stone T, Lober W, Kitahata MM, Crane HM. Integrating a web-based, patient-administered assessment into primary care for HIV-infected adults. J AIDS HIV Res 2012;4(2):47-55.
9. Smith R, Villanueva G, Probyn K, Sguassero Y, Ford N, Orrell C, Cohen K, Chaplin M, Leeflang MMG, Hine P. Accuracy of measures for antiretroviral adherence in people living with HIV. Cochrane Database of Systematic Reviews 2022, Issue 7.
10. World Health Organization. Epidemiological fact sheet. HIV statistics, globally and by WHO region, 2023. 2023. Available at: https://cdn.who.int/media/docs/default-source/hq-hiv-hepatitis-and-stis-library/j0294-who-hiv-epi-factsheet-v7.pdf
11. Colasanti J, Kelly J, Pennisi E, Hu YJ, Root C, Hughes D, Del Rio C, Armstrong WS. Continuous Retention and Viral Suppression Provide Further Insights Into the HIV Care Continuum Compared to the Cross-sectional HIV Care Cascade. Clin Infect Dis. 2016 Mar 1;62(5):648-654.
12. Guy R, Mean CV, Wand H, et al. Antiretroviral treatment interruption and loss to follow-up in two hiv cohorts in australia and asia: implications for 'test and treat' prevention strategy. *AIDS Patient Care and STDs*. 2013;27(12):681-691. doi:10.1089/apc.2012.0439
13. Hughes AJ, Mattson CL, Scheer S, Beer L, Skarbinski J. Discontinuation of antiretroviral therapy among adults receiving HIV care in the United States. J Acquir Immune Defic Syndr. 2014 May 1;66(1):80-9.

14. Crespo-Bermejo C, de Arellano ER, Lara-Aguilar V, Valle-Millares D, Gómez-Lus ML, Madrid R, Martín-Carbonero L, Briz V. Persistent low-Level viremia in persons living with HIV undertreatment: An unresolved status. Virulence. 2021 Dec;12(1):2919-2931.
15. Olakunde BO, Ezeanolue EE. The virological consequences of low-level viraemia. The Lancet Global Health. 2022;10(12):1700.
16. Ford N, Geng E, Ellman T, Orrell C, Ehrenkranz P, Sikazwe I, Jahn A, Rabkin M, Ayisi Addo S, Grimsrud A, Rosen S, Zulu I, Reidy W, Lejone T, Apollo T, Holmes C, Kolling AF, Phate Lesihla R, Nguyen HH, Bakashaba B, Chitembo L, Tiriste G, Doherty M, Bygrave H. Emerging priorities for HIV service delivery. PLoS Med. 2020 Feb 14;17(2):e1003028.
17. Lebouché B, Engler K, Lévy JJ, Gilmore N, Spire B, Rozenbaum W, Routy JP. Minimal interference: A basis for selecting ART for prevention with positives. AIDS Care. 2013;25(10):1284-90.
18. Engler K, Toupin I, Vicente S, Ahmed S, Lebouché B. A review of HIV-specific patient-reported measures of perceived barriers to antiretroviral therapy adherence: what themes are they covering? Journal of Patient-Reported Outcomes. 2019;3:37.
19. Chu D, Schuster T, Lessard D, Mate K, Engler K, Ma Y, Abulkhir A, Arora A, Long S, de Pokomandy A, et al. Acceptability of a patient portal (Opal) in HIV clinical care: a feasibility study. J Pers Med. 2021;11:134.
20. Lessard K, Engler K, Toupin I, I-Score Consulting Team, Routy J-P, Lebouché B. Evaluation of a project to engage patients in the development of a patient-reported measure for HIV care (the I-Score Study). Health Expectations. 2019;22:209–225.
21. Engler K, Vicente S, Mate KKV, Lessard D, Ahmed S, Lebouché B. Content validation of a new measure of patient-reported barriers to antiretroviral therapy adherence, the I-Score: results from a Delphi study. Journal of Patient-Reported Outcomes. 2022;6(1):28.
22. Lessard D, Engler K, Vicente S, Bilodeau M, Lebouché B. (2020). Challenges of patient engagement in an HIV clinical research program: a qualitative analysis of stakeholder accounts. Journal of Patient Experience. 2020;7(6):925-930.
23. Lessard D, Vicente S, Keeler P, Lebouché B. A patient's narrative of engaging HIV care: Lessons learned to harness resources and improve access to care. Patient Experience Journal. 2021;8(2):14-17.
24. Engler K, Lènàrt A, Lessard D, Toupin I, Lebouché B. Barriers to antiretroviral therapy adherence in developed countries: a qualitative synthesis to develop a conceptual framework for a new patient-reported outcome measure. AIDS Care. 2018;30(sup1):17-28.
25. Engler K, Ahmed S, Lessard D, Vicente S, Lebouché B. Assessing the content validity of a new patient-reported measure of barriers to antiretroviral therapy adherence for electronic administration in routine HIV care: Proposal for a web-based Delphi study. JMIR Research Protocols. 2019;8(8):e12836.
26. Sabaté E. Adherence to Long-term Therapies: Evidence for Action. 2003 Geneva World Health Organization. Available at: https://www.paho.org/en/documents/who-adherence-long-term-therapies-evidence-action-2003
27. Chan AHY, Cooper V, Lycett H, Horne R. Practical barriers to medication adherence: What do current self- or observer-reported instruments assess? Front Pharmacol. 2020;11:572.
28. Kwan YH, Oo LJY, Loh DHF, Phang JK, Weng SD, Blalock DV, Chew EH, Yap KZ, Tan CYK, Yoon S, Fong W, Østbye T, Low LL, Bosworth HB, Thumboo J. Development of an

item bank to measure medication adherence: systematic review. J Med Internet Res. 2020;22(10):e19089.
29. Rampin R, Rampin V. Taguette: open-source qualitative data analysis. Journal of Open Source Software. 2021;6(68):3522. Available at: https://doi.org/10.21105/joss.03522
30. Prinsen CAC, Mokkink LB, Bouter LM, Alonso J, Patrick DL, de Vet HCW, Terwee CB. COSMIN guideline for systematic reviews of patient-reported outcome measures. Qual Life Res. 2018 May;27(5):1147-1157.
31. Loutfy M, Tharao W, Kazemi M, Logie CH, Underhill A, O'Brien N, Pick N, Kestler M, H Yudin M, Rana J, MacGillivray J, Kennedy VL, Jaworsky D, Carvalhal A, Conway T, Webster K, Lee M, Islam S, Nicholson V, Ndung'u M, Proulx-Boucher K, Carter A, Gormley R, Narasimhan M, Welbourn A, de Pokomandy A, Kaida A; CHIWOS Research Team. Development of the Canadian Women-Centred HIV Care Model Using the Knowledge-to-Action Framework. J Int Assoc Provid AIDS Care. 2021 Jan-Dec;20:2325958221995612.
32. Gorusch RL. Factor Analysis (2nd ed.). 1983. Hillsdale, NJ: Lawrence Erlbaum Associates.
33. Hatcher L. A Step-by-Step Approach to Using the SAS® System for Factor Analysis and Structural Equation Modeling. 1994. Cary, N.C.: SAS Institute, Inc.
34. Harris PA, Taylor R, Minor BL, Elliott V, Fernandez M, O'Neal L, McLeod L, Delacqua G, Delacqua F, Kirby J, Duda SN; REDCap Consortium. The REDCap consortium: Building an international community of software platform partners. J Biomed Inform. 2019;95:103208.
35. Amico KR, Fisher WA, Cornman DH, Shuper PA, Redding CG, Konkle-Parker DJ, Barta W, Fisher JD. Visual analog scale of ART adherence: association with 3-day self-report and adherence barriers. J Acquir Immune Defic Syndr. 2006;42(4):455-9.
36. O'Connor JL, Gardner EM, Esser S, Mannheimer SB, Lifson AR, Telzak EE, Phillips AN; International Network for Strategic Initiatives in Global HIV Trials (INSIGHT) Strategies for Management of Antiretroviral Therapy (SMART) Study Group. A simple self-reported adherence tool as a predictor of viral rebound in people with viral suppression on antiretroviral therapy. HIV Med. 2016;17(2):124-32.
37. Banas K, Lyimo RA, Hospers HJ, van der Ven A, de Bruin M. Predicting adherence to combination antiretroviral therapy for HIV in Tanzania: a test of an extended theory of planned behaviour model. Psychology & Health. 2017;32(10):1249-1265.
38. Castillo-Mancilla JR, Haberer JE. Adherence measurements in HIV: New advancements in pharmacologic methods and real-time monitoring. Curr HIV/AIDS Rep. 2018;15(1):49-59.
39. Mokkink LB, Terwee CB, Knol DL, Stratford PW, Alonso J, Patrick DL, et al. The COSMIN checklist for evaluating the methodological quality of studies on measurement properties: a clarification of its content. BMC Med Res Methodol. 2010;10:22.
40. Nguyen TM, La Caze A, Cottrell N. What are validated self-report adherence scales really measuring?: a systematic review. Br J Clin Pharmacol. 2014;77(3):427-445. doi:10.1111/bcp.12194
41. Byrd KK, Hou JG, Hazen R, Kirkham H, Suzuki S, Clay PG, Bush T, Camp NM, Weidle PJ, Delpino A; Patient-Centered HIV Care Model Team. Antiretroviral adherence level necessary for HIV viral suppression using real-world data. J Acquir Immune Defic Syndr. 2019;82(3):245-251.
42. Panel on Antiretroviral Guidelines for Adults and Adolescents. Guidelines for the Use of Antiretroviral Agents in Adults and Adolescents with HIV. Laboratory Testing. Department of Health and Human Services. 2023. Available

at https://clinicalinfo.hiv.gov/en/guidelines/hiv-clinical-guidelines-adult-and-adolescent-arv/plasma-hiv-1-rna-cd4-monitoring.

43. R Core Team. R: A language and environment for statistical computing. 2022. R Foundation for Statistical Computing, Vienna, Austria. Available at: https://www.R-project.org/.
44. Aguinis H, Vassar M, Wayant C. On reporting and interpreting statistical significance and p values in medical research. BMJ Evidence-Based Medicine. 2021;26(2):39-42.
45. Akoglu, H. User's guide to correlation coefficients. Turkish Journal of Emergency Medicine. 2018;18(3):91-93.
46. Hosmer DW, Lemeshow S, Sturdivant RX. Applied Logistic Regression. Third edition. Wiley; 2013.
47. Swets JA. Measuring the accuracy of diagnostic systems. Science. 1988;240(4857):1285-1293.
48. Gibbons C, Porter I, Gonçalves-Bradley DC, Stoilov S, Ricci-Cabello I, Tsangaris E, Gangannagaripalli J, Davey A, Gibbons EJ, Kotzeva A, Evans J, van der Wees PJ, Kontopantelis E, Greenhalgh J, Bower P, Alonso J, Valderas JM. Routine provision of feedback from patient-reported outcome measurements to healthcare providers and patients in clinical practice. Cochrane Database of Systematic Reviews. 2021;10.
49. Terwee CB, Prinsen CAC, Chiarotto A, Westerman MJ, Patrick DL, Alonso J, Bouter LM, de Vet HCW, Mokkink LB. COSMIN methodology for evaluating the content validity of patient-reported outcome measures: a Delphi study. Qual Life Res. 2018;27(5):1159-1170.
50. Rosenzveig A, Kuspinar A, Daskalopoulou SS, Mayo NE. Toward patient-centered care: a systematic review of how to ask questions that matter to patients. Medicine (Baltimore). 2014;93(22):e120.
51. Finitsis DJ, Pellowski JA, Huedo-Medina TB, et al. Visual analogue scale (VAS) measurement of antiretroviral adherence in people living with HIV (PLWH): a meta-analysis. J Behav Med. 2016;39:1043–1055.
52. Åström, M., Thet Lwin, Z.M., Teni, F.S. et al. Use of the visual analogue scale for health state valuation: a scoping review. Qual Life Res. 2023;32:2719–2729.
53. Wilson IB, Carter AE, Berg KM. Improving the self-report of HIV antiretroviral medication adherence: is the glass half full or half empty? Curr HIV/AIDS Rep. 2009 Nov;6(4):177-86.
54. Schober P, Boer C, Schwarte LA. Correlation coefficients: appropriate use and interpretation. Anesthesia & Analgesia. 2018;126(5):1763-1768.
55. Holmes EA, Hughes DA, Morrison V. Predicting adherence to medications using health psychology theories: a systematic review of 20 years of empirical research. Value in Health. 2014;17(8):863-76.
56. Kwan YH, Weng SD, Loh DHF, Phang JK, Oo LJY, Blalock DV, Chew EH, Yap KZ, Tan CYK, Yoon S, Fong W, Østbye T, Low LL, Bosworth HB, Thumboo J. Measurement Properties of Existing Patient-Reported Outcome Measures on Medication Adherence: Systematic Review. J Med Internet Res. 2020 Oct 9;22(10):e19179.
57. Engler K, Vicente S, Ma Y, Hijal T, Cox J, Ahmed S, Klein M, Achiche S, Pant Pai N, de Pokomandy A, Lacombe K, Lebouché B. Implementation of an electronic patient-reported measure of barriers to antiretroviral therapy adherence with the Opal patient portal: Protocol for a mixed method type 3 hybrid pilot study at a large Montreal HIV clinic. PLoS One. 2021;16(12):e0261006.
58. Nachega JB, Scarsi KK, Gandhi M, et al. Long-acting antiretrovirals and HIV treatment adherence. Lancet HIV 2023;10(5):e332-e342.

59. European AIDS Clinical Society. Use of Patient Reported Outcome Measures (PROMs) in HIV Clinical Care. 2023. Available from: https://eacs.sanfordguide.com/prevention-non-infectious-co-morbidities/eacs-patient-reported-outcome-measures-in-hiv-care
60. Wang Z, Zhu Y, Duan X, et al. HIV-specific reported outcome measures: systematic review of psychometric properties. JMIR Public Health Surveill 2022;8(12):e39015.

Supporting information

S1 File. Cognitive interview schedule for the focus groups.

S1 Table. Descriptive statistics of the I-Score's 7 items at each time point, for the global sample and stratified by sociodemographic group and mode of survey administration.

S2 Table. Descriptive statistics of the five dependent variables at each time point, for the global sample and stratified by sociodemographic group and mode of survey administration.

Figure 1

Click here to access/download;Figure;Fig 1.tif

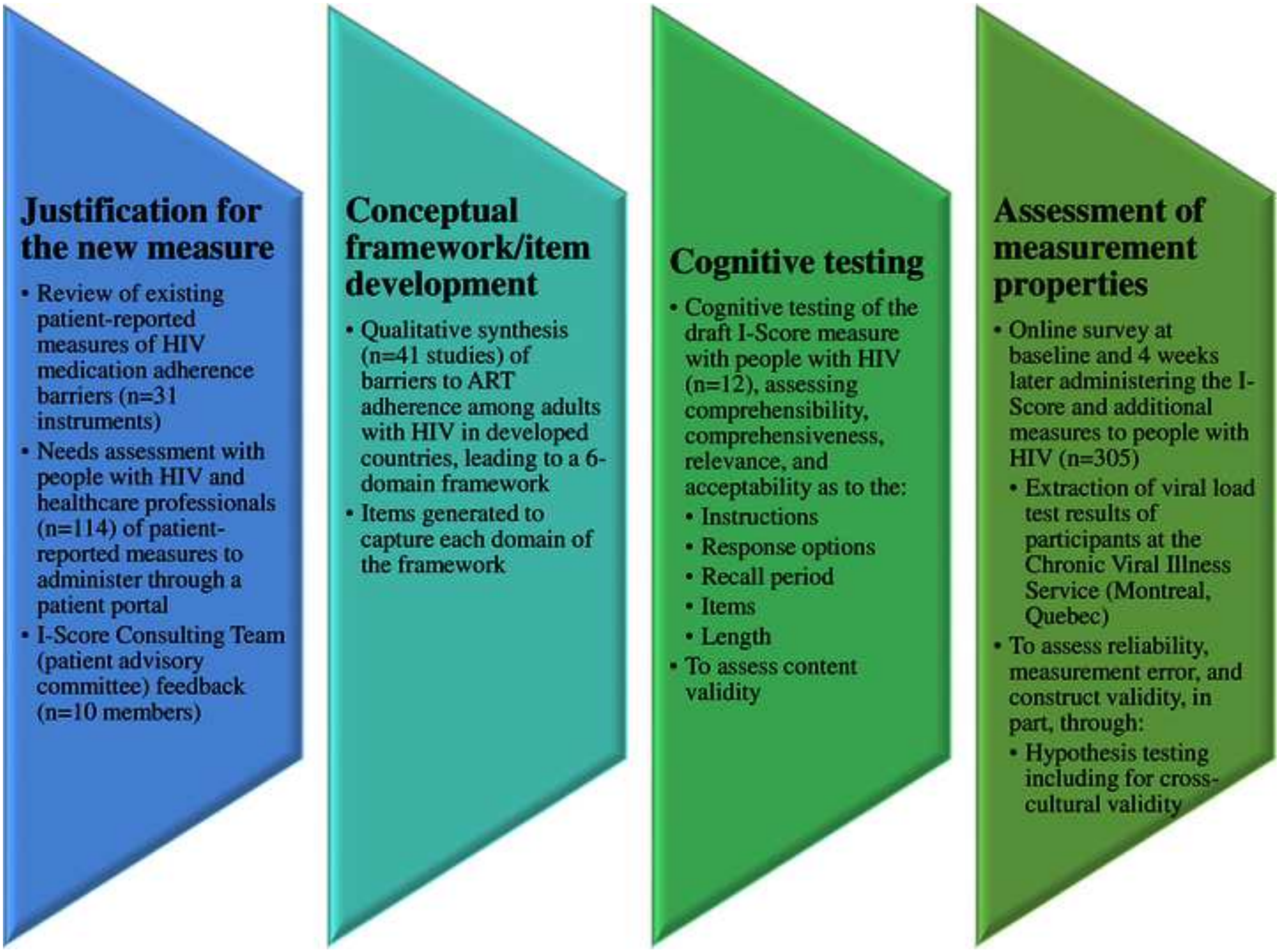

S1 File Interview schedule

Click here to access/download
Supporting Information
S1 File.docx

S1 Table

Click here to access/download
Supporting Information
S1 Table.docx

S2 Table

Click here to access/download
Supporting Information
S2 Table.docx
