# Supplementary material for: A multi-phase project to develop a patient-reported measure of barriers to antiretroviral therapy adherence for use in HIV care: The 7-Item I-Score
Source: PLoS One. 2026 Jan 6;21(1):e0324241. doi: 10.1371/journal.pone.0324241 (PMC12774347; doi:10.1371/journal.pone.0324241)
Supplement: Attachment — Submitted filename: Response to reviewers.docx [file pone.0324241.s010.docx]

September 16, 2025

Alejandro Torrado Pacheco, PhD
Staff Editor
PLOS ONE

**Re: Author response to reviewers of PONE-D-24-21594R1**

Dear Dr. Pacheco and reviewers,

Thank you for the opportunity to further improve the manuscript. We greatly appreciate the reviewers’ positive feedback on the value of our work; it is very validating. Below, we provide a point-by-point response to the proposed areas for improvement. We sincerely hope our changes will be to your satisfaction.

On behalf of the co-authors,

The first author

| **Reviewer comment** | **Author response** |
| --- | --- |
| My biggest question is who the audience is for this manuscript? It is full of technical details, so it would seem that this is for the methodologists. That said, there are descriptions of some of the methods that suggest maybe this is for non-methods people. For instance, in lines 304-310 the description of PCA is probably not needed for a methodologist audience since they should be familiar with PCA and how PCA and rotations are used in survey development. | Thank you for your thoughtful feedback. The level of methodological detail provided was to allow readers and other individuals interested in PROM development to understand the process and rationale as much as possible. However, we agree that the manuscript may have acquired a distracting didactic tone. We have, therefore, revised the paper to remove unnecessary methodological descriptions (see deleted text in paragraphs starting on lines 301, 311, 334, and 346). |
| Furthermore, some of the details feel as though they could probably be moved to a supplement since they don't add too much to the overall story. For instance, Tables 4 and 5 are important, but do they add much? I felt like they could be referenced with a sentence or two in the text and included in the supplement for the supremely interested to see. Changes such as this would greatly help the flow of the manuscript and, I hope, not discourage the non-methodologists from reading this. | Thank you for this comment. We welcome the suggestion to simplify the manuscript. We have relegated Tables 4 and 5 to the supplementary files (i.e. S3 and S4 Tables) and now briefly describe their results in the paragraph starting on line 394. |
| (line 203) Is this supposed to be "...HIV in Canada"? | Yes, thank out for pointing out the typo. We have made the correction. |
| (lines 319-322) Is there any scientific basis for these thresholds or are they based on feels? These are pretty important to your assessments, but I don't find them to be that convincing. I'm not sure there is much you can change, but you might want to make note of this. | Thank you for raising this important point. The thresholds we used for interpreting the areas under the ROC curve (AUCs) are heuristic guidelines rather than values derived from theoretical statistical proofs or comprehensive simulation studies.  As originally presented by Hosmer and Lemeshow in Applied Logistic Regression (2000) [1], the cut-offs were introduced to provide applied researchers with a pragmatic framework for describing model discrimination. Much like the conventional thresholds for Cronbach’s alpha (e.g., 0.7 acceptable, 0.8 good, 0.9 excellent), these values are rules of thumb that have become widely cited and adopted for their simplicity and interpretability, not because of formal statistical justification.  Hosmer & Lemeshow’s thresholds as a rule of thumb are used in a wide range of applied studies across disciplines. For example, Walsh et al. (2022) describe their interpretation of AUC values “as per rule of thumb from Hosmer and Lemeshow” [2]. Similarly, a U.S. government report on criminal justice risk assessment refers to AUC classification “according to the rule of thumb of Hosmer and Lemeshow (2000)” [3]. Other methodological discussions in machine learning and engineering contexts also cite the same framework as a rule of thumb [4,5,6].  Hopefully, this clarifies our choice of thresholds to interpret the AUC’s.  **References**   1. Hosmer DW, Lemeshow S, Sturdivant RX. *Applied Logistic Regression*. 2nd ed. New York: Wiley; 2000. 2. Walsh ME, Ferris H, O’Hanlon S, O’Connor M, Lenehan B. Development of a frailty index in the Irish hip fracture population. *Arch Osteoporos*. 2022;17(1):88. 3. U.S. Department of Justice. *Criminal Justice Risk Assessment: A Review of Instruments and Validity*. Washington, DC: DOJ; 2010. 4. Sun X, Xu W. Fast implementation of DeLong’s algorithm for comparing the areas under correlated ROC curves. *MDPI Aerospace*. 2020;7(11):151. 5. Ke L, Zhang G, He J, Li Y, Li Y, Liu X, Fang P. Pilot Selection in the Era of Virtual Reality: Algorithms for Accurate and Interpretable Machine Learning Models. *Aerospace*. 2023;10(5):394. 6. Yang, S, Berdine, G. The receiver operating characteristic (ROC) curve. The Southwest Respiratory and Critical Care Chronicles. 2017; 5(19):34. |
| (Table 6) I found this table absolutely overwhelming. I'm not quite sure what to make of it and I'm not sure what to suggest. I guess my suggestion would be to create a forest-like plot with the AUCs for all the models (including 95% confidence intervals as error bars). You could color them based on the H and S interpretations if you wish. I can understand the desire to comment on the significant covariates, but I'm not the biggest fan of that. I encourage reporting the entire model, with all odds ratios and 95% CIs. That would have to be a supplementary table (or tables), but maybe you can include the significant variables in the text and then leave the details to the supplement. | We agree that Table 6 is very large and could easily be overwhelming. Thank you for your suggestions. We have split Table 6 into two tables: 1) Table 4, which provides the AUCs for all models and color codes the results based on the Hosmer & Lemeshow + Swets interpretations (see the legend); and 2) S5 Table (a new supplemental table), which displays the odds ratios and 95% CIs of the entire models, highlighting the significant covariates.  Concerning the request to include confidence intervals for the AUCs in the new Table 4, we have decided against this. Xu (2024) [1] shows that DeLong’s widely used non-parametric variance (and covariance) estimates are always positively biased, which in turn biases standard errors and CIs for single or multiple AUC’s. Instead, they recommend resampling methods, including the Monte Carlo bootstrap and the ideal bootstrap, to assess the statistical variability of the AUC. Originally, we used the bootstrap approach to compute confidence intervals for the AUC’s, but the CIs were too large and, therefore, we decided not to report them. Hopefully, this explains our reasons for not reporting CIs in Table 4.  **Reference**   1. Xu J. On the bias in the AUC variance estimate. Pattern Recognit Lett. 2024;178:62-8. |
| (lines 678-680) If 75% was achieved in 100% of analyses, why is the lower AUC < 0.75? Similarly, why does a 50% have an AUC that is < 0.75? Sorry, I guess I am missing something with how these results are reported. | We understand how this could be confusing. For hypothesis (b), our goal was for 75% of analyses (i.e. of AUC’s calculated) for each dependent variable with the global sample to attain an AUC of at least .70. To achieve this, both AUC’s needed to meet the threshold (i.e. the one between the Time 1 I-Score with a given Time 1 dependent variable; and the other with the Time 1 I-Score and the same dependent variable but at Time 2).  For example: (100%; AUC: 0.75-0.80)  100% = the percentage of AUC’s calculated that attained at least .70 (goal: at least 75%)  0.75-0.80 = the range of AUC’s actually obtained  We hope this clarifies the reporting of our results. |
